# Investigating a therapist-guided, parent-assisted remote digital behavioural intervention for tics in children and adolescents—‘Online Remote Behavioural Intervention for Tics’ (ORBIT) trial: protocol of an internal pilot study and single-blind randomised controlled trial

**DOI:** 10.1136/bmjopen-2018-027583

**Published:** 2019-01-03

**Authors:** Charlotte Lucy Hall, E Bethan Davies, Per Andrén, Tara Murphy, Sophie Bennett, Beverley J Brown, Susan Brown, Liam Chamberlain, Michael P Craven, Amber Evans, Cristine Glazebrook, Isobel Heyman, Rachael Hunter, Rebecca Jones, Joseph Kilgariff, Louise Marston, David Mataix-Cols, Elizabeth Murray, Charlotte Sanderson, Eva Serlachius, Chris Hollis

**Affiliations:** 1 Trial Manager, Institute of Mental Health, University of Nottingham, Nottingham, UK; 2 Research Fellow and Trial Therapist, NIHR MindTech Medtech Co-operative, Institute of Mental Health, University of Nottingham, Nottingham, UK; 3 Clinical Psychologist, Department of Clinical Neuroscience, Karolinska Institutet, Stockholm Health Care Services, Stockholm, Sweden; 4 Consultant Psychologist, University College London, Hospital for Children NHS Foundation Trust, London, UK; 5 Senior Research Fellow, University College London, Hospital for Children NHS Foundation Trust, London, UK; 6 Trial Therapist, Division of Psychiatry and Applied Psychology, University of Nottingham, Queen’s Medical Centre, Nottingham, UK; 7 Trial Therapist, University College London, Hospital for Children NHS Foundation Trust, Nottingham, UK; 8 Professor of Health Psychology, Division of Psychiatry and Applied Psychology, Institute of Mental Health, Nottingham, UK; 9 Consultant Psychiatrist, Hospital for Children NHS Foundation Trust, London, UK; 10 Health Economist, Research Department of Primary care and Population health and Priment CTU, University College London, London, UK; 11 Senior Research Associate, Research Department of Primary care and Population health and Priment CTU, University College London, London, UK; 12 Advanced Nurse, Division of Psychiatry and Applied Psychology, University of Nottingham, Queen’s Medical Centre, Nottingham, UK; 13 Principal Research Associate, Research Department of Primary care and Population Health and Priment CTU, University College London, London, UK; 14 Professor of Child and Adolescent Psychiatric Science, Department of Clinical Neuroscience, Karolinska Institutet, Stockholm, Sweden and Stockholm Health Care Services, London, UK; 15 Professor of eHealth and Primary Care, eHealth Unit, Research Department of Primary Care and Population Health, University College London, London, Sweden; 16 Research Clinical Psychologist, University College London, London, UK; 17 Assistant Professor in Child and Adolescent Psychiatry, Department of Clinical Neuroscience, Karolinska Institutet, Stockholm, Sweden; 18 Queens Medical Centre, Professor of Child and Adolescent Psychiatry, Developmental Psychiatry, University of Nottingham, Nottingham, UK

**Keywords:** behaviour therapy, exposure and response prevention, internet, persistent (chronic) motor or vocal tic disorder, tourette’s disorder

## Abstract

**Introduction:**

Tourette syndrome and chronic tic disorder are common, disabling childhood-onset conditions. Guidelines recommend that behavioural therapy should be offered as first-line treatment for children with tics. However, there are very few trained behaviour therapists for tics and many patients cannot access appropriate care. This trial investigates whether an internet-delivered intervention for tics can reduce severity of symptoms.

**Methods and analysis:**

This parallel-group, single-blind, randomised controlled superiority trial with an internal pilot will recruit children and young people (aged 9–17 years) with tic disorders. Participants will be randomised to receive 10 weeks of either online, remotely delivered, therapist-supported exposure response prevention behavioural therapy for tics, or online, remotely delivered, therapist-supported education about tics and co-occurring conditions. Participants will be followed up mid-treatment, and 3, 6, 12 and 18 months post randomisation.

The primary outcome is reduction in tic severity as measured on the Yale Global Tic Severity Scale total tic severity score. Secondary outcomes include a cost-effectiveness analysis and estimate of the longer-term impact on patient outcomes and healthcare services. An integrated process evaluation will analyse quantitative and qualitative data in order to fully explore the implementation of the intervention and identify barriers and facilitators to implementation. The trial is funded by the National Institute of Health Research (NIHR), Health Technology Assessment (16/19/02).

**Ethics and dissemination:**

The findings from the study will inform clinicians, healthcare providers and policy makers about the clinical and cost-effectiveness of an internet delivered treatment for children and young people with tics. The results will be submitted for publication in peer-reviewed journals. The study has received ethical approval from North West Greater Manchester Research Ethics Committee (ref.: 18/NW/0079).

**Trial registration numbers:**

ISRCTN70758207 and NCT03483493; Pre-results.

**Strengths and limitations of this study**

- This study is a 10-week, parallel-group, single blind, non-commercial, randomised controlled superiority trial with an internal pilot; the first large trial for internet-delivered therapy for tics in the UK.
- The study design and methodology are based on an initial pilot study of internet-delivered exposure response prevention for tics conducted in Sweden.
- The protocol was created with a multidisciplinary team of experts including, healthcare professionals, patient and public involvement members, and health service researchers.
- This trial is only available to English-speaking participants with access to a computer/internet.

## Introduction

Tourette syndrome (TS) and chronic tic disorder (CTD) are common, disabling, childhood-onset conditions affecting up to 1% of young people and are associated with significant distress, psychosocial impairment and reduced quality of life.^1^ The majority of patients additionally experience comorbidities such as attention-deficit hyperactivity disorder (ADHD), obsessive-compulsive disorder (OCD), depression, anxiety and self-injurious behaviour.^2^ Evidence-based interventions for the treatment of tics in children and adolescents with TS include pharmacological treatment and behaviour therapy.^1 3–5^ European clinical guidelines^3^ and a National Institute of Health Research Health Technology Assessment Evidence Synthesis^1^ recommend that behaviour therapy should be offered as a first-line intervention for tics in children and adolescents in a stepped-care approach.

Despite these recommendations, only one in five young people with TS are currently able to access behaviour therapy for tics, while around 50% receive medication,^6^ which is associated with a significant risk of adverse effects including weight gain and sedation.^4 5^ Furthermore, those young people who manage to access behaviour therapy typically receive four or fewer face-to-face therapy sessions,^6^ less than half the number recommended.^7^ Commonly used behaviour therapies include *habit reversal training (HRT)*, whereby patients are taught to implement and maintain an action referred to as a ‘competing response’ when a premonitory urge to tic is experienced (the urge to tic often felt before the tic is expressed); *comprehensive behavioural intervention for tics (CBIT)*, which includes HRT and adds additional elements of relaxation training, functional analyses (identification of situations which could exacerbate tics) and social support; and *exposure and response prevention (ERP)*, where patients tolerate premonitory urge sensations and learn from their therapist how to resist their tics.

The effectiveness of behavioural therapy for reducing tics has been well established,^1^ with systematic reviews demonstrating a similar magnitude of effect for HRT/CBIT compared with pharmaceutical interventions.^1 4^ A recent trial compared 10 weeks of behavioural therapy (either HRT or ERP) with education and pharmacological treatment and found the behavioural therapy to be as effective as pharmacological treatment for reducing tic severity, with both being superior to education.^8^ Additionally, although there is a lack of evidence from randomised controlled trials (RCTs) for ERP, the evidence from available studies suggests both HRT and ERP may be at least equally effective in reducing tics, with some evidence showing a tendency to favour ERP.^3^ The effectiveness of these behavioural interventions in conjunction with the lack of side effects compared with pharmacotherapy supports the use of behaviour therapy as a first-line intervention. However, there is a lack of trained therapists across many European countries, leaving many patients unable to access evidence-based behaviour therapy. This need for improved access to therapy was highlighted in a recent study whereby families of children with TS noted the struggle to access limited behavioural therapy resources, despite 76% of parents saying they would want behavioural therapy to be available for their child.^6^

Over the last decade, internet-delivered cognitive-behavioural therapy (ICBT) has developed, enabling the delivery of effective, but less therapist intensive, cognitive and behavioural interventions remotely, which can increase the availability of evidence-based treatments. Across diagnostic conditions, studies have not only shown efficacy of ICBT compared with no-treatment control conditions, but meta-analyses comparing face-to-face delivered CBT with ICBT have demonstrated comparable results in terms of symptom reduction for adults with mental health problems,^9 10^ which could also result in 50% cost savings.^10^ However, therapist guidance during ICBT has been shown to be an important contributing factor in determining outcome.^11^ Although self-guided ICBT is superficially attractive, given the very low costs of implementation, research evidence demonstrates very low adherence rates.^12 13^

Recently, researchers at the Karolinska Institutet, Sweden, have developed a technical platform for ICBT called BIP (*Barninternetprojektet*, Swedish for *Child Internet Project*; http://www.bup.se/bip). The platform has been used to deliver therapist-supported ICBT for a range of different conditions, including phobia,^14^ anxiety^15^ and OCD.^16^ The findings support the use of therapist-guided ICBT using the BIP system; however, there is little research evidence with regards to the effectiveness of ICBT and TS. In a recent systematic review of digital health interventions, Hollis and colleagues^17^ found the majority of interventions have been designed to help children and young people at risk for developing, or with a diagnosis of, an anxiety disorder and/or depression, with areas such as TS being largely overlooked. Innovations in behavioural therapies for tics include delivery of CBIT treatment remotely via video over the internet with a therapist. Two pilot RCTs have compared CBIT delivered remotely via videoconference to traditional face-to-face delivery in children with TS. Results showed significant tic reduction for both groups, with no difference between the modes of delivery.^18 19^ The method of delivering CBIT was rated as highly acceptable by the participants.^18^

One Swedish pilot study, on which this current trial is based, has used the BIP platform to deliver ICBT with remote therapist support. The study compared tic reduction in children aged 8–16 years randomised to receive either ERP or HRT delivered online via the BIP platform.^20^ Participants in both groups showed improvement after treatment completion, but only participants in the ERP arm showed significant reduction on the primary outcome measure Yale Global Tic Severity Scale (YGTSS) total tic severity score. The gains were maintained for at least 12 months after the end of treatment. Although this study was not designed or powered as a definitive efficacy trial, these findings show that ERP treatment delivered online via BIP is acceptable to participants and shows promise in reducing tics. Furthermore, the study reported no severe adverse events. There were no drop-outs and no data loss for the primary outcome, and 83% of parents and children rated the treatment as good or very good, indicating the trial was highly acceptable to patients in Sweden.

However, the evidence available for the BIP treatment programmes has all been gathered in Sweden and we have limited experience of delivering therapist supported online ERP to children and adolescents in the UK. There is evidence that uptake and use of digital health interventions is highly context dependent,^21 22^ and it would therefore be unwise to assume that a delivery package that works in Sweden will work equally well in the UK.

The overarching aim of the Online Remote Behavioural Intervention for Tics (ORBIT) trial is to evaluate the clinical and cost-effectiveness of BIP TIC, a therapist-guided, parent-assisted ERP programme for tics in young people with TS in the UK.

Specific objectives are to

1. Optimise the design and delivery of BIP TIC in partnership with young people with TS, carers and healthcare professionals (HCPs) to maximise acceptability, effectiveness and long-term uptake.
2. Undertake an internal pilot to assess whether recruitment, engagement with the intervention and retention to the trial outcomes are sufficient to allow the trial to progress and provide a definitive answer on effectiveness.
3. Determine the clinical effectiveness of BIP TIC compared with online tic-related education in reducing tics, as measured by the masked assessor-rated YGTSS total tic severity score (primary outcome).
4. Evaluate the cost-effectiveness of BIP TIC.
5. Estimate the longer-term impact on patient outcomes and health service costs.
6. Conduct a process analysis in line with Medical Research Council (MRC) guidelines for complex interventions to determine barriers and facilitators to implementation.

The primary hypothesis is that the BIP TIC therapist-guided remote digital ERP intervention will be superior to a comparator intervention of therapist-guided online tic-related education in reducing tics, as measured by the masked assessor-rated YGTSS total tic severity score.^23^

## Methods and analysis

### Trial design

**Figure 1:**
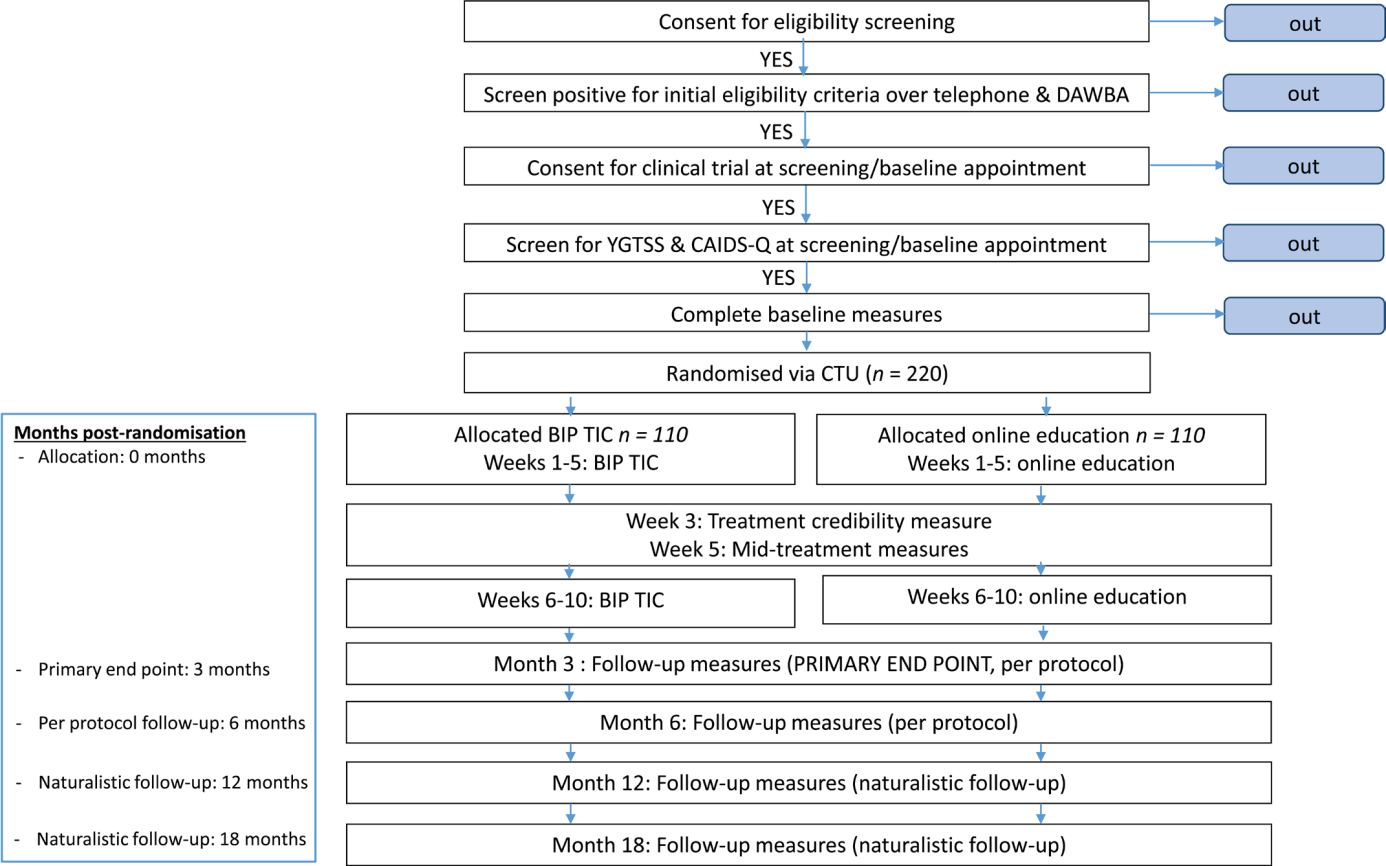
Schematic flow diagram of trial design. CAIDS-Q, Child and Adolescent Intellectual Disability Screening Questionnaire; CTU, Clinical Trials Unit; DAWBA, Development and Wellbeing Assessment; YGTSS, Yale Global Tic Severity Scale.

**Figure 2:**
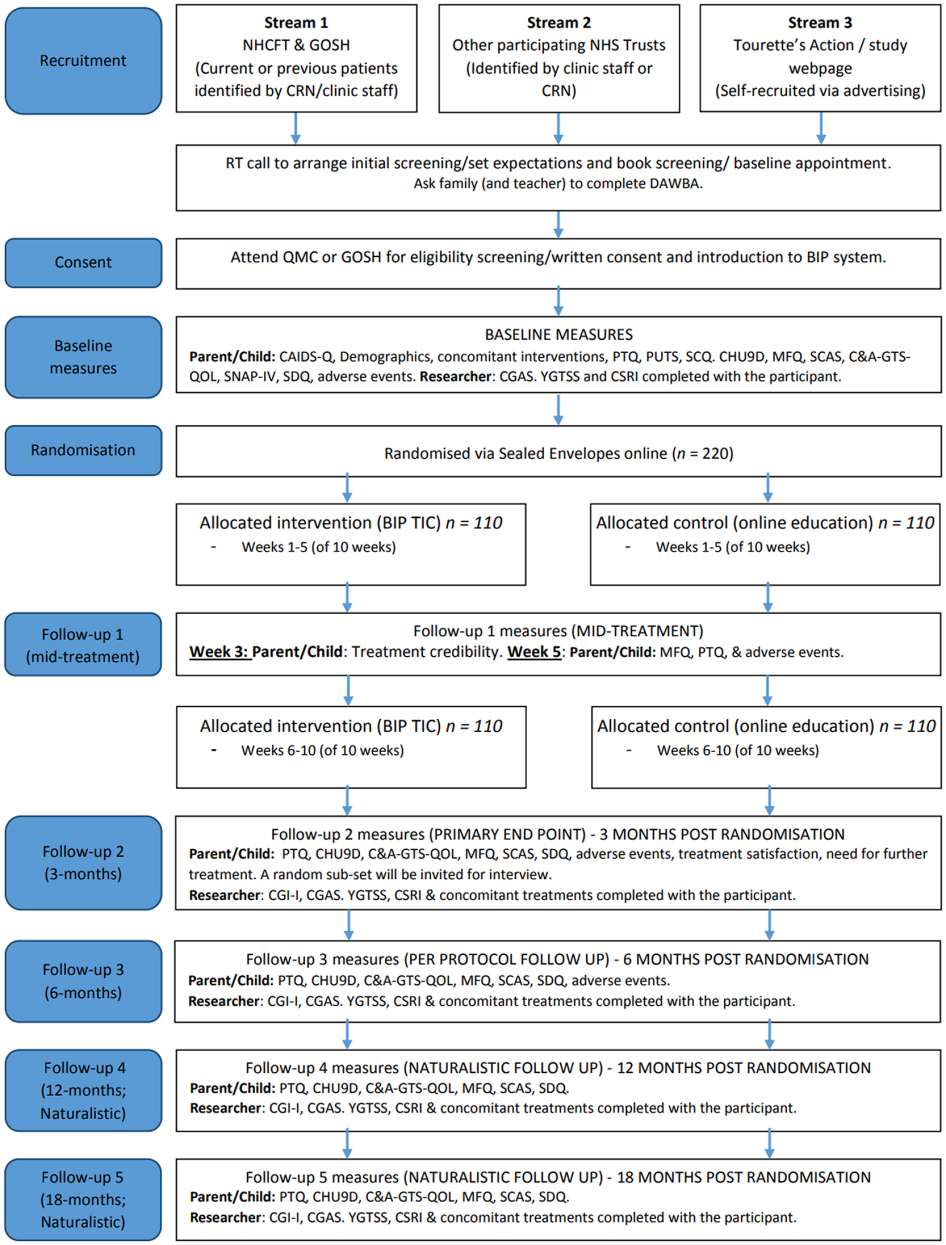
Participant flow chart. Note: The CSRI includes a measure of school attendance. The YGTSS includes both a measure of severity and impairment. C&A-GTS-QOL, Child and adolescent version of the Gilles de la Tourette Syndrome Quality of Life Scale; CAIDS-Q, Child and Adolescent Intellectual Disability Screening Questionnaire; CGAS, Children’s Global Assessment Scale; CGI-I, Clinical Global Impressions-Improvement; CHU9D, Child Health Utility 9D; CSRI, Client Service Receipt Inventory; DAWBA, Development and Wellbeing Assessment; MFQ, Mood and Feelings Questionnaire; PTQ, Parent Tic Questionnaire; PUTS, Premonitory Urge for Tics Scale; RT, research team; SCAS, Spence Child Anxiety Scale; SCQ, Social Communication Questionnaire; SNAP-IV, Swanson, Nolan and Pelham Rating Scale; SDQ, Strengths and Difficulties Questionnaire; YGTSS, Yale Global Tic Severity Scale.

The design is a 10-week, parallel-group, single-blind, non-commercial, randomised controlled superiority trial with an internal pilot. Participants will be randomised to receive 10 weeks of online, remotely delivered, therapist-supported ERP for tics, or online, remotely delivered, therapist-supported education for tics. Participants will be followed up mid-treatment, and at 3, 6, 12 and 18 months post randomisation. Up until the 6-month follow-up participants are encouraged not to change medication or start alternative therapies for tics. Months 12 and 18 are naturalistic follow-up points where participants are free to access any treatments in accordance with standard practice recommended by their usual treating clinician. A subsample of participants and parents will be purposively selected to participate in process evaluation interviews after the 3-month follow-up. Interviews will also be conducted with therapists, clinicians recruiting to the study and commissioners. The process evaluation will also link data on usage of the intervention with outcomes. A schematic diagram of the study design is shown in figure 1, and a more detailed participant flow is shown in figure 2.

An internal pilot will be conducted until the end of the ninth month to determine whether recruitment, engagement with the intervention and retention to the trial are sufficient to allow the trial to progress, the stop/go rules for proceeding are

1. The study needs to have recruited 30% of the sample (66 participants).
2. At least 60% of participants need to have completed the intervention (defined as the child/adolescent completing at least their first four chapters).
3. 80% of participants who have reached the relevant time window need to have completed the primary outcome measure.

The trial was prospectively registered with ISRTCN (ISRCTN70758207) and ClinicalTrials.gov (NCT03483493).

### Setting

The intervention will be remotely delivered from two national centres for treating tics in the UK (Queen’s Medical Centre, Nottingham, and Great Ormond Street Hospital, London), serving geographically and demographically diverse populations. However, multiple Patient Identification Centres (PICs) will be involved in recruiting participants into the two research sites.

### Recruitment and eligibility

#### Participant identification

Participants will be identified and recruited by either (1) clinic staff or clinical study officers at PICs across National Health Services (NHS) Trusts in England; or (2) the two study sites (Nottingham and London) or (3) via Tourettes Action (tic disorder charity) website, our study website or social media, based on the following criteria:

#### Inclusion criteria

1. Aged 9–17 years.
2. Suspected or confirmed TS or chronic tic disorder.
  - Including moderate/severe tics: Total Tic Severity Score >15 on the YGTSS; TTSS score >10 if motor or vocal tics only.
3. Competent to provide written, informed consent (parental consent for child aged <16 years).
4. Broadband internet access and regular PC/laptop/Mac user, with mobile phone SMS.

#### Exclusion criteria

1. Receipt of/engaged in structured behavioural intervention for tics (eg, HRT/CBIT or ERP) within the last 12 months.
2. Change to medication for tics (start or stop) within the previous two months.
3. Diagnoses of alcohol/substance dependence, psychosis, suicidality or anorexia nervosa.
4. Moderate/severe intellectual disability.
5. Immediate risk to self or others.
6. Parent or child not able to speak or read/write English.

Patients will be provided with a brief information sheet and a ‘consent to contact’ (C2C) form: for NHS-referred patients, these forms will be given by a member of the usual care team (or CSO); for self-recruited patients, these will be completed online.

### Initial screening

On receipt of a C2C, a member of the research team will undertake a telephone screening interview with the potential participant to determine basic eligibility. Potential participants who meet the initial eligibility requirements will be invited to attend a face-to-face screening/baseline appointment held at one of the two study sites. All participants will be reimbursed for their travel costs to attend this appointment.

Before participants attend the face-to-face screening/baseline appointment parents will be asked to complete an online parent and teacher Development and Wellbeing Assessment (DAWBA)^24^ (see secondary outcomes for further details). Completion of the parent DAWBA is a requirement for enrolment into the trial; findings from the DAWBA will be used to exclude people who score highly on self-harm, psychosis, anorexia nervosa or suicidality. It is the parent’s decision to involve the teacher in completing the teacher DAWBA.

### Face-to-face screening appointment

Potential participants found eligible from the initial telephone screening will be invited to the face-to-face screening/baseline appointment. Consent will be taken at this appointment by a researcher/assessor trained in Good Clinical Practice (GCP), further details about which are listed under ‘ethics and dissemination’. Participants will be provided with information sheets which have been developed with our patient and public involvement (PPI) group. Families will be encouraged to ask the research team any questions. Where the participant is a child (under 16 years), an age-appropriate participant information sheet will be provided.

A trained assessor will conduct the YGTSS^23^ assessment to determine the presence of tics (inclusion criteria) and the Child and Adolescent Intellectual Disability Screening Questionnaire (CAIDS-Q)^25^ to screen for intellectual disability (exclusion criteria). Details of the YGTSS are described under the primary outcome.

Patients who are not eligible because they have recently changed their tic medication (within 2 months), or received a behavioural therapy for tics within the specified exclusion time frame (12 months), may be eligible for re-screening at a later date when these time exclusions have passed. Patients who meet the eligibility criteria, after completion of all screening assessments, will be asked to complete baseline measures and will be introduced to the BIP system.

### Interventions

The BIP treatment programmes are delivered via a secure internet platform that enables presentation of different treatment content to different patient populations. Patients log in via personal user names and passwords to access the treatment content which usually includes text, pictures, instructional videos and worksheets (see link for video of the intervention https://vimeo.com/294763841/628b99ddfb). The treatment content for both groups is presented in chapters, like a self-help book.

Both the ERP (intervention) and the education (active control) are delivered via the BIP platform. The interventions consist of 10 separate child-chapters and parent/carer-chapters which are designed to be delivered over 10 weeks, with access to therapist support during this time.

The child/young person and their parent/carer are provided with their own separate login to the BIP platform to access the relevant child or parent/carer chapters. The first four chapters cover the main content of the treatment; completion of these four child chapters is a minimum requirement to meet treatment completion criteria.

Both children and parents/carers have regular contact with a therapist during the 10 weeks via messages that can be sent inside the treatment platform (resembling an email) or telephone if needed. The therapist can also directly comment on exercises that the participant has been working on, and give specific feedback to motivate the patient. The participant typically has contact with the therapist several times a week, although the contact is asynchronous. The therapist is available to support the participants during these 10 weeks, although support may be delivered over a 12 week period if necessary to allow for breaks during holiday and festive periods. Access to the BIP treatment programme is granted for 1 year after the 10 weeks of therapist-guided treatment, with the remaining access to the BIP platform being unsupported. The therapist manuals for both interventions were written by the co-applicants.

### Therapist-guided, internet-delivered ERP Intervention (BIP TIC; experimental group)

The treatment consists of evidence-based interventions adapted from previously published treatment manuals on ERP and established behavioural intervention for tics protocols.^26 27^ During the treatment, participants are instructed to practice suppressing their tics: this is known as ‘response prevention’. Then, with the help of their parent/carer, the participant is instructed to provoke premonitory urges and control all of their tics: this is known as ‘exposure’. Through treatment, the child gains mastery in tolerating the urge, controlling the tics and is able to do so for increasingly longer periods of time. The participants also receive detailed education about tics and strategies to promote tic management at school.

The parent modules contain information regarding parent coping strategies, how to support their child in working with BIP and functional analysis relating to tics.

### Therapist-guided, internet-delivered education (active control group)

An active comparator intervention was chosen to ensure that all participants still received an internet-delivered therapist-guided intervention over and above what would typically be received in standard care. Education about tics (without a specific focus on training behavioural strategies to reduce tics) was chosen to enable a comparison to previous trials of face-to-face behaviour therapy for tics.^28^

The comparator intervention was developed for the trial and consisted of educational information about TS and co-occurring conditions. The material was adapted and updated from the educational intervention used in two previous RCTs.^26 28^

The chapters have been matched in length to that of the behavioural intervention. The comparator intervention reviews the definition of tics, natural history, common presentations, prevalence, aetiology, risks and protective factors and strategies for describing tics to other people, and so on. Problem-solving and development of expertise in tic disorders is emphasised. The parent modules emphasise parent coping strategies and self-care in caring for a child with tic disorder. There is no information on tic control within the comparator intervention.

### Start of treatment

The participant will agree a start date for commencing therapy with their assessor at the screening/baseline appointment (typically within 24–48 hours of the baseline appointment). The participant will be reminded that it is important to log-on and start the therapy on the allocated date. At the start, the therapist will release the first two chapters of the intervention for the participants to complete and monitor their progress, sending reminders and feedback where necessary. If the participant does not start the intervention at the agreed start time, the 10-week start date does not alter.

All therapists will undergo training delivered by a supervisor (TM or JK) before commencing either intervention. Therapists will be closely supervised by TM or JK to ensure adherence to protocol. The team from the Karolinska Institutet will provide technical support throughout the trial.

### Measures and outcomes

#### Primary outcome

The primary outcome is the severity of tics as measured by the TTSS (0–50) on the YGTSS.^23^ The primary end point is 3 months post randomisation. The TTSS combines separate scales for motor tics (0–25) and for vocal tics (0–25). The primary outcome measure (TTSS) will be measured at baseline (pre-intervention), 3 months, 6, 12 and 18 months post randomisation.

The YGTSS is administered by a blinded assessor as an investigator-based semistructured interview focusing on motor and vocal tic frequency, severity and tic-related impairment over the previous week. The baseline assessment will be conducted face-to-face at the baseline/screening appointment. When possible, the follow-up assessments will be conducted by the same blinded assessor that conducted the baseline assessment via videoconference call/WebEx or telephone. With the participants’ permission, all YGTSS assessments will be recorded for quality checks.

All assessors/researchers will be trained in conducting YGTSS by a clinical expert (TM). Agreement with the expert rater will be established at the start of the trial and monitored every 6 months. Assessors have to be within 15% of the expert rater (TM) for the Total Motor Tic score, the Total Vocal Tic score and the Total Tic Score on three YGTSS recordings at baseline and four randomly selected recordings at follow-up. Assessors who do not meet this threshold of agreement will be re-trained.

#### Secondary outcomes

A flow chart of study assessments is shown in table 1 which documents the time point for completion and the informant. See also figure 2.

**Table 1:** Schedule of assessments

Months post randomisation	0	0	1	1	3	6	12	18	Completed by
Time point	Telephone screen	Baseline assessment	Mid-treatment (3 weeks)	Mid-treatment (5 weeks)	Primary end point	6 months	12 months	18 months	
Screening for eligibility	X	X							R
SDQ & DAWBA (conducted post telephone screen and prior to baseline)	X								P & T
YGTSS TTSS		X			X	X	X	X	R
YGTSS impairment		X			X	X	X	X	R
CAIDS-Q		X							P/R
SCQ		X							P
SNAP-IV		X							P
Demographics		X							R
PUTS		X							C
PTQ		X		X	X	X	X	X	P
CGAS		X			X	X	X	X	R
CHU9D		X			X	X	X	X	P
Modified CSRI (including school attendance)		X			X	X	X	X	P/R
MFQ (self-report)		X		X	X	X	X	X	C
SCAS (Self-report)		X			X	X	X	X	C
C&A GTS-QOL		X			X	X	X	X	C
Adverse effects/side effects		X		X	X	X			P/C
Concomitant interventions		X			X	X	X	X	P/R
SDQ (P)					X	X	X	X	P
CGI-I					X	X	X	X	R
Treatment credibility			X						P/C
Treatment satisfaction					X				P/C
Need for further treatment					X				P/C
Interview (process evaluation)					X				

C&A-GTS-QOL, Child and adolescent version of the Gilles de la Tourette Syndrome Quality of Life Scale; CAIDS-Q, Child and Adolescent Intellectual Disability Screening Questionnaire; CGAS, Children’s Global Assessment Scale; CGI-I, Clinical Global Impressions- Improvement; CHU9D, Child Health Utility 9D; CSRI, Client Service Receipt Inventory; DAWBA, Development and Wellbeing Assessment; MFQ, Mood and Feelings Questionnaire; P, parent; PTQ, Parent Tic Questionnaire; PUTS, Premonitory Urges for Tics Scale; R, researcher; SCAS, Spence Child Anxiety Scale; SCQ, Social Communication Questionnaire; SDQ, Strengths and Difficulties Questionnaire; SNAP-IV; Swanson, Nolan and Pelham Rating Scale; T, teacher; TTSS, Total Tic Severity Scale; YGTSS, Yale Global Tic Severity Scale.

- *YGTSS: impairment scale* 23: The impairment scale (0–50) forms part of the YGTSS described above. The rating focuses on distress and impairment experienced in interpersonal, academic and occupational domains.
- *Parent Tic Questionnaire (PTQ)* 29: The PTQ assesses the number, frequency and intensity of motor and vocal tics.
- *Clinical Global Impressions Scale (CGI) improvement* 30: The CGI improvement provides an overall clinician-determined summary measure of symptom improvement.
- *Children’s Global Assessment Scale (CGAS)* 31: The CGAS is a 0–100 scale that integrates psychological, social and academic functioning in children as a measure of psychiatric disturbance.
- *Strengths and Difficulties Questionnaire (SDQ—parent completed)* 32: The SDQ is a brief measure of behavioural and emotional difficulties. The SDQ will be completed by parents as part of DAWBA at baseline and at follow-up via an online platform.
- *The Mood and Feelings Questionnaire (MFQ; Child completed version)* 33: The MFQ reports depressive symptoms as specified by the DSM-III-R diagnostic criteria for major depression.
- *Spence Child Anxiety Scale (SCAS—*s*elf-report)* 34: The SCAS is a self-report measure that evaluates symptoms relating to separation anxiety, social phobia, OCD, pain, agoraphobia, generalised anxiety and fears of physical injury.
- *Child Health Utility 9D (CHU9D; parent and child completed versions)* 35: The CHU9D is a paediatric quality of life measure for use in healthcare resource-allocation decision making.
- *The Child and Adolescent Gilles de la Tourette Syndrome—Quality of Life Scale (C&A-GTS-QOL*)^36^: The C&A-GTS-QOL is a disease-specific measure of health-related quality of life designed for children and adolescents with TS.
- *Client Service Receipt Interview (CSRI)* 37: The CSRI is a flexible research instrument developed to collect information on service receipt, service-related issues and income. A modified version of the CSRI will be completed which also combines elements of the Child and Adolescent Service Use Schedule (CA-SUS).^38^
- *Adverse events (side effects)*: Adverse events/side effects will be recorded on a modified version of the side effects scale developed by Hill and Taylor.^39^

The abovementioned scales all have established reliability, validity and history of use in clinical and research settings. Some further measures were developed by the research team for the purpose of this study; these include

- *Treatment credibility*: The questionnaire consists of two items which ask how suitable the child and parent believe the internet treatment is for managing tics, and how much improvement they expect from the treatment.
- *Treatment satisfaction and need for further treatment:* Seven items ask about the child’s/parent’s satisfaction with the treatment and an additional question asks whether they feel more tic treatment is required.
- *Concomitant interventions:* To assess what other treatments/interventions the child/young person is accessing during the study period, parents will be asked to complete a short questionnaire documenting other interventions (including medication) in progress.

#### Additional baseline measures

To further characterise the sample at baseline, the following measures will also be recorded:

- *Demographics questionnaire*: Records the child’s age, gender, ethnicity, parental education/occupation, list of the child’s current diagnoses and interventions (including medications) and general practitioner (GP) and school details.
- *Social Communication Questionnaire (SCQ)* 40: The SCQ is a screening tool for autism spectrum disorder (ASD).
- *Premonitory Urge for Tics Scale (PUTS)* 41: The PUTS is a self-report instrument that measures premonitory urges in tic disorders.
- *Swanson, Nolan, and Pelham Rating Scale (SNAP-IV)* 42: The SNAP-IV is a behavioural rating scale that includes the core symptoms of ADHD and oppositional-defiant disorder (ODD).

#### Process evaluation

The process evaluation will follow the MRC guidelines for evaluating the implementation of complex interventions.^43 44^ It will explore the fidelity and implementation of intervention, and make recommendations for adaptations. It will also examine the potential mechanisms underlying participant behaviour change and probe for unexpected consequences.

Interviews will be conducted with therapists and their supervisors supporting the intervention (target n>5), clinicians recruiting to the study (n >10), children (n>20 in each arm) and parents of children in both arms (n>20 in each arm). Online feedback from participants will also be analysed together with indices recorded as part of the BIP system such as total therapist time, number of chapters clicked through and number of patient log-ins.

#### Patient and public involvement

A group of PPI members was created to shape and guide the study. The group comprises four parents and four children and young people with a diagnosis of TS, plus two PPI members with previous experience of large studies. The design of the trial and its dissemination will also have been shaped by PPI in the following ways:

- Reviewing and selection of outcome measures.
- Co-developing of study materials and recruitment processes.
- Reviewing the interventions and providing feedback on all chapters.
- Attending ongoing trial team meetings to provide PPI perspective and assist with troubleshooting (eg, recruitment and retention).
- Guiding interview topics and shaping questions for process evaluation.
- Co-creating lay summaries and other dissemination materials.

The PPI group mainly contributes remotely, in order to enable involvement from members who are not geographically close. Face-to-face input occurs at key points, for example, attendance at trial team meetings.

#### Sample size

To detect a clinically important average difference of 0.5, of an SD between intervention and comparator with 90% power at p<0.05 (two-sided), after allowing for 20% dropout, requires a total sample size of 220 participants. A systematic review found the average estimate for the SD of the YGTSS (TTSS) from 19 trials of behavioural intervention for tics was 6.6.^17^ Thus, the trial is powered to detect an average difference of 3.3 on the YGTSS.

Recruitment is planned to be conducted within an 18-month period. We are not aware of any other competing studies that are in progress or planned that would affect recruitment. Input received from young people and carers in developing this trial indicates that participation in the trial will be attractive as it guarantees access to an intervention. Our national recruitment network of specialist (PIC) clinics, including multiple community child and adolescent mental health services and paediatric clinics and the charity Tourettes Action, have the capacity to meet the recruitment target within the time window. Recruitment figures will be monitored monthly by the trial manager (CLH) and any shortfall will be reported to the project management group (PMG).

#### Randomisation

Randomisation will be conducted using the Sealed Envelope online randomisation system and managed by Priment Clinical Trials Unit. Participants will be randomised online by the researcher/assessor. Randomisations will be distributed equally between the two arms (ratio 1:1) and stratified by study site using block randomisation with varying block sizes. The researcher will remain blind to the treatment allocation, and the therapist will be notified of the treatment allocation. Therapists will set up participants’ treatment on the BIP system.

All participants receive the same outcome measures so assessors will not know which group the participant is in by the measurements. If an assessor becomes unblinded, subsequent assessments for that participant will be conducted by a different assessor (blind to arm allocation) where possible. In case of a medical emergency, participants will be able to disclose to the treating physician (eg, GP) what treatment they received without unblinding the researchers. Outcome assessors will guess the treatment allocation after each assessment, to be able to calculate if the guesses are better than random.

#### Data management and analysis

Participants who do not complete therapy or measures at a given time point will still be followed up until the end of the trial unless they inform the research team they would like to withdraw from the trial. Participants will be reimbursed with £20 vouchers for completing measures at baseline and each follow-up time point, except the mid-treatment follow-ups (3 and 5 weeks).

All aspects of data management of the study will comply with the UK Data Protection Act 1998/General Data Protection Regulation, Priment’s standard operating procedures and GCP. Outcome measures will be stored in BASS, a rating platform stored on a Swedish server. Participant-completed outcome measures are directly entered into an online database by the participant. Assessor/researcher-completed measures may initially be entered on a paper case report form or directly into the database. Data extraction will be performed within the UK.

#### Statistical analysis

All baseline variables will be summarised by randomised group. Categorical data will be reported as frequencies (%) and continuous data will be reported as mean (SD) unless skewed then they will be reported as median (IQR). No statistical tests will be carried out comparing baseline variables. The primary outcome (TTSS of the YGTSS at 3 months post-randomisation) will be analysed using a linear regression model, including baseline TTSS and centre (the stratification variable). If the assumptions of linear regression are violated, another suitable method will be used. Primary analyses will be performed using intention-to-treat principles. Predictors of missingness will be examined. If any are found, they will be included in statistical models in a supportive analysis. There are no planned subgroup analyses.

Secondary outcomes will be analysed using similar statistical methods as the primary outcome. Continuous outcomes will be analysed with linear regression. Dichotomous outcomes will be analysed using logistic regression. Results will be presented as estimated differences or ORs as appropriate, with accompanying 95% CI. P values will not be reported for secondary outcomes.

#### Economic analysis

The primary cost-effectiveness analysis for the within-trial evaluation will be the mean incremental cost per point change in YGTSS of treatment as usual (TAU) plus BIP TIC compared with TAU plus online education with therapist support at 3 months, from an NHS and personal social services cost perspective.

A secondary analysis of the mean incremental cost per quality-adjusted life-year (QALY) of the intervention compared with the active control from a health and social care cost perspective will also be calculated. A decision model projecting costs and QALYs into adulthood will also be developed.

#### Qualitative analysis

Interview data collected from the process evaluation will be analysed using thematic analysis.^45^ Data will be analysed using NVivo to establish themes and subthemes. A mixed-methods approach will be used to integrate quantitative and qualitative data in order to fully explore implementation of the intervention.

#### Monitoring

##### Management and oversight

There are three committees in place to oversee the trial, including a PMG, Trial Steering Committee (TSC) and Data Monitoring and Ethics Committee (DMEC). The PMG consists of all the co-investigators listed on the protocol who will meet at least every six months to discuss the study progress and overall conduct of the trial. The role of the TSC will be to provide overall supervision for the trial on behalf of the Trial Sponsor and Trial Funder, to ensure that the trial is conducted in accordance with GCP and to review the findings of the internal pilot. The DMEC is the only body involved in the trial that has access to the unblinded comparative data. The role of its members is to monitor these data and make recommendations to the TSC on whether there any ethical or safety reasons why the trial should not continue. There are no interim analyses planned; however, this does not preclude the DMEC from requesting analyses. The TSC and DMEC will meet annually. The charters are available on request from the corresponding author.

##### Adverse events

There are no anticipated serious adverse events. All adverse events will be recorded and monitored, and the chief investigator and medical expert (CH) will determine seriousness and causality and report the event to the DMEC and ethics committee where necessary. Side effects will be formally monitored via completion of the side-effects scale throughout the intervention and for 3 months after the intervention finishes (month 6).

##### Audit

The trial manager, or a nominated designee of the sponsor, shall carry out monitoring of trial data as an ongoing activity. The sponsor may decide to carry out a full audit of the trial and the trial management systems/procedures in the event of serious breaches to GCP or protocol. Trial data and evidence of monitoring and system audits will be made available for inspection by the Research Ethics Committee as required.

## Ethics and dissemination

The study received ethical and Health Research Authority (HRA) approval from North West Greater Manchester Research Ethics Committee on 23 March 2018 (ref.: 18/NW/0079: protocol V.2.0, 26 February 2018). Local approvals have been granted from the participating Trusts. The study is sponsored by Nottinghamshire Healthcare NHS Foundation Trust; neither the sponsor nor the funders will be involved in the analysis of study data or report writing. Only the UK research team will have access to the study data, which will be stored in secure locked files or password protected databases. Data will be available for inspection by the ethics committee on request. Changes to the protocol will be communicated to the ethics committee, funders, and trial registries by the trial manager (CLH). The process for obtaining participant informed consent or assent and parent/guardian informed consent will be in accordance with the ethical guidance and GCP. The investigator or their nominee and the participant or other legally authorised representative (such as the child’s parent) shall both sign and date informed consent forms before the person can participate in the study. Where the young person is 16 years and over, written consent will be required from both the parent (online supplementary file A) and young person (online supplementary file B). Where the young person is under 16 years, written parental consent will be required, alongside the young person’s assent (supplementary file C). In the event of any conflict between the parent and child, the child will not enter the study.

10.1136/bmjopen-2018-027583.supp1 Supplementary file 1

10.1136/bmjopen-2018-027583.supp2 Supplementary file 2

10.1136/bmjopen-2018-027583.supp3 Supplementary file 3

Individual participant medical information obtained as a result of this study is considered confidential and disclosure to third parties is prohibited unless warranted by an adverse event. Participant confidentiality will be further ensured by utilising identification numbers to correspond to treatment data in the computer files. No post-trial care is required; however, participants will have access to the intervention for 1 year after starting the treatment, although this access will be without therapist support after the 10/12 weeks. The chief investigator and site principal investigators declare no financial or competing interests. At the end of the trial, the data belongs to the chief investigator and the study sponsor. All members of the team will have access to the final trial data set. The findings will be published in peer-reviewed journals, presented at relevant conferences and disseminated to the public via lay summaries co-created with our PPI group. All outputs will be authored by the research team and will not involve professional writers. Access to the full protocol is available on request to the corresponding author.

## Supplementary Material

Reviewer comments

Author's manuscript
