# Supplementary material for: Investigating a therapist-guided, parent-assisted remote digital behavioural intervention for tics in children and adolescents—‘Online Remote Behavioural Intervention for Tics’ (ORBIT) trial: protocol of an internal pilot study and single-blind randomised controlled trial
Source: BMJ Open. 2019 Jan 3;9(1):e027583. doi: 10.1136/bmjopen-2018-027583 (PMC6326281; doi:10.1136/bmjopen-2018-027583)
Supplement: Supplementary file 2 [file bmjopen-2018-027583supp002.pdf]

CONSENT FORM FOR YOUNG PEOPLE 16 YEARS AND OVER

Centre Name: Nottinghamshire Healthcare NHS Foundation Trust

REC reference: 18/NW/0079

Participant Identification Number for this trial:

CONSENT FORM

Title of Project: Online Remote Behavioural Intervention for Tics (ORBIT)

Name of Researcher:

Please initial box

1. I confirm that I have read the information sheet dated _____ (version___) for the above study. I have had the opportunity to consider the information, ask questions and have had these answered satisfactorily. ☐

2. I understand that my participation is voluntary and that I am free to withdraw at any time without giving any reason, without my medical care or legal rights being affected. ☐

3. I understand that relevant sections of my medical notes and data collected during the study, may be looked at by individuals from the ORBIT team, from regulatory authorities or from the NHS Trust, where it is relevant to my taking part in this research. I give permission for these individuals to have access to my records. I understand this data will be stored in the UK (Sealed Envelopes) and Sweden (BIP and BASS) secure databases and servers. ☐

4. I understand that the information collected about me will be used to support other research in the future, and may be shared anonymously with other researchers. ☐

5. I agree to my General Practitioner being informed of my participation in the study. ☐

6. I understand that I may be asked to take part in research interviews, which will be recorded and anonymous direct quotes from these interviews may be used in study reports. ☐

7. I agree to take part in the above study. ☐

____________________	____________________	____________________
Name of Participant	Date	Signature
____________________	____________________	____________________
Name of Person taking consent	Date	Signature

When completed: 1 for participant; 1 for researcher site file; 1 (original) to be kept in medical notes.

This research was funded by the NIHR Health Technology Assessment (ref 16/19/02). The views expressed are those of the author(s) and not necessarily those of the NHS, the NIHR or the Department of Health.

Funded by NIHR
