# Supplementary material for: Investigating a therapist-guided, parent-assisted remote digital behavioural intervention for tics in children and adolescents—‘Online Remote Behavioural Intervention for Tics’ (ORBIT) trial: protocol of an internal pilot study and single-blind randomised controlled trial
Source: BMJ Open. 2019 Jan 3;9(1):e027583. doi: 10.1136/bmjopen-2018-027583 (PMC6326281; doi:10.1136/bmjopen-2018-027583)
Supplement: Supplementary file 3 [file bmjopen-2018-027583supp003.pdf]

ASSENT FORM FOR YOUNG PEOPLE UNDER 16 YEARS

Centre Name: Nottinghamshire Healthcare NHS Foundation Trust

REC reference: 18/NW/0079

Participant Identification Number for this trial:

ASSENT FORM

Title of Project: Online Remote Behavioural Intervention for Tics (ORBIT)

Name of Researcher:

Please initial box

1. I have read the information sheet dated ______ (version___) for the ORBIT study. I have discussed it with my mum/dad/carer and the researcher and I have asked questions. ☐
2. I understand that I don't have to take part and I can stop taking part any time. This is my choice and no-one will be upset with me if I stop. ☐
3. I understand that the ORBIT team may look at my medical records and the data will be kept in a database both in England and in Sweden. This will be kept safe and only the research team will see my data. ☐
4. I understand that the research team will write a report about the project. My name will not be mentioned in any reports. ☐
5. I agree to my Doctor knowing that I am taking part in the ORBIT study. ☐
6. The researcher might ask me to take part in an interview about my experiences of the ORBIT trial. I do not have to take part. If I agree to take part, the interview will be recorded but only the research team will know that I did the interview. ☐
7. I agree to take part in the ORBIT study. ☐

Name of young person	Date	Signature
Name of Person taking assent	Date	Signature

When completed: 1 for participant; 1 for researcher site file; 1 (original) to be kept in medical notes.

This research was funded by the NIHR Health Technology Assessment (ref 16/19/02). The views expressed are those of the author(s) and not necessarily those of the NHS, the NIHR or the Department of Health.
