# Supplementary material for: Investigating a therapist-guided, parent-assisted remote digital behavioural intervention for tics in children and adolescents—‘Online Remote Behavioural Intervention for Tics’ (ORBIT) trial: protocol of an internal pilot study and single-blind randomised controlled trial
Source: BMJ Open. 2019 Jan 3;9(1):e027583. doi: 10.1136/bmjopen-2018-027583 (PMC6326281; doi:10.1136/bmjopen-2018-027583)
Supplement: Author's manuscript [file bmjopen-2018-027583.draft_revisions.pdf]

BMJ Open

BMJ Open is committed to open peer review. As part of this commitment we make the peer review history of every article we publish publicly available.

When an article is published we post the peer reviewers' comments and the authors' responses online. We also post the versions of the paper that were used during peer review. These are the versions that the peer review comments apply to.

The versions of the paper that follow are the versions that were submitted during the peer review process. They are not the versions of record or the final published versions. They should not be cited or distributed as the published version of this manuscript.

BMJ Open is an open access journal and the full, final, typeset and author-corrected version of record of the manuscript is available on our site with no access controls, subscription charges or pay-per-view fees (http://bmjopen.bmj.com).

If you have any questions on BMJ Open's open peer review process please email editorial.bmjopen@bmj.com

BMJ Open

Investigating a therapist-guided, parent-assisted remote digital behavioural intervention for tics in children and adolescents – 'Online Remote Behavioural Intervention for Tics' (ORBIT) Trial: protocol of an internal pilot study and single-blind randomised controlled trial

Journal:	*BMJ Open*
Manuscript ID	bmjopen-2018-027583
Article Type:	Protocol
Date Submitted by the Author:	30-Oct-2018

Complete List of Authors:	Hall, Charlotte; Institute of Mental Health, School of Medicine, University of Nottingham<br>Davies, E. Bethan; University of Nottingham, School of Medicine<br>Andrén, Per; Karolinska Institutet Department of Clinical Neuroscience, Department of Clinical Neuroscience<br>Murphy, Tara; UCL Great Ormond Street Institute of Child Health<br>Bennett, Sophie; UCL Great Ormond Street Institute of Child Health, ; Institute of Child Health<br>Brown, Beverley; University of Nottingham, School of Medicine<br>Brown, Susan; University of Nottingham<br>Chamberlain, Liam; Queen's Medical Centre<br>Craven, Michael; Institute of Mental Health, University of Nottingham<br>Evans, Amber; UCL Great Ormond Street Institute of Child Health<br>Glazebrook, Cristine; University of Nottingham, Psychiatry<br>Heyman, Isobel; Great Ormond Street Hospital, Psychological Medicine Team, Child and Adolescent Mental Health Service<br>Hunter, Rachael; University College London, Research Dept of Primary Care and Population Health<br>Jones, Rebecca; UCL<br>Kilgariff, Joseph; Queen's Medical Centre<br>Marston, Louise; University College London,<br>Mataix-Cols, David; Karolinska Institutet, Department of Clinical Neuroscience<br>Murray, Elizabeth; University College London, Primary Care and Population Health<br>Sanderson, Charlotte; UCL Great Ormond Street Institute of Child Health<br>Serlachius , Eva ; Karolinska Institutet,<br>Hollis, Chris; University of Nottingham, Division of Psychiatry

Keywords:	behaviour therapy, exposure and response prevention, internet, Persistent (Chronic) Motor or Vocal Tic Disorder, Tourette's Disorder

For peer review only - http://bmjopen.bmj.com/site/about/guidelines.xhtml

Investigating a therapist-guided, parent-assisted remote digital behavioural intervention for tics in children and adolescents – 'Online Remote Behavioural Intervention for Tics' (ORBIT) Trial: protocol of an internal pilot study and single-blind randomised controlled trial

Authors

Charlotte L. Hall, Trial Manager, Institute of Mental Health, University of Nottingham, Nottingham, UK

E. Bethan Davies, Research Fellow and Trial Therapist, NIHR MindTech Medtech Co-operative, Institute of Mental Health, University of Nottingham, Nottingham, UK

Per Andrén, Clinical Psychologist, Department of Clinical Neuroscience, Karolinska Institutet, Stockholm, Sweden and Stockholm Health Care Services, Stockholm County Council, Sweden

Tara Murphy, Consultant Psychologist, University College London, Great Ormond Street Hospital for Children NHS Foundation Trust, Great Ormond Street, London, UK

Sophie Bennett, Senior Research Fellow, University College London, Great Ormond Street Hospital for Children NHS Foundation Trust, Great Ormond Street, London, UK

Beverley J Brown, Research Fellow, Institute of Mental Health, University of Nottingham, Nottingham, UK

Susan Brown, Research Fellow, NIHR MindTech Medtech Co-operative, Institute of Mental Health, University of Nottingham, Nottingham, UK

Liam Chamberlain, Trial Therapist, Division of Psychiatry and Applied Psychology, University of Nottingham, Queen's Medical Centre, Nottingham, UK

Michael P. Craven, Senior Research Fellow, NIHR MindTech Medtech Co-operative, Institute of Mental Health, University of Nottingham, Nottingham, UK

Amber Evans, Trial Therapist, University College London, Great Ormond Street Hospital for Children NHS Foundation Trust, Great Ormond Street, London, UK

Cris Glazebrook, Professor of Health Psychology, Division of Psychiatry and Applied Psychology, Institute of Mental Health, University of Nottingham, Nottingham, UK

Isobel Heyman, Consultant Psychiatrist, Great Ormond Street Hospital for Children NHS Foundation Trust, Great Ormond Street, London, UK

Rachael Hunter, Health Economist, Research Department of Primary care and Population health and Priment CTU, University College London, Royal Free Campus, London, UK

For peer review only - http://bmjopen.bmj.com/site/about/guidelines.xhtml

Rebecca Jones, Senior Research Associate, Research Department of Primary care and Population health and Priment CTU, University College London, Royal Free Campus, London, UK

Joseph Kilgariff, Advanced Nurse, Division of Psychiatry and Applied Psychology, University of Nottingham, Queen's Medical Centre, Nottingham, UK

Louise Marston, Principal Research Associate, Research Department of Primary care and Population Health and Priment CTU, University College London, Royal Free Campus, London, UK

David Mataix-Cols, Professor of Child and Adolescent Psychiatric Science, Department of Clinical Neuroscience, Karolinska Institutet, Stockholm, Sweden and Stockholm Health Care Services, Stockholm County Council, Sweden

Elizabeth Murray, Professor of eHealth and Primary Care, eHealth Unit, Research Department of Primary Care and Population Health, University College London, Royal Free Campus, London, UK

Charlotte Sanderson, Research Clinical Psychologist, University College London, Great Ormond Street Hospital for Children NHS Foundation Trust, Great Ormond Street, London, UK

Eva Serlachius, Assistant Professor in Child and Adolescent Psychiatry, Department of Clinical Neuroscience, Karolinska Institutet, Stockholm, Sweden and Stockholm Health Care Services, Stockholm County Council, Sweden

Chris Hollis, Professor of Child & Adolescent Psychiatry, Developmental Psychiatry, University of Nottingham, Queens Medical Centre, Nottingham, UK

On behalf of the ORBIT Trial team

Corresponding Author
Chris Hollis (*PhD, FRCPsych)*
Institute of Mental Health, University of Nottingham, Triumph Road, Nottingham, NG7 2TU.
Email: chris.hollis@nottingham.ac.uk Tel: 0115 823 0258

Key words: behaviour therapy; exposure and response prevention; Internet; Persistent (Chronic) Motor or Vocal Tic Disorder; Tourette's Disorder

Word count: 4,254

For peer review only - http://bmjopen.bmj.com/site/about/guidelines.xhtml

ABSTRACT

Introduction

Tourette syndrome and chronic tic disorder are common, disabling childhood-onset conditions. Guidelines recommend that behavioural therapy should be offered as first-line treatment for children with tics. However, there are very few trained behaviour therapists for tics and many patients cannot access appropriate care. This trial investigates whether an internet-delivered intervention for tics can reduce severity of symptoms.

Method and analysis

This parallel-group, single-blind, randomised controlled superiority trial with an internal pilot will recruit children and young people (aged 9-17 years) with tic disorders. Participants will be randomised to receive 10-weeks of either online, remotely-delivered, therapist-supported exposure response prevention (ERP) behavioural therapy for tics, or online, remotely-delivered, therapist-supported education about tics and co-occurring conditions. Participants will be followed-up mid-treatment, and 3-, 6-, 12-, and 18-month post-randomisation.

The primary outcome is reduction in tic severity as measured on the Yale Global Tic Severity Scale (YGTSS) total tic severity score. Secondary outcomes include a cost-effectiveness analysis and estimate of the longer-term impact on patient outcomes and healthcare services. An integrated process evaluation will analyse quantitative and qualitative data in order to fully explore the implementation of the intervention and identify barriers and facilitators to implementation. The trial is funded by the National Institute of Health Research (NIHR), Health Technology Assessment (16/19/02).

Ethics and dissemination

For peer review only - http://bmjopen.bmj.com/site/about/guidelines.xhtml

The findings from the study will inform clinicians, healthcare providers and policy makers about the clinical and cost-effectiveness of an internet delivered treatment for children and young people with tics. The results will be submitted for publication in peer-reviewed journals. The study has received ethical approval from North West Greater Manchester Research Ethics Committee (Ref: 18/NW/0079).

Trial registration: ISRCTN70758207 and ClinicalTrials.gov (NCT03483493).

For peer review only

STRENGTHS AND LIMITATIONS OF THIS STUDY

- This study is a 10-week, parallel-group, single blind, non-commercial, randomised controlled superiority trial with an internal pilot; the first large trial for internet delivered therapy for tics in the UK.
- The study design and methodology are based upon an initial pilot study of internet-delivered ERP for tics conducted in Sweden.
- The protocol was created with a multidisciplinary team of experts including, healthcare professionals, patient and public involvement members, and health service researchers.
- This trial is only available to English-speaking participants with access to a computer/internet.

For peer review only - http://bmjopen.bmj.com/site/about/guidelines.xhtml

INTRODUCTION

Tourette syndrome (TS) and chronic tic disorder (CTD) are common, disabling, childhood-onset conditions affecting up to 1% of young people and are associated with significant distress, psychosocial impairment and reduced quality of life. [1] The majority of patients additionally experience comorbidities such as attention deficit hyperactivity disorder (ADHD), obsessive-compulsive disorder (OCD), depression, anxiety, and self-injurious behaviour. [2] Evidence-based interventions for the treatment of tics in children and adolescents with TS include pharmacological treatment and behaviour therapy. [1 3-5] European clinical guidelines[3] and a National Institute of Health Research (NIHR) Health Technology Assessment (HTA) Evidence Synthesis[1] recommend that behaviour therapy should be offered as a first-line intervention for tics in children and adolescents in a stepped-care approach.

Despite these recommendations, only 1 in 5 young people with TS are currently able to access behaviour therapy for tics, while around 50% receive medication,[6] which is associated with a significant risk of adverse effects including weight gain and sedation.[4 5] Furthermore, those young people who manage to access behaviour therapy typically receive four or fewer face-to-face therapy sessions,[6] less than half the number recommended.[7] Commonly used behaviour therapies include: *habit reversal training (HRT),* whereby patients are taught to implement and maintain an action referred to as a 'competing response' when a premonitory urge to tic is experienced (the urge to tic often felt before the tic is expressed); *comprehensive behavioural intervention for tics (CBIT),* which includes HRT and adds additional elements of relaxation training, functional analyses (identification of situations which could exacerbate tics) and social support; and *exposure and response prevention (ERP),* where patients tolerate premonitory urge sensations and learn from their therapist how to resist their tics.

For peer review only - http://bmjopen.bmj.com/site/about/guidelines.xhtml

The effectiveness of behavioural therapy for reducing tics has been well established,[1] with systematic reviews demonstrating a similar magnitude of effect for HRT/CBIT compared to pharmaceutical interventions.[1, 4] A recent trial compared 10 weeks of behavioural therapy (either HRT or ERP) with education and pharmacological treatment and found the behavioural therapy to be as effective as pharmacological treatment for reducing tic severity, with both being superior to education.[8] Additionally, although there is a lack of evidence from randomised controlled trials (RCTs) for ERP, the evidence from available studies suggests both HRT and ERP may be at least equally effective in reducing tics, with some evidence showing a tendency to favour ERP.[3] The effectiveness of these behavioural interventions in conjunction with the lack of side effects compared to pharmacotherapy supports the use of behaviour therapy as a first-line intervention.. However, there is a lack of trained therapists across many European countries, leaving many patients unable to access evidence-based behaviour therapy. This need for improved access to therapy was highlighted in a recent study whereby families of children with TS noted the struggle to access limited behavioural therapy resources, despite 76% of parents saying they would want behavioural therapy to be available for their child.[6]

Over the last decade, internet-delivered cognitive behaviour therapy (ICBT) has developed, enabling the delivery of effective, but less therapist intensive, cognitive and behavioural interventions remotely, which can increase the availability of evidence based treatments. Across diagnostic conditions, studies have not only shown efficacy of ICBT compared to no-treatment control conditions, but meta analyses comparing face-to-face delivered CBT with ICBT have demonstrated comparable results in terms of symptom reduction for adults with mental health problems,[9 ,10] which could also result in 50% cost savings.[10] However, therapist guidance during

For peer review only - http://bmjopen.bmj.com/site/about/guidelines.xhtml

ICBT has been shown to be an important contributing factor in determining outcome.[11] Although self-guided ICBT is superficially attractive, given the very low costs of implementation, research evidence demonstrates very low adherence rates.[12, 13]

Recently, researchers at the Karolinska Institutet, Sweden, have developed a technical platform for ICBT called BIP (*Barninternetprojektet,* Swedish for *Child Internet Project*; http://www.bup.se/bip). The platform has been used to deliver therapist-supported ICBT for a range of different conditions, including phobia,[14] anxiety,[15] and obsessive-compulsive disorder.[16] The findings support the use of therapist guided ICBT using the BIP system, however, there is little research evidence with regards to the effectiveness of ICBT and TS. In a recent systematic review of digital health interventions, Hollis and colleagues[17] found the majority of interventions have been designed to help children and young people at risk for developing, or with a diagnosis of, an anxiety disorder and/or depression, with areas such as TS being largely overlooked. Innovations in behavioural therapies for tics include delivery of CBIT treatment remotely via video over the internet with a therapist. Two pilot RCTs have compared CBIT delivered remotely via videoconference to traditional face-to-face delivery in children with TS. Results showed significant tic reduction for both groups, with no difference between the modes of delivery.[18, 19] The method of delivering CBIT was rated as highly acceptable by the participants.[18]

One Swedish pilot study, upon which this current trial is based, has used the BIP platform to deliver ICBT with remote therapist support. The study compared tic reduction in children aged 8-16 years randomised to receive either ERP or HRT delivered online via the BIP platform.[20] Participants in both groups showed improvement after treatment completion, but only participants in the ERP arm showed significant reduction on the primary outcome measure Yale

For peer review only - http://bmjopen.bmj.com/site/about/guidelines.xhtml

Global Tic Severity Scale (YGTSS) total tic severity score. . The gains were maintained for at least 12 months after the end of treatment. Although this study was not designed or powered as a definitive efficacy trial, these findings show that ERP treatment delivered online via BIP is acceptable to participants and shows promise in reducing tics. Furthermore, the study reported no severe adverse events. There were no drop-outs and no data loss for the primary outcome, and 83% of parents and children rated the treatment as good or very good, indicating the trial was highly acceptable to patients in Sweden.

However, the evidence available for the BIP treatment programs has all been gathered in Sweden and we have limited experience of delivering therapist supported online ERP to children and adolescents in the UK. There is evidence that uptake and use of digital health interventions is highly context dependent[21, 22] and it would therefore be unwise to assume that a delivery package that works in Sweden will work equally well in the UK.

The overarching aim of the Online Remote Behavioural Intervention for Tics (ORBIT) trial is to evaluate the clinical and cost-effectiveness of BIP TIC, a therapist-guided, parent-assisted ERP programme for tics in young people with TS in the UK.

Specific objectives are to:

1. To optimise the design and delivery of BIP TIC in partnership with young people with TS, carers and healthcare professionals (HCPs) to maximise acceptability, effectiveness and long-term uptake.
2. To undertake an internal pilot to assess whether recruitment, engagement with the intervention and retention to the trial outcomes are sufficient to allow the trial to progress and provide a definitive answer on effectiveness.

For peer review only - http://bmjopen.bmj.com/site/about/guidelines.xhtml

3. To determine the clinical effectiveness of BIP TIC compared to online tic-related education in reducing tics, as measured by the masked assessor-rated Yale Global Tic Severity Scale (YGTSS) total tic severity score (primary outcome).
4. To evaluate the cost-effectiveness of BIP TIC.
5. To estimate the longer-term impact on patient outcomes and health service costs.
6. To conduct a process analysis in line with MRC guidelines for complex interventions to determine barriers and facilitators to implementation.

The primary hypothesis is that the BIP TIC therapist-guided remote digital ERP intervention will be superior to a comparator intervention of therapist-guided online tic-related education in reducing tics, as measured by the masked assessor-rated YGTSS total tic severity score[23].

METHODS AND ANALYSIS

Trial design

The design is a 10-week, parallel-group, single-blind, non-commercial, randomised controlled superiority trial with an internal pilot. Participants will be randomised to receive 10-weeks of online, remotely-delivered, therapist-supported ERP for tics, or online, remotely-delivered, therapist-supported education for tics. Participants will be followed-up mid-treatment, and at 3-, 6-, 12-, and 18-months post-randomisation. Up until the 6-month follow-up participants are encouraged not to change medication or start alternative therapies for tics. Months 12 and 18 are naturalistic follow-up points where participants are free to access any treatments in accordance with standard practice recommended by their usual treating clinician. A sub-sample of participants and parents will be purposively selected to participate in process evaluation

For peer review only - http://bmjopen.bmj.com/site/about/guidelines.xhtml

interviews after the 3-month follow-up. Interviews will also be conducted with therapists, clinicians recruiting to the study and commissioners. The process evaluation will also link data on usage of the intervention with outcomes. A schematic diagram of the study design is shown in Figure 1 and a more detailed participant flow is shown in Figure 2.

An internal pilot will be conducted until the end of the 9th month to determine whether recruitment, engagement with the intervention and retention to the trial are sufficient to allow the trial to progress, the stop/go rules for proceeding are:

1) The study needs to have recruited 30% of the sample (66 participants).
2) At least 60% of participants need to have completed the intervention (defined as the child/adolescent completing at least their first four chapters).
3) 80% of participants who have reached the relevant time window need to have completed the primary outcome measure.

The trial was prospectively registered with ISRTCN (ISRCTN70758207) and ClinicalTrials.gov (NCT03483493).

<<INSERT FIGURE 1>>>

Setting

The intervention will be remotely delivered from two national centres for treating tics in the U.K. (Queen's Medical Centre, Nottingham and Great Ormond Street Hospital, London), serving geographically and demographically diverse populations. However, multiple Patient Identification Centres (PICs) will be involved in recruiting participants into the two research sites.

For peer review only - http://bmjopen.bmj.com/site/about/guidelines.xhtml

Recruitment and eligibility

Participant identification

Participants will be identified and recruited by either: (1) clinic staff or clinical study officers (CSOs) at Patient Identification Centres (PICs) across National Health Services (NHS) Trusts in England; or (2) the two study sites (Nottingham and London) or (3) via Tourettes Action (tic disorder charity) website, our study website, or social media, based on the following criteria:

Inclusion criteria

1) Aged 9 to 17 years-old.
2) Suspected or confirmed Tourette syndrome or chronic tic disorder.
   - Including moderate/ severe tics: Total Tic Severity Score (TTTS) >15 on the YGTSS; TTSS score>10 if motor or vocal tics only.
3) Competent to provide written, informed consent (parental consent for child aged <16 years).
4) Broadband internet access and regular PC/laptop/Mac user, with mobile phone SMS.

Exclusion criteria

1) Receipt of/engaged in structured behavioural intervention for tics (e.g. HRT/CBIT or ERP) within the last 12 months.
2) Change to medication for tics (start or stop) within the previous 2 months.
3) Diagnoses of alcohol/substance dependence, psychosis, suicidality, or anorexia nervosa.
4) Moderate/severe intellectual disability.
5) Immediate risk to self or others.
6) Parent or child not able to speak or read/write English.

For peer review only

For peer review only - http://bmjopen.bmj.com/site/about/guidelines.xhtml

Patients will be provided with a brief information sheet and a 'consent to contact' (C2C) form: for NHS referred patients, these forms will be given by a member of the usual care team (or CSO); for self-recruited patients, these will be completed online.

Initial screening

Upon receipt of a C2C, a member of the research team will undertake a telephone screening interview with the potential participant to determine basic eligibility. Potential participants who meet the initial eligibility requirements will be invited to attend a face-to-face screening/baseline appointment held at one of the two study sites. All participants will be reimbursed for their travel costs to attend this appointment.

Before participants attend the face-to-face screening/baseline appointment parents will be asked to complete an online parent and teacher Development and Wellbeing Assessment (DAWBA)[24] (see secondary outcomes for further details). Completion of the parent DAWBA is a requirement for enrolment into the trial; findings from the DAWBA will be used to exclude people who score highly on self-harm, psychosis, anorexia nervosa or suicidality. It is the parent's decision to involve the teacher in completing the teacher DAWBA.

Face-to-face screening appointment

Potential participants found eligible from the initial telephone screening will be invited to the face-to-face screening/baseline appointment. Consent will be taken at this appointment by a researcher/assessor trained in Good Clinical Practice (GCP), further details about which are listed under 'ethics and dissemination'. Participants will be provided with information sheets which have been developed with our patient and public involvement (PPI) group. Families will be encouraged to ask the research team any questions. Where the participant is a child (under 16-years-old) an age appropriate participant information sheet will be provided.

For peer review only - http://bmjopen.bmj.com/site/about/guidelines.xhtml

A trained assessor will conduct the YGTSS[23] assessment to determine the presence of tics (inclusion criteria) and the Child and Adolescent Intellectual Disability Screening Questionnaire *(*CAIDS-Q)[25] to screen for intellectual disability (exclusion criteria). Details of the YGTSS are described under the primary outcome.

Patients who are not eligible because they have recently changed their tic medication (within 2-months), or received a behavioural therapy for tics within the specified exclusion time frame (12 months), may be eligible for re-screening at a later date when these time exclusions have passed. Patients who meet the eligibility criteria, after completion of all screening assessments, will be asked to complete baseline measures and will be introduced to the BIP system.

Interventions

The BIP treatment programs are delivered via a secure internet platform that enables presentation of different treatment content to different patient populations. Patients log in via personal user names and passwords to access the treatment content which usually includes text, pictures, instructional videos and worksheets (see link for video of the intervention https://vimeo.com/294763841/628b99ddfb). The treatment content for both groups is presented in chapters, like a self-help book.

Both the ERP (intervention) and the education (active control) are delivered via the BIP platform. The interventions consist of 10 separate child-and parent/carer-chapters which are designed to be delivered over 10 weeks, with access to therapist support during this time.

The child/young person and their parent/carer are provided with their own separate login to the BIP platform to access the relevant child or parent/carer chapters. The first four chapters cover

For peer review only - http://bmjopen.bmj.com/site/about/guidelines.xhtml

the main content of the treatment; completion of these four child chapters is a minimum requirement to meet treatment completion criteria.

Both children and parents/carers have regular contact with a therapist during the 10 weeks via messages that can be sent inside the treatment platform (resembling an email) or telephone if needed. The therapist can also directly comment on exercises that the participant has been working on, and give specific feedback to motivate the patient. The participant typically has contact with the therapist several times a week, although the contact is asynchronous. The therapist is available to support the participants during these 10-weeks, although support may be delivered over a 12-week period if necessary to allow for breaks during holiday and festive periods. Access to the BIP treatment program is granted for 1-year after the 10 weeks of therapist-guided treatment, with the remaining access to the BIP platform being unsupported. The therapist manuals for both interventions were written by the co-applicants.

Therapist guided, internet-delivered ERP Intervention (BIP TIC; experimental group)

The treatment consists of evidence-based interventions adapted from previously published treatment manuals on ERP and established behavioural intervention for tics protocols.[26, 27] During the treatment, participants are instructed to practice suppressing their tics: this is known as ‘response prevention’. Then, with the help of their parent/carer, the participant is instructed to provoke premonitory urges and control all of their tics: this is known as ‘exposure’. Through treatment, the child gains mastery in tolerating the urge, controlling the tics, and is able to do so for increasingly longer periods of time. The participants also receive detailed education about tics and strategies to promote tic management at school.

For peer review only

For peer review only - http://bmjopen.bmj.com/site/about/guidelines.xhtml

The parent modules contain information regarding parent coping strategies, how to support their child in working with BIP and functional analysis relating to tics.

Therapist guided, internet-delivered education (active control group)

An active comparator intervention was chosen to ensure that all participants still received an internet-delivered therapist-guided intervention over-and-above what would typically be received in standard care. Education about tics (without a specific focus on training behavioural strategies to reduce tics) was chosen to enable a comparison to previous trials of face-to-face behaviour therapy for tics.[28]

The comparator intervention was developed for the trial and consisted of educational information about TS and co-occurring conditions. The material was adapted and updated from the educational intervention used in two previous randomised controlled trials.[26, 28]

The chapters have been matched in length to that of the behavioural intervention. The comparator intervention reviews the definition of tics, natural history, common presentations, prevalence, aetiology, risks and protective factors and strategies for describing tics to other people etc. Problem-solving and development of expertise in tic disorders is emphasised. The parent modules emphasise parent coping strategies and self-care in caring for a child with tic disorder. There is no information on tic control within the comparator intervention.

Start of treatment

The participant will agree a start date for commencing therapy with their assessor at the screening/baseline appointment (typically within 24-48 hours of the baseline appointment). The participant will be reminded that it is important to log-on and start the therapy on the allocated date. At the start, the therapist will release the first two chapters of the intervention for the

For peer review only - http://bmjopen.bmj.com/site/about/guidelines.xhtml

participants to complete and monitor their progress, sending reminders and feedback where necessary. If the participant does not start the intervention at the agreed start time, the 10-week start date does not alter.

All therapists will undergo training delivered by a supervisor (TM or JK) before commencing either intervention. Therapists will be closely supervised by TM or JK to ensure adherence to protocol. The team from the Karolinska Institutet will provide technical support throughout the trial.

Measures and outcomes

Primary outcome

The primary outcome is the severity of tics as measured by the TTSS (0-50) on the YGTSS .[23] The primary end point is 3-months post-randomisation. The TTSS combines separate scales for motor tics (0-25) and for vocal tics (0-25). The primary outcome measure (TTSS) will be measured at baseline (pre-intervention), 3-months, 6, 12 and 18-months post randomisation.

The YGTSS is administered by a blinded assessor as an investigator-based semi-structured interview focussing on motor and vocal tic frequency, severity and tic related impairment over the previous week. The baseline assessment will be conducted face-to-face at the baseline/screening appointment. When possible, the follow-up assessments will be conducted by the same blinded assessor that conducted the baseline assessment, via video-conference call/WebEx or telephone. With the participants' permission, all YGTSS assessments will be recorded for quality checks.

All assessors/researchers will be trained in conducting YGTSS by a clinical expert (TM). Agreement with the expert rater will be established at the start of the trial and monitored every six-months. Assessors have to be within 15% of the expert rater (TM) for the Total Motor Tic

For peer review only - http://bmjopen.bmj.com/site/about/guidelines.xhtml

score, the Total Vocal Tic score and the Total Tic Score on 3 YGTSS recordings at baseline and 4 randomly selected recordings at follow-up. Assessors who do not meet this threshold of agreement will be re-trained.

Secondary outcomes

A flowchart of study assessments is shown in Table 1 which documents the time point for completion and the informant. See also Figure 2.

Table 1. Schedule of assessments

For peer review only

For peer review only

**Months post-randomisation**	**0**	**0**	**1**	**1**	**3**	**6**	**12**	**18**	**Completed by**
**Time Point**	Telephone screen	Baseline assessment	Mid-treatment (3 weeks)	Mid-treatment (5 weeks)	Primary end point	6-months	12-months	18-months	
Screening for eligibility	X	X							R
SDQ & DAWBA (conducted post telephone screen & prior to baseline)	X								P & T
YGTSS TTSS		X			X	X	X	X	R
YGTSS Impairment		X			X	X	X	X	R
CAIDS-Q		X							P/R
SCQ		X							P
SNAP-IV		X							P
Demographics		X							R
PUTS		X							C
PTQ		X		X	X	X	X	X	P
CGAS		X			X	X	X	X	R
CHU9D		X			X	X	X	X	P
Modified CSRI (inc school attendance)		X			X	X	X	X	P/R
MFQ-(Self-report)		X		X	X	X	X	X	C
SCAS (Self-report)		X			X	X	X	X	C
C&A GTS-QOL		X			X	X	X	X	C
Adverse effects/side effects		X		X	X	X			P/C
Concomitant interventions		X			X	X	X	X	P/R
SDQ (P)					X	X	X	X	P

CGI-I					X	X	X	X	R
Treatment credibility			X						P/C
Treatment satisfaction					X				P/C
Need for further treatment					X				P/C
Interview (Process evaluation)					X				

C = child; C&A-GTS-QOL = Child and adolescent version of the Gilles de la Tourette Syndrome Quality of Life Scale; CAIDS-Q = Child and Adolescent Intellectual Disability Screening Questionnaire; CGAS = Children's Global Assessment Scale; CGI-I = Clinical Global Impressions- Improvement; CHU9D = Child Health Utility 9D; CSRI = Client Service Receipt Inventory; DAWBA = Development and Wellbeing Assessment; MFQ = Mood and Feelings Questionnaire; P = parent; PTQ = Parent Tic Questionnaire; PUTS = Premonitory Urges for Tics Scale; R = researcher; SCAS = Spence Child Anxiety Scale; SCQ = Social Communication Questionnaire; SDQ = Strengths and Difficulties Questionnaire; SNAP-IV; Swanson, Nolan and Pelham Rating Scale; T = teacher; TTSS= Total Tic Severity Scale; YGTSS = Yale Global Tic Severity Scale.

<<INSERT FIGURE 2>>>

- *YGTSS – Impairment scale*:[23] The impairment scale (0-50) forms part of the YGTSS described above. The rating focuses on distress and impairment experienced in interpersonal, academic, and occupational domains.
- *Parent Tic Questionnaire (PTQ)*:[29] The PTQ assesses the number, frequency, and intensity of motor and vocal tics.
- *Clinical Global Impressions Scale (CGI)-Improvement*:[30] The CGI Improvement provides an overall clinician-determined summary measure of symptom improvement.

For peer review only - http://bmjopen.bmj.com/site/about/guidelines.xhtml

- *Children's Global Assessment Scale (CGAS)*:[31] The CGAS is a 0-100 scale that integrates psychological, social and academic functioning in children as a measure of psychiatric disturbance.
- *Strengths and Difficulties Questionnaire (SDQ – parent completed)*:[32] The SDQ is a brief measure of behavioural and emotional difficulties. The SDQ will be completed by parents as part of DAWBA at baseline and at follow-up via an online platform.
- *The Mood and Feelings Questionnaire (MFQ; Child completed version)*: [33] The MFQ reports depressive symptoms as specified by the DSM-III-R diagnostic criteria for major depression.
- *Spence Child Anxiety Scale (SCAS* - *self-report)*:[34] The SCAS is a self-report measure that evaluates symptoms relating to separation anxiety, social phobia, obsessive-compulsive disorder, pain, agoraphobia, generalised anxiety and fears of physical injury.
- *Child Health Utility 9D (CHU9D; parent and child completed versions)*: [35] The CHU9D is a paediatric quality of life measure for use in health care resource-allocation decision making.
- *The Child and Adolescent Gilles de la Tourette Syndrome–Quality of Life Scale (C&A-GTS-QOL)*:[36] The C&A-GTS-QOL is a disease-specific measure of health-related quality of life designed for children and adolescents with TS.
- *Client Service Receipt Interview (CSRI)*:[37] The CSRI is a flexible research instrument developed to collect information on service receipt, service-related issues and income. A modified version of the CSRI will be completed which also combines elements of the Child and Adolescent Service Use Schedule (CA-SUS).[38]

For peer review only - http://bmjopen.bmj.com/site/about/guidelines.xhtml

- *Adverse events (Side effects)*: Adverse events/side effects will be recorded on a modified version of the side effects scale developed by Hill and Taylor.[39]

The above mentioned scales all have established reliability, validity and history of use in clinical and research settings. Some further measures were developed by the research team for the purpose of this study, these include:

- *Treatment credibility:* The questionnaire consists of 2-items which ask how suitable the child and parent believe the internet treatment is for managing tics, and how much improvement they expect from the treatment.
- *Treatment satisfaction and need for further treatment:* 7-items ask about the child's/parent's satisfaction with the treatment and an additional question asks whether they feel more tic treatment is required.
- *Concomitant interventions:* To assess what other treatments/interventions the child/young person is accessing during the study period, parents will be asked to complete a short questionnaire documenting other interventions (including medication) in progress.

Additional baseline measures

To further characterise the sample at baseline, the following measures will also be recorded:

- *Demographics questionnaire*: Records the child's age, gender, ethnicity, parental education/ occupation, list of the child's current diagnoses and interventions (including medications) and general practitioner (GP) and school details.

For peer review only - http://bmjopen.bmj.com/site/about/guidelines.xhtml

- *Social Communication Questionnaire (SCQ)*:[40] The SCQ is a screening tool for Autism Spectrum Disorder (ASD).
- *Premonitory Urge for Tics Scale (PUTS)*:[41] The PUTS is a self-report instrument that measures premonitory urges in tic disorders.
- *Swanson, Nolan, and Pelham Rating Scale (SNAP-IV)*:[42] The SNAP-IV is a behavioural rating scale that includes the core symptoms of ADHD and oppositional defiant disorder (ODD).

Process evaluation

The process evaluation will follow the Medical Research Council (MRC) guidelines for evaluating the implementation of complex interventions[43 44]. It will explore the fidelity and implementation of intervention, and make recommendations for adaptations. It will also examine the potential mechanisms underlying participant behaviour change and probe for unexpected consequences.

Interviews will be conducted with therapists and their supervisors supporting the intervention (target n>5), clinicians recruiting to the study (n = >10), children (n=>20 in each arm) and parents of children in both arms (n=>20 in each arm). Online feedback from participants will also be analysed together with indices recorded as part of the BIP system such as total therapist time, number of chapters clicked through and number of patient log-ins.

Patient and public involvement (PPI)

A group of PPI members was created to shape and guide the study. The group is comprised of four parents and four children and young people with a diagnosis of Tourette syndrome, plus two

For peer review only - http://bmjopen.bmj.com/site/about/guidelines.xhtml

PPI members with previous experience of large studies. The design of the trial and its dissemination will also have been shaped by PPI in the following ways:

- reviewing and selection of outcome measures
- co-development of study materials and recruitment processes
- reviewing the interventions and providing feedback on all chapters
- attending ongoing trial team meetings to provide PPI perspective and assist with troubleshooting (e.g. recruitment and retention)
- guiding interview topics and shaping questions for process evaluation,
- co-creation of lay summaries and other dissemination materials

The PPI group mainly contributes remotely, in order to enable involvement from members who are not geographically close. Face-to-face input occurs at key points, for example attendance at trial team meetings.

Sample size

To detect a clinically important average difference of 0.5 of a standard deviation between intervention and comparator with 90% power at $p<0.05$ (2-sided), after allowing for 20% dropout, requires a total sample size of 220 participants. A systematic review found the average estimate for the standard deviation (SD) of the YGTSS (TTSS) from 19 trials of behavioural intervention for tics was 6.6.[17] Thus, the trial is powered to detect an average difference of 3.3 on the YGTSS.

Recruitment is planned to be conducted within an 18-month period. We are not aware of any other competing studies that are in progress or planned that would affect recruitment. Input received from young people and carers in developing this trial indicates that participation in the trial will

For peer review only - http://bmjopen.bmj.com/site/about/guidelines.xhtml

For peer review only

be attractive as it guarantees access to an intervention. Our national recruitment network of specialist (PIC) clinics, including multiple community child and adolescent mental health services (CAMHS) and paediatric clinics and the charity Tourettes Action, have the capacity to meet the recruitment target within the time window. Recruitment figures will be monitored monthly by the Trial Manager (CLH) and any shortfall will be reported to the project management group (PMG).

Randomisation

Randomisation will be conducted using the Sealed Envelope online randomisation system and managed by Priment Clinical Trials Unit (CTU). Participants will be randomised online by the researcher/assessor. Randomisations will be distributed equally between the two arms (ratio 1:1) and stratified by study site using block randomisation with varying block sizes. The researcher will remain blind to the treatment allocation, and the therapist will be notified of the treatment allocation. Therapists will set up participants' treatment on the BIP system.

All participants receive the same outcome measures so assessors will not know which group the participant is in by the measurements. If an assessor becomes unblinded, subsequent assessments for that participant will be conducted by a different assessor (blind to arm allocation) where possible. In case of a medical emergency, participants will be able to disclose to the treating physician (e.g. GP) what treatment they received without unblinding the researchers. Outcome assessors will guess the treatment allocation after each assessment, to be able to calculate if the guesses are better than random.

Data management and analysis

Participants who do not complete therapy or measures at a given time point will still be followed up until the end of the trial unless they inform the research team they would like to withdraw from

For peer review only - http://bmjopen.bmj.com/site/about/guidelines.xhtml

For peer review only

the trial. Participants will be reimbursed with £20 vouchers for completing measures at baseline and each follow-up time point, except the mid-treatment follow-ups (3 and 5 weeks).

All aspects of data management of the study will comply with the UK Data Protection Act 1998/ General Data Protection Regulation (GDPR), Priment's standard operating procedures (SOPs) and GCP. Outcome measures will be stored in BASS, a rating platform stored on a Swedish server. Participant completed outcome measures are directly entered into an online database by the participant. Assessor/researcher completed measures may initially be entered on a paper case report form (CRF) or directly into the database. Data extraction will be performed within the UK.

Statistical analysis

All baseline variables will be summarised by randomised group. Categorical data will be reported as frequencies (%) and continuous data will be reported as mean (SD) unless skewed then they will be reported as median (interquartile range). No statistical tests will be carried out comparing baseline variables. The primary outcome (total tic severity score [TTSS] of the YGTSS at 3-months post-randomisation) will be analysed using a linear regression model, including baseline TTSS and centre (the stratification variable). If the assumptions of linear regression are violated, another suitable method will be used. Primary analyses will be performed using intention to treat principles. Predictors of missingness will be examined. If any are found, they will be included in statistical models in a supportive analysis. There are no planned subgroup analyses.

Secondary outcomes will be analysed using similar statistical methods as the primary outcome. Continuous outcomes will be analysed with linear regression. Dichotomous outcomes will be analysed using logistic regression. Results will be presented as estimated differences or odds ratios as appropriate, with accompanying 95% CI. P values will not be reported for secondary outcomes.

For peer review only - http://bmjopen.bmj.com/site/about/guidelines.xhtml

Economic analysis

The primary cost-effectiveness analysis for the within-trial evaluation will be the mean incremental cost per point change in YGTSS of treatment as usual (TAU) plus BIP TIC compared to TAU plus online education with therapist support at 3 months, from an NHS and personal social services cost perspective.

A secondary analysis of the mean incremental cost per quality-adjusted life-year (QALY) of the intervention compared to the active control from a health and social care cost perspective will also be calculated. A decision model projecting costs and QALYs into adulthood will also be developed.

Qualitative analysis

Interview data collected from the process evaluation will be analysed using thematic analysis.[45] Data will be analysed using NVivo to establish themes and subthemes. A mixed-methods approach will be used to integrate quantitative and qualitative data in order to fully explore implementation of the intervention.

Monitoring

Management and oversight

There are three committees in place to oversee the trial, including: a Project Management Group (PMG), Trial Steering Committee (TSC) and Data Monitoring and Ethics Committee (DMEC). The PMG consists of all the co-investigators listed on the protocol who will meet at least every six months to discuss the study progress and overall conduct of the trial. The role of the TSC will be to provide overall supervision for the trial on behalf of the Trial Sponsor and Trial Funder, to

For peer review only - http://bmjopen.bmj.com/site/about/guidelines.xhtml

ensure that the trial is conducted in accordance with GCP, and to review the findings of the internal pilot. The DMEC is the only body involved in the trial that has access to the unblinded comparative data. The role of its members is to monitor these data and make recommendations to the TSC on whether there any ethical or safety reasons why the trial should not continue. There are no interim analyses planned, however this does not preclude the DMEC from requesting analyses. The TSC and DMEC will meet annually. The charters are available on request from the corresponding author.

Adverse events

There are no anticipated serious adverse events. All adverse events will be recorded and monitored and the Chief Investigator and medical expert (CH) will determine seriousness and causality and report the event to the DMEC and ethics committee where necessary. Side effects will be formally monitored via completion of the side-effects scale throughout the intervention and for 3-months after the intervention finishes (month 6).

Audit

The Trial Manager, or a nominated designee of the Sponsor, shall carry out monitoring of trial data as an ongoing activity. The Sponsor may decide to carry out a full audit of the trial and the trial management systems/ procedures in the event of serious breaches to GCP or protocol. Trial data and evidence of monitoring and system audits will be made available for inspection by the Research Ethics Committee (REC) as required.

ETHICS AND DISSEMINATION

For peer review only - http://bmjopen.bmj.com/site/about/guidelines.xhtml

The study received ethical and Health Research Authority (HRA) approval from North West Greater Manchester Research Ethics Committee on 23rd March 2018 (Ref: 18/NW/0079: protocol version 2.0, 26th February 2018). Local approvals have been granted from the participating Trusts. The study is sponsored by Nottinghamshire Healthcare NHS Foundation Trust; neither the sponsor nor the funders will be involved in the analysis of study data or report writing. Only the UK research team will have access to the study data, which will be stored in secure locked files or password protected databases. Data will be available for inspection by the ethics committee upon request. Changes to the protocol will be communicated to the ethics committee, funders, and trial registries by the trial manager (CLH). The process for obtaining participant informed consent or assent and parent/ guardian informed consent will be in accordance with the ethical guidance and GCP. The investigator or their nominee and the participant or other legally authorised representative (such as the child's parent) shall both sign and date informed consent forms before the person can participate in the study. Where the young person is 16-years and over, written consent will be required from both the parent (Supplementary File A) and young person (Supplementary File B). Where the young person is under 16-years, written parental consent will be required, alongside the young person's assent (Supplementary File C). In the event of any conflict between the parent and child, the child will not enter the study.

Individual participant medical information obtained as a result of this study is considered confidential and disclosure to third parties is prohibited unless warranted by an adverse event. Participant confidentiality will be further ensured by utilising identification numbers to correspond to treatment data in the computer files. No post-trial care is required; however, participants will have access to the intervention for one-year after starting the treatment, although this access will

For peer review only - http://bmjopen.bmj.com/site/about/guidelines.xhtml

be without therapist support after the 10/12weeks. The Chief Investigator and site principal investigators declare no financial or competing interests. At the end of the trial, the data belongs to the Chief Investigator and the study sponsor. All members of the team will have access to the final trial dataset. The findings will be published in peer-reviewed journals, presented at relevant conferences and disseminated to the public via lay summaries co-created with our PPI group. All outputs will be authored by the research team and will not involve professional writers. Access to the full protocol is available upon request to the corresponding author.

Acknowledgements: ORBIT Trial team members:, Robin Carpenter, Caitlin Hand, Anne Marie Downey, Kareem Khan, Natalia Lago, Jennifer L Martin, and Roz Shafran.

We would like to thank Tourettes Action for their support with the trial, we particularly acknowledge Dr Seonaid Anderson for her ongoing help and advice. We would like to thank our Patient and Public Involvement (PPI) members for their help, including: Debbie Butler, Julie Bryant, James Bungay, Claire Bungay, Sandra Wang, Marco Wang, Alison Neave, Matthew Neave, Rachel Jopling, and Luke Jopling. We thank members of our Trial Steering Committee: Prof Edmund Sonuga-Barke (Chair), Mrs Neelam Gurav, Dr Prathiba Chisabesan, Dr Kim Goldsmith, Dr David Manley, and Dr Cara Verdellen. We also thank members of our Data Safety and Monitoring Committee: Prof Stephen Scott (Chair), Dr Uttom Chowdhury, and Prof Richard Emsley. Finally, we would like to thank Angela Summerfield for her continued support and help.

Author contributions: CH is the Chief Investigator and takes final responsibility for study design, conduct and decision to submit for publication. The study design was conceived and written by CH, CLH (Trial Manager), PA, TM, DMC, EM, LM, MPC, IS, ES, RJ, RH, and CG (co-investigators). CLH wrote the protocol manuscript with approval from all authors. LM and

For peer review only - http://bmjopen.bmj.com/site/about/guidelines.xhtml

RJ (statisticians) designed and wrote the statistical analysis plan. RH (health economist and co-investigator) designed and wrote the health economic plan. CG leads on the process evaluation. EBD (therapist) and TM and JK lead on delivery and design of the therapy. SB (co-investigator) leads on PPI. AMD, RC, NL, JK, MPC, IS, ES (co-investigators) and CS (researcher) and AE (therapist) provided advice and critical input on the study design. All authors critically revised the manuscript for its important intellectual content. All authors read and approved the final manuscript.

Funding statement: This research was funded by the NIHR Heath Technology Assessment (HTA) (Ref 16/19/02). The views expressed are those of the author(s) and not necessarily those of the NHS, the NIHR or the Department of Health and social care. MPC, SB, EBD, CG and CH acknowledge the financial support of the NIHR Nottingham Biomedical Research Centre and NIHR MindTech MedTech Co-operative. The study sponsor and funders have no role in study design, including; collection, management, analysis and interpretation of data; writing of the report; and the decision to submit the report for publication.

Trial Sponsor: Nottinghamshire Healthcare NHS Foundation Trust; Shirley Mitchell. Duncan Macmillan House, Porchester Road, Mapperley, Nottingham, UK, NG3 6AA Shirley.mitchell@nottshc.nhs.uk (Ref: Hollis201117).

Competing interests: On behalf of all authors, the corresponding author declares no competing interests.

Ethical approval: The initial protocol (version 2.0, 26th February 2018) was approved by North West Greater Manchester Research Ethics Committee (REC reference 18/NW/0079) on 23rd March 2018. A subsequent amendment (version 3.0, 16th April 2018) was approved by the

For peer review only - http://bmjopen.bmj.com/site/about/guidelines.xhtml

committee on 15 May 2018 to allow the 10-week intervention to be delivered over a 12-week period to account for periods of therapist absence during bank holidays or unforeseen circumstances.

References

1. Hollis C, Pennant M, Cuenca J, et al. Clinical effectiveness and patient perspectives of different treatment strategies for tics in children and adolescents with Tourette syndrome: a systematic review and qualitative analysis. *Health Technol Asses* 2016;20(4):1-450. DOI: 10.3310/hta20040

2. Freeman RD, Fast DK, Burd L, et al. An international perspective on Tourette syndrome: selected findings from 3500 individuals in 22 countries. *Dev Med Child Neurol* 2000;42(7):436-47. DOI: 10.1111/j.1469-8749.2000.tb00346.x

3. Verdellen C, Van De Griendt J, Hartmann A, et al. European clinical guidelines for Tourette syndrome and other tic disorders. Part III: behavioural and psychosocial interventions. *Eur Child Adoles Psy* 2011;20(4):197-207. DOI: 10.1007/s00787-011-0167-3

4. Whittington C, Pennant M, Kendall T, et al. Practitioner Review: Treatments for Tourette syndrome in children and young people–a systematic review. *J Child Psychol Psyc* 2016;57(9):988-1004. DOI: 10.1111/jcpp.12556

5. Roessner V, Plessen KJ, Rothenberger A, et al. European clinical guidelines for Tourette syndrome and other tic disorders. Part II: pharmacological treatment. *Eur Child Adoles Psy* 2011;20(4):173-96. DOI: 10.1007/s00787-011-0163-7

6. Cuenca J, Glazebrook C, Kendall T, et al. Perceptions of treatment for tics among young people with Tourette syndrome and their parents: a mixed methods study. *BMC Psychiatry* 2015;15(1):46. DOI: 10.1186/s12888-015-0430-0

For peer review only - http://bmjopen.bmj.com/site/about/guidelines.xhtml

7. Verdellen CW, Keijsers GP, Cath DC, et al. Exposure with response prevention versus habit reversal in Tourettes's syndrome: a controlled study. *Behav Res Ther* 2004;42(5):501-11. DOI: 10.1016/S0005-7967(03)00154-2

8. Rizzo R, Pellico A, Silvestri PR, et al. A Randomized Controlled Trial Comparing Behavioral, Educational, and Pharmacological Treatments in Youths With Chronic Tic Disorder or Tourette Syndrome. *Front Psych* 2018;9:100. DOI: 10.3389/fpsyt.2018.00100

9. Andersson G, Cuijpers P, Carlbring P, et al. Guided Internet-based vs. face-to-face cognitive behavior therapy for psychiatric and somatic disorders: a systematic review and meta-analysis. *World Psychiatry* 2014;13(3):288-95. DOI: 10.1002/wps.20151

10. Hedman E, Ljótsson B, Lindefors N. Cognitive behavior therapy via the Internet: a systematic review of applications, clinical efficacy and cost–effectiveness. *Expert Rev Pharm Out* 2012;12(6):745-64. DOI: 10.1586/erp.12.67

11. Palmqvist B, Carlbring P, Andersson G. Internet-delivered treatments with or without therapist input: does the therapist factor have implications for efficacy and cost? *Expert Rev Pharm Out* 2007;7(3):291-97. DOI: 10.1586/14737167.7.3.291

12. Johansson R, Andersson G. Internet-based psychological treatments for depression. *Expert Rev Neurother* 2012;12(7):861-70. DOI: 10.1586/ern.12.63

13. Cuijpers P, Donker T, Johansson R, et al. Self-guided psychological treatment for depressive symptoms: a meta-analysis. *PLoS One* 2011;6(6):e21274. DOI: 10.1371/journal.pone.0021274

14. Vigerland S, Thulin U, Ljótsson B, et al. Internet-delivered CBT for children with specific phobia: a pilot study. *Cogn Behav Therapy* 2013;42(4):303-14. DOI: 10.1016/j.brat.2015.11.006

For peer review only - http://bmjopen.bmj.com/site/about/guidelines.xhtml

15. Vigerland S, Ljótsson B, Thulin U, et al. Internet-delivered cognitive behavioural therapy for children with anxiety disorders: A randomised controlled trial. *Behav Res Ther* 2016;76:47-56. DOI: 10.1016/j.brat.2015.11.006

16. Lenhard F, Vigerland S, Andersson E, et al. Internet-delivered cognitive behavior therapy for adolescents with obsessive-compulsive disorder: an open trial. *PLoS One* 2014;9(6):e100773. DOI: 10.1371/journal.pone.0100773

17. Hollis C, Falconer CJ, Martin JL, et al. Annual Research Review: Digital health interventions for children and young people with mental health problems–a systematic and meta-review. *J Child Psychol Psyc* 2017;58(4):474-503. DOI: 10.1111/jcpp.12663

18. Himle MB, Freitag M, Walther M, et al. A randomized pilot trial comparing videoconference versus face-to-face delivery of behavior therapy for childhood tic disorders. *Behav Res Ther* 2012;50(9):565-70. DOI: 10.1016/j.brat.2012.05.009

19. Ricketts EJ, Goetz AR, Capriotti MR, et al. A randomized waitlist-controlled pilot trial of voice over Internet protocol-delivered behavior therapy for youth with chronic tic disorders. *J Telemed Telecare* 2016;22(3):153-62. DOI: 10.1177/1357633X15593192

20. Andrén P, Aspvall K, Fernández de la Cruz L, et al. Therapist- and parent-guided Internet-delivered behaviour therapy for paediatric Tourette's Disorder: a pilot randomised controlled trial with long-term follow-up. *PsyArXiv (web)* October 21st 2018. DOI:10.31234/osf.io/dp3qz

21. Lau R, Stevenson F, Ong BN, et al. Achieving change in primary care—causes of the evidence to practice gap: systematic reviews of reviews. *Implement Sci* 2016;11(1):40. DOI: 10.1186/s13012-016-0396-4

For peer review only - http://bmjopen.bmj.com/site/about/guidelines.xhtml

22. Kaplan HC, Brady PW, Dritz MC, et al. The influence of context on quality improvement success in health care: a systematic review of the literature. *Milbank Q* 2010;88(4):500-59. DOI: 10.1111/j.1468-0009.2010.00611.x

23. Leckman JF, Riddle MA, Hardin MT, et al. The Yale Global Tic Severity Scale: initial testing of a clinician-rated scale of tic severity. *J Am Acad Child Psy* 1989;28(4):566-73. DOI: 10.1097/00004583-198907000-00015

24. Goodman R, Ford T, Richards H, et al. The Development and Well-Being Assessment: description and initial validation of an integrated assessment of child and adolescent psychopathology. *Journal of Child Psychology and Psychiatry* 2000;41(05):645-55.

25. McKenzie K, Murray GC, Murray AL. The Validity of the Child and Adolescent Intellectual Disability Screening Questionnaire (CAIDS-Q) with children aged 6–7 years, 11 months: A brief report. *Psychiat Res* 2013;210(2):675-77. DOI: 10.1016/j.psychres.2013.06.014

26. Yates R, Edwards K, King J, et al. Habit reversal training and educational group treatments for children with tourette syndrome: a preliminary randomised controlled trial. *Behav Res Ther* 2016;80:43-50. DOI: 10.1016/j.brat.2016.03.003

27. Verdellen C, Van de Griendt J, Kriens S, et al. Tics - Therapist Manual [program]. Amsterdam, Netherlands: Boom Publishers, 2011.

28. Piacentini J, Woods DW, Scahill L, et al. Behavior therapy for children with Tourette disorder: a randomized controlled trial. *JAMA* 2010;303(19):1929-37. DOI: 10.1001/jama.2010.607

29. Chang S, Himle MB, Tucker BT, et al. Initial psychometric properties of a brief parent-report instrument for assessing tic severity in children with chronic tic disorders. *Child Fam Behav Ther* 2009;31(3):181-91. DOI: 10.1080/07317100903099100

For peer review only - http://bmjopen.bmj.com/site/about/guidelines.xhtml

30. Guy W. ECDEU assessment manual for psychopharmacology. *US Department of Health, and Welfare* 1976:534-37.

31. Shaffer D, Gould MS, Brasic J, et al. A children's global assessment scale (CGAS). *Arch Gen Psychiat* 1983;40(11):1228-31. DOI: 10.1001/archpsyc.1983.01790100074010

32. Goodman R. The extended version of the Strengths and Difficulties Questionnaire as a guide to child psychiatric caseness and consequent burden. *Journal of Child Psychology and Psychiatry* 1999;40(5):791-99.

33. Angold A, Costello EJ, Messer SC, et al. Development of a short questionnaire for use in epidemiological studies of depression in children and adolescents. *Int J Meth Psych Res* 1995; *5*(4), 237-249.

34. Spence SH. A measure of anxiety symptoms among children. *Behav Res Ther* 1998;36(5):545-66. DOI: 10.1016/S0005-7967(98)00034-5

35. Stevens K. The Child Health Utility 9D (CHU9D). *A new, paediatric, preference-based measure of health related quality of life PRO Newsletter* 2010;43:11-2.

36. Cavanna AE, Luoni C, Selvini C, et al. Disease-specific quality of life in young patients with Tourette syndrome. *Pediatr Neurol* 2013;48(2):111-14. DOI: 10.1016/j.pediatrneurol.2012.10.006

37. Beecham J, Knapp M. Costing Psychiatric Interventions. In: Thornicroft G, Brewin C, Wing J, eds. Measuring mental health needs. London: Gaskell 2001:200-24.

38. Byford S, Barrett B, Roberts C, et al. Cost-effectiveness of selective serotonin reuptake inhibitors and routine specialist care with and without cognitive–behavioural therapy in adolescents with major depression. *Brit J Psychiat* 2007;191(6):521-27. DOI: 10.1192/bjp.bp.107.038984

For peer review only

39. Hill P, Taylor E. An auditable protocol for treating attention deficit/hyperactivity disorder. *Arch Dis Child* 2001;84(5):404-09. DOI: 10.1136/adc.84.5.404

40. Rutter M, Bailey A, Lord C. Social Communication Questionnaire (SCQ). Los Angeles: Western Psychological Services 2003.

41. Woods DW, Piacentini J, Himle MB, et al. Premonitory Urge for Tics Scale (PUTS): initial psychometric results and examination of the premonitory urge phenomenon in youths with Tic disorders. *J Dev Behav Pediatr* 2005;26(6):397-403.

42. Swanson JM, Kraemer HC, Hinshaw SP, et al. Clinical relevance of the primary findings of the MTA: success rates based on severity of ADHD and ODD symptoms at the end of treatment. *J Am Acad Child Psy* 2001;40(2):168-79. DOI: 10.1097/00004583-200102000-00011

43. Craig P, Dieppe P, Macintyre S, et al. Developing and evaluating complex interventions: the new Medical Research Council guidance. *BMJ* 2008;337:a1655. DOI: https://doi.org/10.1136/bmj.a1655

44. Moore GF, Audrey S, Barker M, et al. Process evaluation of complex interventions: Medical Research Council guidance. *BMJ* 2015;350. DOI: 10.1136/bmj.h1258

45. Braun V, Clarke V. Using thematic analysis in psychology. *Qual Res Psychol* 2006;3(2):77-101.DOI: 10.1191/1478088706qp063oa

FIGURE CAPTION:

Figure 1: Schematic flow diagram of trial design

Key: CAIDS-Q = Child and Adolescent Intellectual Disability Screening Questionnaire; CTU = Clinical Trials Unit; DAWBA = Development and Wellbeing Assessment; YGTSS = Yale Global Tic Severity Scale.

For peer review only - http://bmjopen.bmj.com/site/about/guidelines.xhtml

Figure 2: Participant flow chart

Key: C&A-GTS-QOL = Child and adolescent version of the Gilles de la Tourette Syndrome Quality of Life Scale; CAIDS-Q = Child and Adolescent Intellectual Disability Screening Questionnaire; CGAS = Children's Global Assessment Scale; CGI-I = Clinical Global Impressions- Improvement; CHU9D = Child Health Utility 9D; CSRI = Client Service Receipt Inventory; DAWBA = Development and Wellbeing Assessment; MFQ = Mood and Feelings Questionnaire; PTQ = Parent Tic Questionnaire; PUTS = Premonitory Urge for Tics Scale; RT = Research team; SCAS = Spence Child Anxiety Scale; SCQ = Social Communication Questionnaire; SNAP-IV; Swanson, Nolan and Pelham Rating Scale; SDQ = Strengths and Difficulties Questionnaire; YGTSS = Yale Global Tic Severity Scale.

Note: The CSRI includes a measure of school attendance. The YGTSS includes both a measure of severity and impairment.

For peer review only

For peer review only - http://bmjopen.bmj.com/site/about/guidelines.xhtml

Figure 1

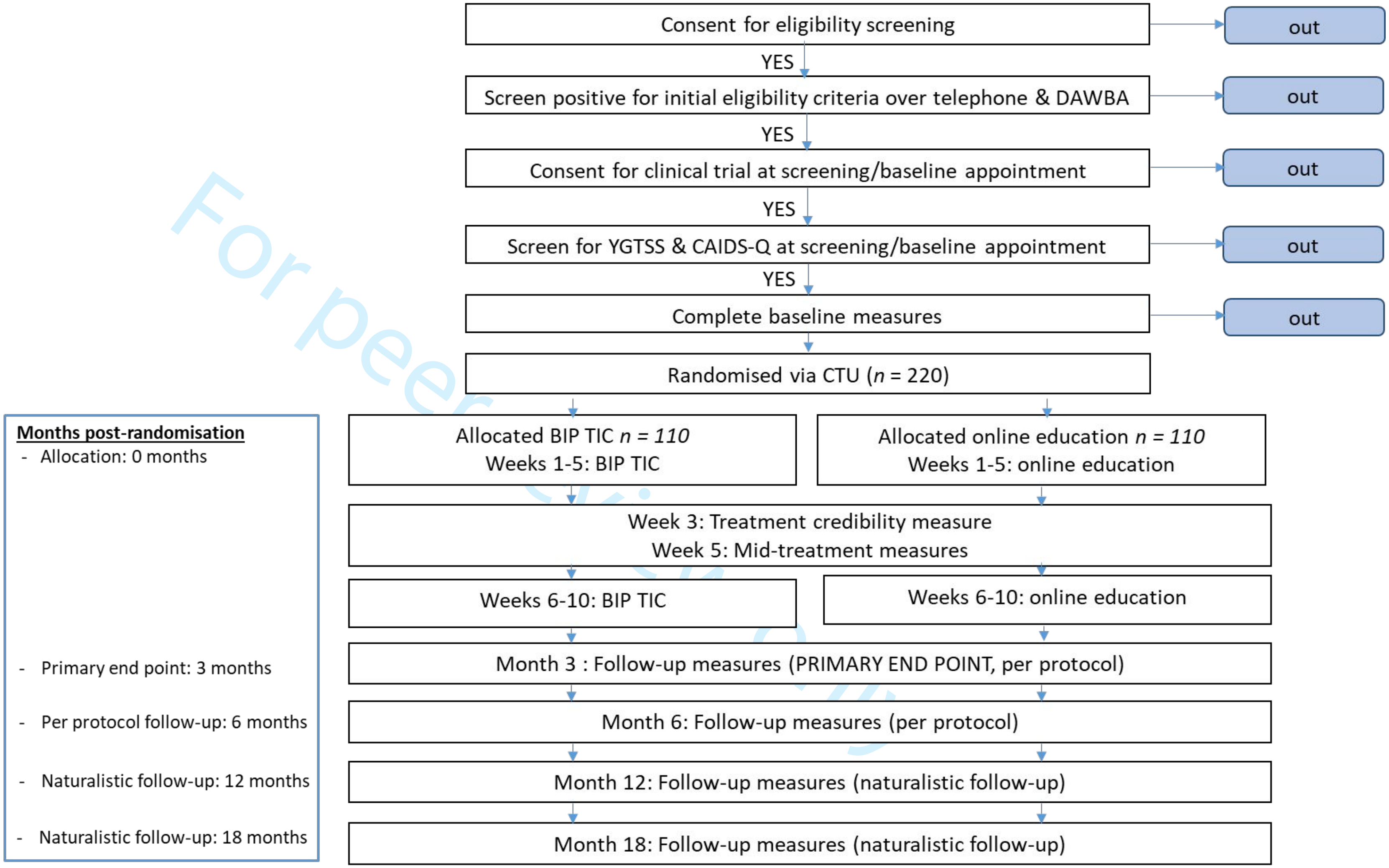

Key: CAIDS-Q = Child and Adolescent Intellectual Disability Screening Questionnaire; CTU = Clinical Trials Unit; DAWBA = Development and Wellbeing Assessment; YGTSS = Yale Global Tic Severity Scale.

Figure 2

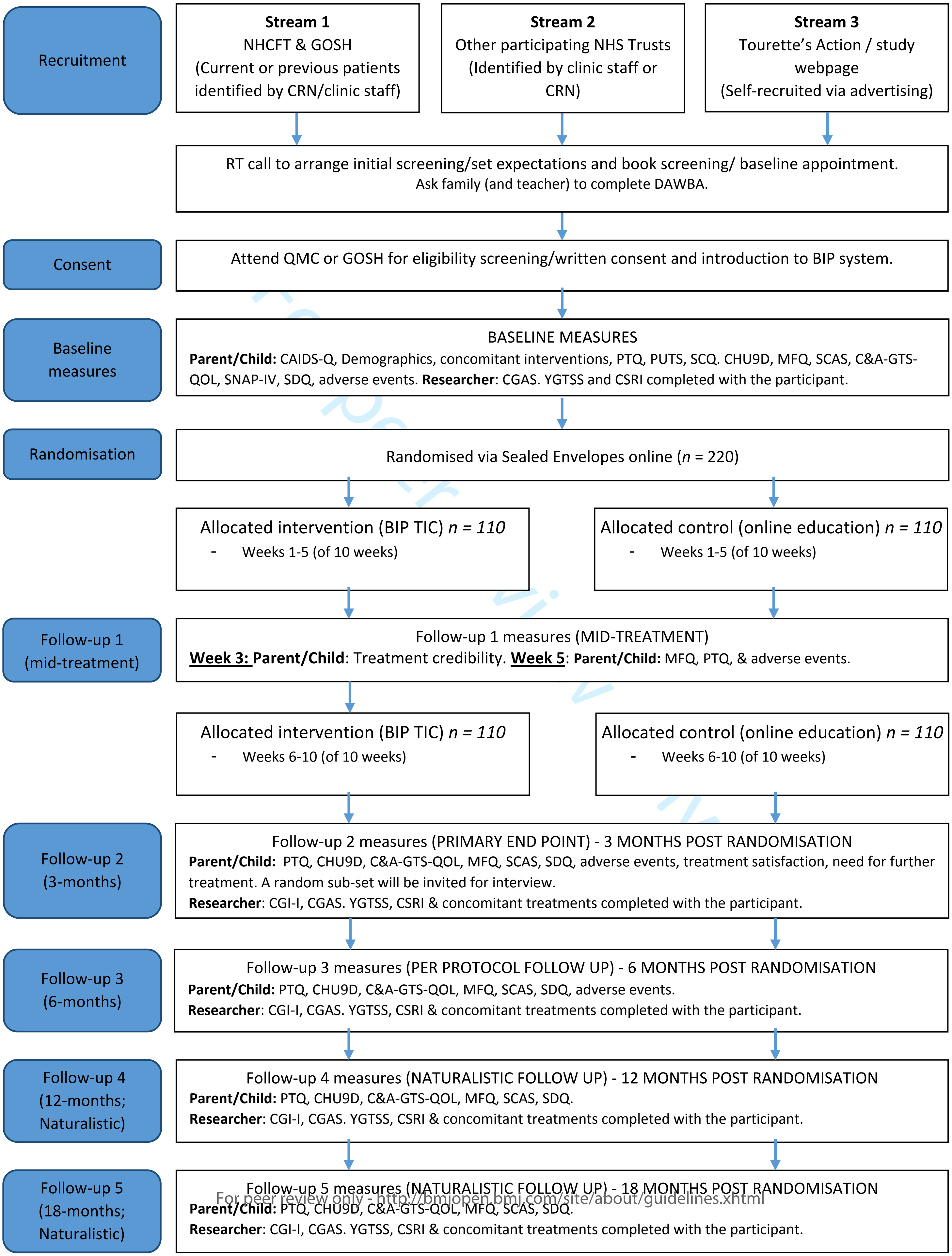

For peer review only - http://bmjopen.bmj.com/site/about/guidelines.xhtml

Figure 2

Key: C&A-GTS-QOL = Child and adolescent version of the Gilles de la Tourette Syndrome Quality of Life Scale; CAIDS-Q = Child and Adolescent Intellectual Disability Screening Questionnaire; CGAS = Children's Global Assessment Scale; CGI-I = Clinical Global Impressions- Improvement; CHU9D = Child Health Utility 9D; CSRI = Client Service Receipt Inventory; DAWBA = Development and Wellbeing Assessment; MFQ = Mood and Feelings Questionnaire; PTQ = Parent Tic Questionnaire; PUTS = Premonitory Urge for Tics Scale; RT = Research team; SCAS = Spence Child Anxiety Scale; SCQ = Social Communication Questionnaire; SNAP-IV; Swanson, Nolan and Pelham Rating Scale; SDQ = Strengths and Difficulties Questionnaire; YGTSS = Yale Global Tic Severity Scale.

Note: The CSRI includes a measure of school attendance. The YGTSS includes both a measure of severity and impairment.

For peer review only

Supplementary File A: Consent for parent/carers

CONSENT FORM FOR PARENTS/CARERS

Centre Name: Nottinghamshire Healthcare NHS Foundation Trust

REC reference: 18/NW/0079

Participant Identification Number for this trial:

CONSENT FORM

Title of Project: Online Remote Behavioural Intervention for Tics (ORBIT)

Name of Researcher:

Please initial box

1. I confirm that I have read the information sheet dated ______ (version__) for the above study. I have had the opportunity to consider the information, ask questions and have had these answered satisfactorily. ☐

2. I understand that mine and my child's participation is voluntary and that I am free to withdraw at any time without giving any reason, without my medical care or legal rights being affected. ☐

3. I understand that relevant sections of my child's medical notes and data collected during the study, may be looked at by individuals from the ORBIT team, from regulatory authorities or from the NHS Trust, where it is relevant to my taking part in this research. ☐
   I give permission for these individuals to have access to my records. I understand this data will be stored in the UK (Sealed Envelopes) and Sweden (BIP and BASS) secure databases and servers.

4. I understand that the information collected about me and my child will be used to support other research in the future, and may be shared anonymously with other researchers. ☐

5. I agree to my child's General Practitioner being informed of our participation in the study. ☐

For peer review only - http://bmjopen.bmj.com/site/about/guidelines.xhtml

Supplementary File A: Consent for parent/carers

6. I understand that I/my child may be asked to take part in research interviews, which will be recorded and anonymous direct quotes from these interviews may be used in study reports. ☐

7. I agree for me and my child (named below) to take part in the above study. ☐

For peer review only

Name of Parent/carer	Date	Signature

Name of child

Name of Person taking consent	Date	Signature

When completed: 1 for participant; 1 for researcher site file; 1 (original) to be kept in medical notes.

This research was funded by the NIHR Health Technology Assessment (ref 16/19/02). The views expressed are those of the author(s) and not necessarily those of the NHS, the NIHR or the Department of Health.

For peer review only - http://bmjopen.bmj.com/site/about/guidelines.xhtml

Supplementary File B: Consent form for young people aged 16-years and over

CONSENT FORM FOR YOUNG PEOPLE 16 YEARS AND OVER

Centre Name: Nottinghamshire Healthcare NHS Foundation Trust

REC reference: 18/NW/0079

Participant Identification Number for this trial:

CONSENT FORM

Title of Project: Online Remote Behavioural Intervention for Tics (ORBIT)

Name of Researcher:

Please initial box

1. I confirm that I have read the information sheet dated _____ (version___) for the above study. I have had the opportunity to consider the information, ask questions and have had these answered satisfactorily. ☐

2. I understand that my participation is voluntary and that I am free to withdraw at any time without giving any reason, without my medical care or legal rights being affected. ☐

3. I understand that relevant sections of my medical notes and data collected during the study may be looked at by individuals from the ORBIT team, from regulatory authorities or from the NHS Trust, where it is relevant to my taking part in this research. I give permission for these individuals to have access to my records. I understand this data will be stored in the UK (Sealed Envelopes) and Sweden (BIP and BASS) secure databases and servers. ☐

4. I understand that the information collected about me will be used to support other researc in the future, and may be shared anonymously with other researchers. ☐

5. I agree to my General Practitioner being informed of my participation in the study. ☐

For peer review only - http://bmjopen.bmj.com/site/about/guidelines.xhtml

Supplementary File B: Consent form for young people aged 16-years and over

6. I understand that I may be asked to take part in research interviews, which will be recorded and anonymous direct quotes from these interviews may be used in study reports. ☐

7. I agree to take part in the above study. ☐

Name of Participant	Date	Signature
Name of Person taking consent	Date	Signature

When completed: 1 for participant; 1 for researcher site file; 1 (original) to be kept in medical notes.

This research was funded by the NIHR Health Technology Assessment (ref 16/19/02). The views expressed are those of the author(s) and not necessarily those of the NHS, the NIHR or the Department of Health.

For peer review only - http://bmjopen.bmj.com/site/about/guidelines.xhtml

For peer review only

Supplementary File C: Assent form for young people aged under 16 years

ASSENT FORM FOR YOUNG PEOPLE UNDER 16 YEARS

Centre Name: Nottinghamshire Healthcare NHS Foundation Trust

REC reference: 18/NW/0079

Participant Identification Number for this trial:

ASSENT FORM

Title of Project: Online Remote Behavioural Intervention for Tics (ORBIT)

Name of Researcher:

Please initial box

1. I have read the information sheet dated ______ (version___) for the ORBIT study. I have discussed it with my mum/dad/carer and the researcher and I have asked questions. ☐

2. I understand that I don't have to take part and I can stop taking part any time. This is my choice and no-one will be upset with me if I stop. ☐

3. I understand that the ORBIT team may look at my medical records and the data will be kept in a database both in England and in Sweden. This will be kept safe and only the research team will see my data. ☐

4. I understand that the research team will write a report about the project. My name will no[illegible] be mentioned in any reports. ☐

5. I agree to my Doctor knowing that I am taking part in the ORBIT study. ☐

6. The researcher might ask me to take part in an interview about my experiences of the ORB[illegible] trial. I do not have to take part. If I agree to take part, the interview will be recorded but on[illegible] the research team will know that I did the interview. ☐

For peer review only - http://bmjopen.bmj.com/site/about/guidelines.xhtml

Supplementary File C: Assent form for young people aged under 16 years

7. I agree to take part in the ORBIT study. ☐

Name of young person	Date	Signature
Name of Person taking assent	Date	Signature

When completed: 1 for participant; 1 for researcher site file; 1 (original) to be kept in medical notes.

This research was funded by the NIHR Health Technology Assessment (ref 16/19/02). The views expressed are those of the author(s) and not necessarily those of the NHS, the NIHR or the Department of Health.

For peer review only

For peer review only - http://bmjopen.bmj.com/site/about/guidelines.xhtml

STANDARD PROTOCOL ITEMS: RECOMMENDATIONS FOR INTERVENTIONAL TRIALS

SPIRIT 2013 Checklist: Recommended items to address in a clinical trial protocol and related documents*

Section/item	Item No	Description	Addressed on page number
**Administrative information**			
Title	1	Descriptive title identifying the study design, population, interventions, and, if applicable, trial acronym	___1_______
Trial registration	2a	Trial identifier and registry name. If not yet registered, name of intended registry	___4+10____
	2b	All items from the World Health Organization Trial Registration Data Set	____4+10___
Protocol version	3	Date and version identifier	_____26___
Funding	4	Sources and types of financial, material, and other support	____28+29____
Roles and responsibilities	5a	Names, affiliations, and roles of protocol contributors	___1+2____
	5b	Name and contact information for the trial sponsor	____26 + 29____
	5c	Role of study sponsor and funders, if any, in study design; collection, management, analysis, and interpretation of data; writing of the report; and the decision to submit the report for publication, including whether they will have ultimate authority over any of these activities	____26+28__
	5d	Composition, roles, and responsibilities of the coordinating centre, steering committee, endpoint adjudication committee, data management team, and other individuals or groups overseeing the trial, if applicable (see Item 21a for data monitoring committee)	___25+26____

For peer review only

For peer review only - http://bmjopen.bmj.com/site/about/guidelines.xhtml

**Introduction**			___________
Background and rationale	6a	Description of research question and justification for undertaking the trial, including summary of relevant studies (published and unpublished) examining benefits and harms for each intervention	__5-6+8____
	6b	Explanation for choice of comparators	____9+14_____
Objectives	7	Specific objectives or hypotheses	____8-9_____
Trial design	8	Description of trial design including type of trial (eg, parallel group, crossover, factorial, single group), allocation ratio, and framework (eg, superiority, equivalence, noninferiority, exploratory)	___9-10____
**Methods: Participants, interventions, and outcomes**			
Study setting	9	Description of study settings (eg, community clinic, academic hospital) and list of countries where data will be collected. Reference to where list of study sites can be obtained	___10_____
Eligibility criteria	10	Inclusion and exclusion criteria for participants. If applicable, eligibility criteria for study centres and individuals who will perform the interventions (eg, surgeons, psychotherapists)	____11____
Interventions	11a	Interventions for each group with sufficient detail to allow replication, including how and when they will be administered	____12-15__
	11b	Criteria for discontinuing or modifying allocated interventions for a given trial participant (eg, drug dose change in response to harms, participant request, or improving/worsening disease)	____NA_____
	11c	Strategies to improve adherence to intervention protocols, and any procedures for monitoring adherence (eg, drug tablet return, laboratory tests)	_____15_____
	11d	Relevant concomitant care and interventions that are permitted or prohibited during the trial	_____11_____
Outcomes	12	Primary, secondary, and other outcomes, including the specific measurement variable (eg, systolic blood pressure), analysis metric (eg, change from baseline, final value, time to event), method of aggregation (eg, median, proportion), and time point for each outcome. Explanation of the clinical relevance of chosen efficacy and harm outcomes is strongly recommended	15-21+Table 1
Participant timeline	13	Time schedule of enrolment, interventions (including any run-ins and washouts), assessments, and visits for participants. A schematic diagram is highly recommended (see Figure)	__Fig1,2, Table 1

For peer review only - http://bmjopen.bmj.com/site/about/guidelines.xhtml

Sample size	14	Estimated number of participants needed to achieve study objectives and how it was determined, including clinical and statistical assumptions supporting any sample size calculations	____22____
Recruitment	15	Strategies for achieving adequate participant enrolment to reach target sample size	__22___

Methods: Assignment of interventions (for controlled trials)

Allocation:			___22-23_____
Sequence generation	16a	Method of generating the allocation sequence (eg, computer-generated random numbers), and list of any factors for stratification. To reduce predictability of a random sequence, details of any planned restriction (eg, blocking) should be provided in a separate document that is unavailable to those who enrol participants or assign interventions	___22-23_____
Allocation concealment mechanism	16b	Mechanism of implementing the allocation sequence (eg, central telephone; sequentially numbered, opaque, sealed envelopes), describing any steps to conceal the sequence until interventions are assigned	___22-23_____
Implementation	16c	Who will generate the allocation sequence, who will enrol participants, and who will assign participants to interventions	____22-23____
Blinding (masking)	17a	Who will be blinded after assignment to interventions (eg, trial participants, care providers, outcome assessors, data analysts), and how	____22-23____
	17b	If blinded, circumstances under which unblinding is permissible, and procedure for revealing a participant's allocated intervention during the trial	___22-23_____

Methods: Data collection, management, and analysis

Data collection methods	18a	Plans for assessment and collection of outcome, baseline, and other trial data, including any related processes to promote data quality (eg, duplicate measurements, training of assessors) and a description of study instruments (eg, questionnaires, laboratory tests) along with their reliability and validity, if known. Reference to where data collection forms can be found, if not in the protocol	__15-21____
	18b	Plans to promote participant retention and complete follow-up, including list of any outcome data to be collected for participants who discontinue or deviate from intervention protocols	__23-24_____

Data management	19	Plans for data entry, coding, security, and storage, including any related processes to promote data quality (eg, double data entry; range checks for data values). Reference to where details of data management procedures can be found, if not in the protocol	___23-24_____
Statistical methods	20a	Statistical methods for analysing primary and secondary outcomes. Reference to where other details of the statistical analysis plan can be found, if not in the protocol	___23/25____
	20b	Methods for any additional analyses (eg, subgroup and adjusted analyses)	___24_____
	20c	Definition of analysis population relating to protocol non-adherence (eg, as randomised analysis), and any statistical methods to handle missing data (eg, multiple imputation)	___24_____
**Methods: Monitoring**			
Data monitoring	21a	Composition of data monitoring committee (DMC); summary of its role and reporting structure; statement of whether it is independent from the sponsor and competing interests; and reference to where further details about its charter can be found, if not in the protocol. Alternatively, an explanation of why a DMC is not needed	___25____
	21b	Description of any interim analyses and stopping guidelines, including who will have access to these interim results and make the final decision to terminate the trial	____9-10 + 25___
Harms	22	Plans for collecting, assessing, reporting, and managing solicited and spontaneously reported adverse events and other unintended effects of trial interventions or trial conduct	_____19+26____
Auditing	23	Frequency and procedures for auditing trial conduct, if any, and whether the process will be independent from investigators and the sponsor	____26______
**Ethics and dissemination**			
Research ethics approval	24	Plans for seeking research ethics committee/institutional review board (REC/IRB) approval	_____26-27___
Protocol amendments	25	Plans for communicating important protocol modifications (eg, changes to eligibility criteria, outcomes, analyses) to relevant parties (eg, investigators, REC/IRBs, trial participants, trial registries, journals, regulators)	____27_____

For peer review only - http://bmjopen.bmj.com/site/about/guidelines.xhtml

Consent or assent	26a	Who will obtain informed consent or assent from potential trial participants or authorised surrogates, and how (see Item 32)	____26-27____
	26b	Additional consent provisions for collection and use of participant data and biological specimens in ancillary studies, if applicable	____26-27____
Confidentiality	27	How personal information about potential and enrolled participants will be collected, shared, and maintained in order to protect confidentiality before, during, and after the trial	___27____
Declaration of interests	28	Financial and other competing interests for principal investigators for the overall trial and each study site	____27+29____
Access to data	29	Statement of who will have access to the final trial dataset, and disclosure of contractual agreements that limit such access for investigators	___27_______
Ancillary and post-trial care	30	Provisions, if any, for ancillary and post-trial care, and for compensation to those who suffer harm from trial participation	_NA (see p27)__
Dissemination policy	31a	Plans for investigators and sponsor to communicate trial results to participants, healthcare professionals, the public, and other relevant groups (eg, via publication, reporting in results databases, or other data sharing arrangements), including any publication restrictions	___26-27_____
	31b	Authorship eligibility guidelines and any intended use of professional writers	___27_____
	31c	Plans, if any, for granting public access to the full protocol, participant-level dataset, and statistical code	___NA______
**Appendices**			
Informed consent materials	32	Model consent form and other related documentation given to participants and authorised surrogates	___Appendix A,B,C_______
Biological specimens	33	Plans for collection, laboratory evaluation, and storage of biological specimens for genetic or molecular analysis in the current trial and for future use in ancillary studies, if applicable	___NA_______

*It is strongly recommended that this checklist be read in conjunction with the SPIRIT 2013 Explanation & Elaboration for important clarification on the items. Amendments to the protocol should be tracked and dated. The SPIRIT checklist is copyrighted by the SPIRIT Group under the Creative Commons "Attribution-NonCommercial-NoDerivs 3.0 Unported" license.

For peer review only - http://bmjopen.bmj.com/site/about/guidelines.xhtml

BMJ Open

Investigating a therapist-guided, parent-assisted remote digital behavioural intervention for tics in children and adolescents – 'Online Remote Behavioural Intervention for Tics' (ORBIT) Trial: protocol of an internal pilot study and single-blind randomised controlled trial

Journal:	*BMJ Open*
Manuscript ID	bmjopen-2018-027583.R1
Article Type:	Protocol
Date Submitted by the Author:	15-Nov-2018

Complete List of Authors:	Hall, Charlotte; Institute of Mental Health, School of Medicine, University of Nottingham<br>Davies, E. Bethan; University of Nottingham, School of Medicine<br>Andrén, Per; Karolinska Institutet Department of Clinical Neuroscience, Department of Clinical Neuroscience<br>Murphy, Tara; UCL Great Ormond Street Institute of Child Health<br>Bennett, Sophie; UCL Great Ormond Street Institute of Child Health, ; Institute of Child Health<br>Brown, Beverley; University of Nottingham, School of Medicine<br>Brown, Sue; University of Nottingham<br>Chamberlain, Liam; Queen's Medical Centre<br>Craven, Michael; Institute of Mental Health, University of Nottingham<br>Evans, Amber; UCL Great Ormond Street Institute of Child Health<br>Glazebrook, Cristine; University of Nottingham, Psychiatry<br>Heyman, Isobel; Great Ormond Street Hospital, Psychological Medicine Team, Child and Adolescent Mental Health Service<br>Hunter, Rachael; University College London, Research Dept of Primary Care and Population Health<br>Jones, Rebecca; UCL<br>Kilgariff, Joseph; Queen's Medical Centre<br>Marston, Louise; University College London,<br>Mataix-Cols, David; Karolinska Institutet, Department of Clinical Neuroscience<br>Murray, Elizabeth; University College London, Primary Care and Population Health<br>Sanderson, Charlotte; UCL Great Ormond Street Institute of Child Health<br>Serlachius , Eva ; Karolinska Institutet,<br>Hollis, Chris; University of Nottingham, Division of Psychiatry

**Primary Subject Heading**:	Mental health
Secondary Subject Heading:	Mental health, Paediatrics
Keywords:	behaviour therapy, exposure and response prevention, internet, Persistent (Chronic) Motor or Vocal Tic Disorder, Tourette's Disorder

For peer review only - http://bmjopen.bmj.com/site/about/guidelines.xhtml

Investigating a therapist-guided, parent-assisted remote digital behavioural intervention for tics in children and adolescents – 'Online Remote Behavioural Intervention for Tics' (ORBIT) Trial: protocol of an internal pilot study and single-blind randomised controlled trial

Authors

Charlotte L. Hall, Trial Manager, Institute of Mental Health, University of Nottingham, Nottingham, UK

E. Bethan Davies, Research Fellow and Trial Therapist, NIHR MindTech Medtech Co-operative, Institute of Mental Health, University of Nottingham, Nottingham, UK

Per Andrén, Clinical Psychologist, Department of Clinical Neuroscience, Karolinska Institutet, Stockholm, Sweden and Stockholm Health Care Services, Stockholm County Council, Sweden

Tara Murphy, Consultant Psychologist, University College London, Great Ormond Street Hospital for Children NHS Foundation Trust, Great Ormond Street, London, UK

Sophie Bennett, Senior Research Fellow, University College London, Great Ormond Street Hospital for Children NHS Foundation Trust, Great Ormond Street, London, UK

Beverley J Brown, Research Fellow, Institute of Mental Health, University of Nottingham, Nottingham, UK

Susan Brown, Research Fellow, NIHR MindTech Medtech Co-operative, Institute of Mental Health, University of Nottingham, Nottingham, UK

Liam Chamberlain, Trial Therapist, Division of Psychiatry and Applied Psychology, University of Nottingham, Queen's Medical Centre, Nottingham, UK

Michael P. Craven, Senior Research Fellow, NIHR MindTech Medtech Co-operative, Institute of Mental Health, University of Nottingham, Nottingham, UK

Amber Evans, Trial Therapist, University College London, Great Ormond Street Hospital for Children NHS Foundation Trust, Great Ormond Street, London, UK

Christine Glazebrook, Professor of Health Psychology, Division of Psychiatry and Applied Psychology, Institute of Mental Health, University of Nottingham, Nottingham, UK

Isobel Heyman, Consultant Psychiatrist, Great Ormond Street Hospital for Children NHS Foundation Trust, Great Ormond Street, London, UK

Rachael Hunter, Health Economist, Research Department of Primary care and Population health and Priment CTU, University College London, Royal Free Campus, London, UK

Rebecca Jones, Senior Research Associate, Research Department of Primary care and Population health and Priment CTU, University College London, Royal Free Campus, London, UK

For peer review only - http://bmjopen.bmj.com/site/about/guidelines.xhtml

Joseph Kilgariff, Advanced Nurse, Division of Psychiatry and Applied Psychology, University of Nottingham, Queen's Medical Centre, Nottingham, UK

Louise Marston, Principal Research Associate, Research Department of Primary care and Population Health and Priment CTU, University College London, Royal Free Campus, London, UK

David Mataix-Cols, Professor of Child and Adolescent Psychiatric Science, Department of Clinical Neuroscience, Karolinska Institutet, Stockholm, Sweden and Stockholm Health Care Services, Stockholm County Council, Sweden

Elizabeth Murray, Professor of eHealth and Primary Care, eHealth Unit, Research Department of Primary Care and Population Health, University College London, Royal Free Campus, London, UK

Charlotte Sanderson, Research Clinical Psychologist, University College London, Great Ormond Street Hospital for Children NHS Foundation Trust, Great Ormond Street, London, UK

Eva Serlachius, Assistant Professor in Child and Adolescent Psychiatry, Department of Clinical Neuroscience, Karolinska Institutet, Stockholm, Sweden and Stockholm Health Care Services, Stockholm County Council, Sweden

Chris Hollis, Professor of Child & Adolescent Psychiatry, Developmental Psychiatry, University of Nottingham, Queens Medical Centre, Nottingham, UK

On behalf of the ORBIT Trial team

Corresponding Author
Chris Hollis (*PhD, FRCPsych)*
Institute of Mental Health, University of Nottingham, Triumph Road, Nottingham, NG7 2TU.
Email: chris.hollis@nottingham.ac.uk Tel: 0115 823 0258

Key words: behaviour therapy; exposure and response prevention; Internet; Persistent (Chronic) Motor or Vocal Tic Disorder; Tourette's Disorder

Word count: 4,254

For peer review only

ABSTRACT

Introduction

Tourette syndrome and chronic tic disorder are common, disabling childhood-onset conditions. Guidelines recommend that behavioural therapy should be offered as first-line treatment for children with tics. However, there are very few trained behaviour therapists for tics and many patients cannot access appropriate care. This trial investigates whether an internet-delivered intervention for tics can reduce severity of symptoms.

Method and analysis

This parallel-group, single-blind, randomised controlled superiority trial with an internal pilot will recruit children and young people (aged 9-17 years) with tic disorders. Participants will be randomised to receive 10-weeks of either online, remotely-delivered, therapist-supported exposure response prevention (ERP) behavioural therapy for tics, or online, remotely-delivered, therapist-supported education about tics and co-occurring conditions. Participants will be followed-up mid-treatment, and 3-, 6-, 12-, and 18-month post-randomisation.

The primary outcome is reduction in tic severity as measured on the Yale Global Tic Severity Scale (YGTSS) total tic severity score. Secondary outcomes include a cost-effectiveness analysis and estimate of the longer-term impact on patient outcomes and healthcare services. An integrated process evaluation will analyse quantitative and qualitative data in order to fully explore the implementation of the intervention and identify barriers and facilitators to implementation. The trial is funded by the National Institute of Health Research (NIHR), Health Technology Assessment (16/19/02).

Ethics and dissemination

The findings from the study will inform clinicians, healthcare providers and policy makers about the clinical and cost-effectiveness of an internet delivered treatment for children and

For peer review only

For peer review only - http://bmjopen.bmj.com/site/about/guidelines.xhtml

young people with tics. The results will be submitted for publication in peer-reviewed journals. The study has received ethical approval from North West Greater Manchester Research Ethics Committee (Ref: 18/NW/0079).

Trial registration: ISRCTN70758207 and ClinicalTrials.gov (NCT03483493).

STRENGTHS AND LIMITATIONS OF THIS STUDY

- This study is a 10-week, parallel-group, single blind, non-commercial, randomised controlled superiority trial with an internal pilot; the first large trial for internet delivered therapy for tics in the UK.
- The study design and methodology are based upon an initial pilot study of internet-delivered ERP for tics conducted in Sweden.
- The protocol was created with a multidisciplinary team of experts including, healthcare professionals, patient and public involvement members, and health service researchers.
- This trial is only available to English-speaking participants with access to a computer/internet.

For peer review only

INTRODUCTION

Tourette syndrome (TS) and chronic tic disorder (CTD) are common, disabling, childhood-onset conditions affecting up to 1% of young people and are associated with significant distress, psychosocial impairment and reduced quality of life. [1] The majority of patients additionally experience comorbidities such as attention deficit hyperactivity disorder (ADHD), obsessive-compulsive disorder (OCD), depression, anxiety, and self-injurious behaviour. [2] Evidence-based interventions for the treatment of tics in children and adolescents with TS include pharmacological treatment and behaviour therapy. [1 3-5] European clinical guidelines[3] and a National Institute of Health Research (NIHR) Health Technology Assessment (HTA) Evidence Synthesis[1] recommend that behaviour therapy should be offered as a first-line intervention for tics in children and adolescents in a stepped-care approach.

Despite these recommendations, only 1 in 5 young people with TS are currently able to access behaviour therapy for tics, while around 50% receive medication,[6] which is associated with a significant risk of adverse effects including weight gain and sedation.[4 5] Furthermore, those young people who manage to access behaviour therapy typically receive four or fewer face-to-face therapy sessions,[6] less than half the number recommended.[7] Commonly used behaviour therapies include: *habit reversal training (HRT),* whereby patients are taught to implement and maintain an action referred to as a 'competing response' when a premonitory urge to tic is experienced (the urge to tic often felt before the tic is expressed); *comprehensive behavioural intervention for tics (CBIT),* which includes HRT and adds additional elements of relaxation training, functional analyses (identification of situations which could exacerbate tics) and social support; and *exposure and response prevention (ERP),* where patients tolerate premonitory urge sensations and learn from their therapist how to resist their tics.

For peer review only

The effectiveness of behavioural therapy for reducing tics has been well established,[1] with systematic reviews demonstrating a similar magnitude of effect for HRT/CBIT compared to pharmaceutical interventions.[1, 4] A recent trial compared 10 weeks of behavioural therapy (either HRT or ERP) with education and pharmacological treatment and found the behavioural therapy to be as effective as pharmacological treatment for reducing tic severity, with both being superior to education.[8] Additionally, although there is a lack of evidence from randomised controlled trials (RCTs) for ERP, the evidence from available studies suggests both HRT and ERP may be at least equally effective in reducing tics, with some evidence showing a tendency to favour ERP.[3] The effectiveness of these behavioural interventions in conjunction with the lack of side effects compared to pharmacotherapy supports the use of behaviour therapy as a first-line intervention.. However, there is a lack of trained therapists across many European countries, leaving many patients unable to access evidence-based behaviour therapy. This need for improved access to therapy was highlighted in a recent study whereby families of children with TS noted the struggle to access limited behavioural therapy resources, despite 76% of parents saying they would want behavioural therapy to be available for their child.[6]

Over the last decade, internet-delivered cognitive behaviour therapy (ICBT) has developed, enabling the delivery of effective, but less therapist intensive, cognitive and behavioural interventions remotely, which can increase the availability of evidence based treatments. Across diagnostic conditions, studies have not only shown efficacy of ICBT compared to no-treatment control conditions, but meta analyses comparing face-to-face delivered CBT with ICBT have demonstrated comparable results in terms of symptom reduction for adults with mental health problems,[9 ,10] which could also result in 50% cost savings.[10] However, therapist guidance during ICBT has been shown to be an important contributing factor in determining

For peer review only - http://bmjopen.bmj.com/site/about/guidelines.xhtml

outcome.[11] Although self-guided ICBT is superficially attractive, given the very low costs of implementation, research evidence demonstrates very low adherence rates.[12, 13]

Recently, researchers at the Karolinska Institutet, Sweden, have developed a technical platform for ICBT called BIP (*Barninternetprojektet,* Swedish for *Child Internet Project*; http://www.bup.se/bip). The platform has been used to deliver therapist-supported ICBT for a range of different conditions, including phobia,[14] anxiety,[15] and obsessive-compulsive disorder.[16] The findings support the use of therapist guided ICBT using the BIP system, however, there is little research evidence with regards to the effectiveness of ICBT and TS. In a recent systematic review of digital health interventions, Hollis and colleagues[17] found the majority of interventions have been designed to help children and young people at risk for developing, or with a diagnosis of, an anxiety disorder and/or depression, with areas such as TS being largely overlooked. Innovations in behavioural therapies for tics include delivery of CBIT treatment remotely via video over the internet with a therapist. Two pilot RCTs have compared CBIT delivered remotely via videoconference to traditional face-to-face delivery in children with TS. Results showed significant tic reduction for both groups, with no difference between the modes of delivery.[18, 19] The method of delivering CBIT was rated as highly acceptable by the participants.[18]

One Swedish pilot study, upon which this current trial is based, has used the BIP platform to deliver ICBT with remote therapist support. The study compared tic reduction in children aged 8-16 years randomised to receive either ERP or HRT delivered online via the BIP platform.[20] Participants in both groups showed improvement after treatment completion, but only participants in the ERP arm showed significant reduction on the primary outcome measure Yale Global Tic Severity Scale (YGTSS) total tic severity score. . The gains were maintained for at least 12 months after the end of treatment. Although this study was not designed or powered as a definitive efficacy trial, these findings show that ERP treatment

For peer review only - http://bmjopen.bmj.com/site/about/guidelines.xhtml

delivered online via BIP is acceptable to participants and shows promise in reducing tics. Furthermore, the study reported no severe adverse events. There were no drop-outs and no data loss for the primary outcome, and 83% of parents and children rated the treatment as good or very good, indicating the trial was highly acceptable to patients in Sweden.

However, the evidence available for the BIP treatment programs has all been gathered in Sweden and we have limited experience of delivering therapist supported online ERP to children and adolescents in the UK. There is evidence that uptake and use of digital health interventions is highly context dependent[21, 22] and it would therefore be unwise to assume that a delivery package that works in Sweden will work equally well in the UK.

The overarching aim of the Online Remote Behavioural Intervention for Tics (ORBIT) trial is to evaluate the clinical and cost-effectiveness of BIP TIC, a therapist-guided, parent-assisted ERP programme for tics in young people with TS in the UK.

Specific objectives are to:

1. To optimise the design and delivery of BIP TIC in partnership with young people with TS, carers and healthcare professionals (HCPs) to maximise acceptability, effectiveness and long-term uptake.
2. To undertake an internal pilot to assess whether recruitment, engagement with the intervention and retention to the trial outcomes are sufficient to allow the trial to progress and provide a definitive answer on effectiveness.
3. To determine the clinical effectiveness of BIP TIC compared to online tic-related education in reducing tics, as measured by the masked assessor-rated Yale Global Tic Severity Scale (YGTSS) total tic severity score (primary outcome).
4. To evaluate the cost-effectiveness of BIP TIC.
5. To estimate the longer-term impact on patient outcomes and health service costs.

For peer review only - http://bmjopen.bmj.com/site/about/guidelines.xhtml

6. To conduct a process analysis in line with MRC guidelines for complex interventions to determine barriers and facilitators to implementation.

The primary hypothesis is that the BIP TIC therapist-guided remote digital ERP intervention will be superior to a comparator intervention of therapist-guided online tic-related education in reducing tics, as measured by the masked assessor-rated YGTSS total tic severity score[23].

For peer review only

METHODS AND ANALYSIS

Trial design

The design is a 10-week, parallel-group, single-blind, non-commercial, randomised controlled superiority trial with an internal pilot. Participants will be randomised to receive 10-weeks of online, remotely-delivered, therapist-supported ERP for tics, or online, remotely-delivered, therapist-supported education for tics. Participants will be followed-up mid-treatment, and at 3-, 6-, 12-, and 18-months post-randomisation. Up until the 6-month follow-up participants are encouraged not to change medication or start alternative therapies for tics. Months 12 and 18 are naturalistic follow-up points where participants are free to access any treatments in accordance with standard practice recommended by their usual treating clinician. A sub-sample of participants and parents will be purposively selected to participate in process evaluation interviews after the 3-month follow-up. Interviews will also be conducted with therapists, clinicians recruiting to the study and commissioners. The process evaluation will also link data on usage of the intervention with outcomes. A schematic diagram of the study design is shown in Figure 1 and a more detailed participant flow is shown in Figure 2.

For peer review only - http://bmjopen.bmj.com/site/about/guidelines.xhtml

An internal pilot will be conducted until the end of the 9th month to determine whether recruitment, engagement with the intervention and retention to the trial are sufficient to allow the trial to progress, the stop/go rules for proceeding are:

1) The study needs to have recruited 30% of the sample (66 participants).
2) At least 60% of participants need to have completed the intervention (defined as the child/adolescent completing at least their first four chapters).
3) 80% of participants who have reached the relevant time window need to have completed the primary outcome measure.

The trial was prospectively registered with ISRTCN (ISRCTN70758207) and ClinicalTrials.gov (NCT03483493).

<<INSERT FIGURE 1>>>

Setting

The intervention will be remotely delivered from two national centres for treating tics in the U.K. (Queen's Medical Centre, Nottingham and Great Ormond Street Hospital, London), serving geographically and demographically diverse populations. However, multiple Patient Identification Centres (PICs) will be involved in recruiting participants into the two research sites.

Recruitment and eligibility

Participant identification

Participants will be identified and recruited by either: (1) clinic staff or clinical study officers (CSOs) at Patient Identification Centres (PICs) across National Health Services (NHS) Trusts in England; or (2) the two study sites (Nottingham and London) or (3) via Tourettes Action

For peer review only

(tic disorder charity) website, our study website, or social media, based on the following criteria:

Inclusion criteria

1) Aged 9 to 17 years-old.
2) Suspected or confirmed Tourette syndrome or chronic tic disorder.
   - Including moderate/ severe tics: Total Tic Severity Score (TTTS) >15 on the YGTSS; TTSS score>10 if motor or vocal tics only.
3) Competent to provide written, informed consent (parental consent for child aged <16 years).
4) Broadband internet access and regular PC/laptop/Mac user, with mobile phone SMS.

Exclusion criteria

1) Receipt of/engaged in structured behavioural intervention for tics (e.g. HRT/CBIT or ERP) within the last 12 months.
2) Change to medication for tics (start or stop) within the previous 2 months.
3) Diagnoses of alcohol/substance dependence, psychosis, suicidality, or anorexia nervosa.
4) Moderate/severe intellectual disability.
5) Immediate risk to self or others.
6) Parent or child not able to speak or read/write English.

Patients will be provided with a brief information sheet and a 'consent to contact' (C2C) form: for NHS referred patients, these forms will be given by a member of the usual care team (or CSO); for self-recruited patients, these will be completed online.

Initial screening

For peer review only

For peer review only - http://bmjopen.bmj.com/site/about/guidelines.xhtml

Upon receipt of a C2C, a member of the research team will undertake a telephone screening interview with the potential participant to determine basic eligibility. Potential participants who meet the initial eligibility requirements will be invited to attend a face-to-face screening/baseline appointment held at one of the two study sites. All participants will be reimbursed for their travel costs to attend this appointment.

Before participants attend the face-to-face screening/baseline appointment parents will be asked to complete an online parent and teacher Development and Wellbeing Assessment (DAWBA)[24] (see secondary outcomes for further details). Completion of the parent DAWBA is a requirement for enrolment into the trial; findings from the DAWBA will be used to exclude people who score highly on self-harm, psychosis, anorexia nervosa or suicidality. It is the parent's decision to involve the teacher in completing the teacher DAWBA.

Face-to-face screening appointment

Potential participants found eligible from the initial telephone screening will be invited to the face-to-face screening/baseline appointment. Consent will be taken at this appointment by a researcher/assessor trained in Good Clinical Practice (GCP), further details about which are listed under 'ethics and dissemination'. Participants will be provided with information sheets which have been developed with our patient and public involvement (PPI) group. Families will be encouraged to ask the research team any questions. Where the participant is a child (under 16-years-old) an age appropriate participant information sheet will be provided.

A trained assessor will conduct the YGTSS[23] assessment to determine the presence of tics (inclusion criteria) and the Child and Adolescent Intellectual Disability Screening Questionnaire (CAIDS-Q)[25] to screen for intellectual disability (exclusion criteria). Details of the YGTSS are described under the primary outcome.

For peer review only - http://bmjopen.bmj.com/site/about/guidelines.xhtml

Patients who are not eligible because they have recently changed their tic medication (within 2-months), or received a behavioural therapy for tics within the specified exclusion time frame (12 months), may be eligible for re-screening at a later date when these time exclusions have passed. Patients who meet the eligibility criteria, after completion of all screening assessments, will be asked to complete baseline measures and will be introduced to the BIP system.

Interventions

The BIP treatment programs are delivered via a secure internet platform that enables presentation of different treatment content to different patient populations. Patients log in via personal user names and passwords to access the treatment content which usually includes text, pictures, instructional videos and worksheets (see link for video of the intervention https://vimeo.com/294763841/628b99ddfb). The treatment content for both groups is presented in chapters, like a self-help book.

Both the ERP (intervention) and the education (active control) are delivered via the BIP platform. The interventions consist of 10 separate child-and parent/carer-chapters which are designed to be delivered over 10 weeks, with access to therapist support during this time.

The child/young person and their parent/carer are provided with their own separate login to the BIP platform to access the relevant child or parent/carer chapters. The first four chapters cover the main content of the treatment; completion of these four child chapters is a minimum requirement to meet treatment completion criteria.

Both children and parents/carers have regular contact with a therapist during the 10 weeks via messages that can be sent inside the treatment platform (resembling an email) or telephone if needed. The therapist can also directly comment on exercises that the participant has been working on, and give specific feedback to motivate the patient. The participant typically has contact with the therapist several times a week, although the contact is asynchronous. The

For peer review only - http://bmjopen.bmj.com/site/about/guidelines.xhtml

therapist is available to support the participants during these 10-weeks, although support may be delivered over a 12-week period if necessary to allow for breaks during holiday and festive periods. Access to the BIP treatment program is granted for 1-year after the 10 weeks of therapist-guided treatment, with the remaining access to the BIP platform being unsupported. The therapist manuals for both interventions were written by the co-applicants.

Therapist guided, internet-delivered ERP Intervention (BIP TIC; experimental group)

The treatment consists of evidence-based interventions adapted from previously published treatment manuals on ERP and established behavioural intervention for tics protocols.[26, 27] During the treatment, participants are instructed to practice suppressing their tics: this is known as 'response prevention'. Then, with the help of their parent/carer, the participant is instructed to provoke premonitory urges and control all of their tics: this is known as 'exposure'. Through treatment, the child gains mastery in tolerating the urge, controlling the tics, and is able to do so for increasingly longer periods of time. The participants also receive detailed education about tics and strategies to promote tic management at school.

The parent modules contain information regarding parent coping strategies, how to support their child in working with BIP and functional analysis relating to tics.

Therapist guided, internet-delivered education (active control group)

An active comparator intervention was chosen to ensure that all participants still received an internet-delivered therapist-guided intervention over-and-above what would typically be received in standard care. Education about tics (without a specific focus on training behavioural strategies to reduce tics) was chosen to enable a comparison to previous trials of face-to-face behaviour therapy for tics.[28]

For peer review only

The comparator intervention was developed for the trial and consisted of educational information about TS and co-occurring conditions. The material was adapted and updated from the educational intervention used in two previous randomised controlled trials.[26, 28]

The chapters have been matched in length to that of the behavioural intervention. The comparator intervention reviews the definition of tics, natural history, common presentations, prevalence, aetiology, risks and protective factors and strategies for describing tics to other people etc. Problem-solving and development of expertise in tic disorders is emphasised. The parent modules emphasise parent coping strategies and self-care in caring for a child with tic disorder. There is no information on tic control within the comparator intervention.

Start of treatment

The participant will agree a start date for commencing therapy with their assessor at the screening/baseline appointment (typically within 24-48 hours of the baseline appointment). The participant will be reminded that it is important to log-on and start the therapy on the allocated date. At the start, the therapist will release the first two chapters of the intervention for the participants to complete and monitor their progress, sending reminders and feedback where necessary. If the participant does not start the intervention at the agreed start time, the 10-week start date does not alter.

All therapists will undergo training delivered by a supervisor (TM or JK) before commencing either intervention. Therapists will be closely supervised by TM or JK to ensure adherence to protocol. The team from the Karolinska Institutet will provide technical support throughout the trial.

Measures and outcomes

Primary outcome

For peer review only

The primary outcome is the severity of tics as measured by the TTSS (0-50) on the YGTSS .[23] The primary end point is 3-months post-randomisation. The TTSS combines separate scales for motor tics (0-25) and for vocal tics (0-25). The primary outcome measure (TTSS) will be measured at baseline (pre-intervention), 3-months, 6, 12 and 18-months post randomisation.

The YGTSS is administered by a blinded assessor as an investigator-based semi-structured interview focussing on motor and vocal tic frequency, severity and tic related impairment over the previous week. The baseline assessment will be conducted face-to-face at the baseline/screening appointment. When possible, the follow-up assessments will be conducted by the same blinded assessor that conducted the baseline assessment, via video-conference call/WebEx or telephone. With the participants' permission, all YGTSS assessments will be recorded for quality checks.

All assessors/researchers will be trained in conducting YGTSS by a clinical expert (TM). Agreement with the expert rater will be established at the start of the trial and monitored every six-months. Assessors have to be within 15% of the expert rater (TM) for the Total Motor Tic score, the Total Vocal Tic score and the Total Tic Score on 3 YGTSS recordings at baseline and 4 randomly selected recordings at follow-up. Assessors who do not meet this threshold of agreement will be re-trained.

Secondary outcomes

A flowchart of study assessments is shown in Table 1 which documents the time point for completion and the informant. See also Figure 2.

Table 1. Schedule of assessments

For peer review only - http://bmjopen.bmj.com/site/about/guidelines.xhtml

**Months post-randomisation**	**0**	**0**	**1**	**1**	**3**	**6**	**12**	**18**	**Completed by**
**Time Point**	Telephone screen	Baseline assessment	Mid-treatment (3 weeks)	Mid-treatment (5 weeks)	Primary end point	6-months	12-months	18-months	
Screening for eligibility	X	X							R
SDQ & DAWBA (conducted post telephone screen & prior to baseline)	X								P & T
YGTSS TTSS		X			X	X	X	X	R
YGTSS Impairment		X			X	X	X	X	R
CAIDS-Q		X							P/R
SCQ		X							P
SNAP-IV		X							P
Demographics		X							R
PUTS		X							C
PTQ		X		X	X	X	X	X	P
CGAS		X			X	X	X	X	R
CHU9D		X			X	X	X	X	P
Modified CSRI (inc school attendance)		X			X	X	X	X	P/R
MFQ-(Self-report)		X		X	X	X	X	X	C
SCAS (Self-report)		X			X	X	X	X	C
C&A GTS-QOL		X			X	X	X	X	C
Adverse effects/side effects		X		X	X	X			P/C
Concomitant interventions		X			X	X	X	X	P/R
SDQ (P)					X	X	X	X	P
CGI-I					X	X	X	X	R

For peer review only

Treatment credibility			X						P/C
Treatment satisfaction					X				P/C
Need for further treatment					X				P/C
Interview (Process evaluation)					X				

C = child; C&A-GTS-QOL = Child and adolescent version of the Gilles de la Tourette Syndrome Quality of Life Scale; CAIDS-Q = Child and Adolescent Intellectual Disability Screening Questionnaire; CGAS = Children's Global Assessment Scale; CGI-I = Clinical Global Impressions- Improvement; CHU9D = Child Health Utility 9D; CSRI = Client Service Receipt Inventory; DAWBA = Development and Wellbeing Assessment; MFQ = Mood and Feelings Questionnaire; P = parent; PTQ = Parent Tic Questionnaire; PUTS = Premonitory Urges for Tics Scale; R = researcher; SCAS = Spence Child Anxiety Scale; SCQ = Social Communication Questionnaire; SDQ = Strengths and Difficulties Questionnaire; SNAP-IV; Swanson, Nolan and Pelham Rating Scale; T = teacher; TTSS= Total Tic Severity Scale; YGTSS = Yale Global Tic Severity Scale.

<<INSERT FIGURE 2>>>

- *YGTSS – Impairment scale*:[23] The impairment scale (0-50) forms part of the YGTSS described above. The rating focuses on distress and impairment experienced in interpersonal, academic, and occupational domains.
- *Parent Tic Questionnaire (PTQ)*:[29] The PTQ assesses the number, frequency, and intensity of motor and vocal tics.
- *Clinical Global Impressions Scale (CGI)-Improvement*:[30] The CGI Improvement provides an overall clinician-determined summary measure of symptom improvement.
- *Children's Global Assessment Scale (CGAS)*:[31] The CGAS is a 0-100 scale that integrates psychological, social and academic functioning in children as a measure of psychiatric disturbance.

For peer review only

For peer review only - http://bmjopen.bmj.com/site/about/guidelines.xhtml

- *Strengths and Difficulties Questionnaire (SDQ – parent completed)*:[32] The SDQ is a brief measure of behavioural and emotional difficulties. The SDQ will be completed by parents as part of DAWBA at baseline and at follow-up via an online platform.
- *The Mood and Feelings Questionnaire (MFQ; Child completed version)*: [33] The MFQ reports depressive symptoms as specified by the DSM-III-R diagnostic criteria for major depression.
- *Spence Child Anxiety Scale (SCAS - self-report)*:[34] The SCAS is a self-report measure that evaluates symptoms relating to separation anxiety, social phobia, obsessive-compulsive disorder, pain, agoraphobia, generalised anxiety and fears of physical injury.
- *Child Health Utility 9D (CHU9D; parent and child completed versions)*: [35] The CHU9D is a paediatric quality of life measure for use in health care resource-allocation decision making.
- *The Child and Adolescent Gilles de la Tourette Syndrome–Quality of Life Scale (C&A-GTS-QOL)*:[36] The C&A-GTS-QOL is a disease-specific measure of health-related quality of life designed for children and adolescents with TS.
- *Client Service Receipt Interview (CSRI)*:[37] The CSRI is a flexible research instrument developed to collect information on service receipt, service-related issues and income. A modified version of the CSRI will be completed which also combines elements of the Child and Adolescent Service Use Schedule (CA-SUS).[38]
- *Adverse events (Side effects)*: Adverse events/side effects will be recorded on a modified version of the side effects scale developed by Hill and Taylor.[39]

For peer review only - http://bmjopen.bmj.com/site/about/guidelines.xhtml

The above mentioned scales all have established reliability, validity and history of use in clinical and research settings. Some further measures were developed by the research team for the purpose of this study, these include:

- *Treatment credibility:* The questionnaire consists of 2-items which ask how suitable the child and parent believe the internet treatment is for managing tics, and how much improvement they expect from the treatment.
- *Treatment satisfaction and need for further treatment:* 7-items ask about the child's/parent's satisfaction with the treatment and an additional question asks whether they feel more tic treatment is required.
- *Concomitant interventions:* To assess what other treatments/interventions the child/young person is accessing during the study period, parents will be asked to complete a short questionnaire documenting other interventions (including medication) in progress.

Additional baseline measures

To further characterise the sample at baseline, the following measures will also be recorded:

- *Demographics questionnaire*: Records the child's age, gender, ethnicity, parental education/ occupation, list of the child's current diagnoses and interventions (including medications) and general practitioner (GP) and school details.
- *Social Communication Questionnaire (SCQ)*:[40] The SCQ is a screening tool for Autism Spectrum Disorder (ASD).
- *Premonitory Urge for Tics Scale (PUTS)*:[41] The PUTS is a self-report instrument that measures premonitory urges in tic disorders.

For peer review only

- *Swanson, Nolan, and Pelham Rating Scale (SNAP-IV)*:[42] The SNAP-IV is a behavioural rating scale that includes the core symptoms of ADHD and oppositional defiant disorder (ODD).

Process evaluation

The process evaluation will follow the Medical Research Council (MRC) guidelines for evaluating the implementation of complex interventions[43 44]. It will explore the fidelity and implementation of intervention, and make recommendations for adaptations. It will also examine the potential mechanisms underlying participant behaviour change and probe for unexpected consequences.

Interviews will be conducted with therapists and their supervisors supporting the intervention (target n>5), clinicians recruiting to the study (n = >10), children (n=>20 in each arm) and parents of children in both arms (n=>20 in each arm). Online feedback from participants will also be analysed together with indices recorded as part of the BIP system such as total therapist time, number of chapters clicked through and number of patient log-ins.

Patient and public involvement (PPI)

A group of PPI members was created to shape and guide the study. The group is comprised of four parents and four children and young people with a diagnosis of Tourette syndrome, plus two PPI members with previous experience of large studies. The design of the trial and its dissemination will also have been shaped by PPI in the following ways:

- reviewing and selection of outcome measures
- co-development of study materials and recruitment processes
- reviewing the interventions and providing feedback on all chapters

For peer review only - http://bmjopen.bmj.com/site/about/guidelines.xhtml

- attending ongoing trial team meetings to provide PPI perspective and assist with troubleshooting (e.g. recruitment and retention)
- guiding interview topics and shaping questions for process evaluation,
- co-creation of lay summaries and other dissemination materials

The PPI group mainly contributes remotely, in order to enable involvement from members who are not geographically close. Face-to-face input occurs at key points, for example attendance at trial team meetings.

Sample size

To detect a clinically important average difference of 0.5 of a standard deviation between intervention and comparator with 90% power at $p<0.05$ (2-sided), after allowing for 20% dropout, requires a total sample size of 220 participants. A systematic review found the average estimate for the standard deviation (SD) of the YGTSS (TTSS) from 19 trials of behavioural intervention for tics was 6.6.[17] Thus, the trial is powered to detect an average difference of 3.3 on the YGTSS.

Recruitment is planned to be conducted within an 18-month period. We are not aware of any other competing studies that are in progress or planned that would affect recruitment. Input received from young people and carers in developing this trial indicates that participation in the trial will be attractive as it guarantees access to an intervention. Our national recruitment network of specialist (PIC) clinics, including multiple community child and adolescent mental health services (CAMHS) and paediatric clinics and the charity Tourettes Action, have the capacity to meet the recruitment target within the time window. Recruitment figures will be monitored monthly by the Trial Manager (CLH) and any shortfall will be reported to the project management group (PMG).

For peer review only - http://bmjopen.bmj.com/site/about/guidelines.xhtml

Randomisation

Randomisation will be conducted using the Sealed Envelope online randomisation system and managed by Priment Clinical Trials Unit (CTU). Participants will be randomised online by the researcher/assessor. Randomisations will be distributed equally between the two arms (ratio 1:1) and stratified by study site using block randomisation with varying block sizes. The researcher will remain blind to the treatment allocation, and the therapist will be notified of the treatment allocation. Therapists will set up participants' treatment on the BIP system.

All participants receive the same outcome measures so assessors will not know which group the participant is in by the measurements. If an assessor becomes unblinded, subsequent assessments for that participant will be conducted by a different assessor (blind to arm allocation) where possible. In case of a medical emergency, participants will be able to disclose to the treating physician (e.g. GP) what treatment they received without unblinding the researchers. Outcome assessors will guess the treatment allocation after each assessment, to be able to calculate if the guesses are better than random.

Data management and analysis

Participants who do not complete therapy or measures at a given time point will still be followed up until the end of the trial unless they inform the research team they would like to withdraw from the trial. Participants will be reimbursed with £20 vouchers for completing measures at baseline and each follow-up time point, except the mid-treatment follow-ups (3 and 5 weeks).

All aspects of data management of the study will comply with the UK Data Protection Act 1998/ General Data Protection Regulation (GDPR), Priment's standard operating procedures (SOPs) and GCP. Outcome measures will be stored in BASS, a rating platform stored on a Swedish server. Participant completed outcome measures are directly entered into an online

For peer review only - http://bmjopen.bmj.com/site/about/guidelines.xhtml

database by the participant. Assessor/researcher completed measures may initially be entered on a paper case report form (CRF) or directly into the database. Data extraction will be performed within the UK.

Statistical analysis

All baseline variables will be summarised by randomised group. Categorical data will be reported as frequencies (%) and continuous data will be reported as mean (SD) unless skewed then they will be reported as median (interquartile range). No statistical tests will be carried out comparing baseline variables. The primary outcome (total tic severity score [TTSS] of the YGTSS at 3-months post-randomisation) will be analysed using a linear regression model, including baseline TTSS and centre (the stratification variable). If the assumptions of linear regression are violated, another suitable method will be used. Primary analyses will be performed using intention to treat principles. Predictors of missingness will be examined. If any are found, they will be included in statistical models in a supportive analysis. There are no planned subgroup analyses.

Secondary outcomes will be analysed using similar statistical methods as the primary outcome. Continuous outcomes will be analysed with linear regression. Dichotomous outcomes will be analysed using logistic regression. Results will be presented as estimated differences or odds ratios as appropriate, with accompanying 95% CI. P values will not be reported for secondary outcomes.

Economic analysis

The primary cost-effectiveness analysis for the within-trial evaluation will be the mean incremental cost per point change in YGTSS of treatment as usual (TAU) plus BIP TIC compared to TAU plus online education with therapist support at 3 months, from an NHS and personal social services cost perspective.

For peer review only - http://bmjopen.bmj.com/site/about/guidelines.xhtml

A secondary analysis of the mean incremental cost per quality-adjusted life-year (QALY) of the intervention compared to the active control from a health and social care cost perspective will also be calculated. A decision model projecting costs and QALYs into adulthood will also be developed.

Qualitative analysis

Interview data collected from the process evaluation will be analysed using thematic analysis.[45] Data will be analysed using NVivo to establish themes and subthemes. A mixed-methods approach will be used to integrate quantitative and qualitative data in order to fully explore implementation of the intervention.

Monitoring

Management and oversight

There are three committees in place to oversee the trial, including: a Project Management Group (PMG), Trial Steering Committee (TSC) and Data Monitoring and Ethics Committee (DMEC). The PMG consists of all the co-investigators listed on the protocol who will meet at least every six months to discuss the study progress and overall conduct of the trial. The role of the TSC will be to provide overall supervision for the trial on behalf of the Trial Sponsor and Trial Funder, to ensure that the trial is conducted in accordance with GCP, and to review the findings of the internal pilot. The DMEC is the only body involved in the trial that has access to the unblinded comparative data. The role of its members is to monitor these data and make recommendations to the TSC on whether there any ethical or safety reasons why the trial should not continue. There are no interim analyses planned, however this does not preclude the DMEC from requesting analyses. The TSC and DMEC will meet annually. The charters are available on request from the corresponding author.

For peer review only - http://bmjopen.bmj.com/site/about/guidelines.xhtml

Adverse events

There are no anticipated serious adverse events. All adverse events will be recorded and monitored and the Chief Investigator and medical expert (CH) will determine seriousness and causality and report the event to the DMEC and ethics committee where necessary. Side effects will be formally monitored via completion of the side-effects scale throughout the intervention and for 3-months after the intervention finishes (month 6).

Audit

The Trial Manager, or a nominated designee of the Sponsor, shall carry out monitoring of trial data as an ongoing activity. The Sponsor may decide to carry out a full audit of the trial and the trial management systems/ procedures in the event of serious breaches to GCP or protocol. Trial data and evidence of monitoring and system audits will be made available for inspection by the Research Ethics Committee (REC) as required.

ETHICS AND DISSEMINATION

The study received ethical and Health Research Authority (HRA) approval from North West Greater Manchester Research Ethics Committee on 23rd March 2018 (Ref: 18/NW/0079: protocol version 2.0, 26th February 2018). Local approvals have been granted from the participating Trusts. The study is sponsored by Nottinghamshire Healthcare NHS Foundation Trust; neither the sponsor nor the funders will be involved in the analysis of study data or report writing. Only the UK research team will have access to the study data, which will be stored in secure locked files or password protected databases. Data will be available for inspection by the ethics committee upon request. Changes to the protocol will be communicated to the ethics committee, funders, and trial registries by the trial manager

For peer review only - http://bmjopen.bmj.com/site/about/guidelines.xhtml

(CLH). The process for obtaining participant informed consent or assent and parent/ guardian informed consent will be in accordance with the ethical guidance and GCP. The investigator or their nominee and the participant or other legally authorised representative (such as the child's parent) shall both sign and date informed consent forms before the person can participate in the study. Where the young person is 16-years and over, written consent will be required from both the parent (Supplementary File A) and young person (Supplementary File B). Where the young person is under 16-years, written parental consent will be required, alongside the young person's assent (Supplementary File C). In the event of any conflict between the parent and child, the child will not enter the study.

Individual participant medical information obtained as a result of this study is considered confidential and disclosure to third parties is prohibited unless warranted by an adverse event. Participant confidentiality will be further ensured by utilising identification numbers to correspond to treatment data in the computer files. No post-trial care is required; however, participants will have access to the intervention for one-year after starting the treatment, although this access will be without therapist support after the 10/12weeks. The Chief Investigator and site principal investigators declare no financial or competing interests. At the end of the trial, the data belongs to the Chief Investigator and the study sponsor. All members of the team will have access to the final trial dataset. The findings will be published in peer-reviewed journals, presented at relevant conferences and disseminated to the public via lay summaries co-created with our PPI group. All outputs will be authored by the research team and will not involve professional writers. Access to the full protocol is available upon request to the corresponding author.

Acknowledgements: ORBIT Trial team members:, Robin Carpenter, Caitlin Hand, Anne Marie Downey, Kareem Khan, Natalia Lago, Jennifer L Martin, and Roz Shafran.

For peer review only - http://bmjopen.bmj.com/site/about/guidelines.xhtml

We would like to thank Tourettes Action for their support with the trial, we particularly acknowledge Dr Seonaid Anderson for her ongoing help and advice. We would like to thank our Patient and Public Involvement (PPI) members for their help, including: Debbie Butler, Julie Bryant, James Bungay, Claire Bungay, Sandra Wang, Marco Wang, Alison Neave, Matthew Neave, Rachel Jopling, and Luke Jopling. We thank members of our Trial Steering Committee: Prof Edmund Sonuga-Barke (Chair), Mrs Neelam Gurav, Dr Prathiba Chisabesan, Dr Kim Goldsmith, Dr David Manley, and Dr Cara Verdellen. We also thank members of our Data Safety and Monitoring Committee: Prof Stephen Scott (Chair), Dr Uttom Chowdhury, and Prof Richard Emsley. Finally, we would like to thank Angela Summerfield for her continued support and help.

Author contributions: CH is the Chief Investigator and takes final responsibility for study design, conduct and decision to submit for publication. The study design was conceived and written by CH, CLH (Trial Manager), PA, TM, DMC, EM, LM, MPC, IH, ES, RJ, RH, and CG (co-investigators). CLH wrote the protocol manuscript with approval from all authors. LM and RJ (statisticians) designed and wrote the statistical analysis plan. RH (health economist and co-investigator) designed and wrote the health economic plan. CG leads on the process evaluation. EBD (therapist) and TM and JK lead on delivery and design of the therapy. SuB (co-investigator) leads on PPI. JK, MPC, IH, SoB and CS and BJB (researchers) and AE and LC (therapists) provided advice and critical input on the study design. All authors critically revised the manuscript for its important intellectual content. All authors read and approved the final manuscript.

Funding statement: This research was funded by the NIHR Heath Technology Assessment (HTA) (Ref 16/19/02). The views expressed are those of the author(s) and not necessarily those of the NHS, the NIHR or the Department of Health and social care. MPC, SB, EBD, CG and CH acknowledge the financial support of the NIHR Nottingham Biomedical

For peer review only - http://bmjopen.bmj.com/site/about/guidelines.xhtml

Research Centre and NIHR MindTech MedTech Co-operative. The study sponsor and funders have no role in study design, including; collection, management, analysis and interpretation of data; writing of the report; and the decision to submit the report for publication.

Trial Sponsor: Nottinghamshire Healthcare NHS Foundation Trust; Shirley Mitchell. Duncan Macmillan House, Porchester Road, Mapperley, Nottingham, UK, NG3 6AA Shirley.mitchell@nottshc.nhs.uk (Ref: Hollis201117).

Competing interests: On behalf of all authors, the corresponding author declares no competing interests.

Ethical approval: The initial protocol (version 2.0, 26th February 2018) was approved by North West Greater Manchester Research Ethics Committee (REC reference 18/NW/0079) on 23rd March 2018. A subsequent amendment (version 3.0, 16th April 2018) was approved by the committee on 15 May 2018 to allow the 10-week intervention to be delivered over a 12-week period to account for periods of therapist absence during bank holidays or unforeseen circumstances.

References

1. Hollis C, Pennant M, Cuenca J, et al. Clinical effectiveness and patient perspectives of different treatment strategies for tics in children and adolescents with Tourette syndrome: a systematic review and qualitative analysis. *Health Technol Asses* 2016;20(4):1-450. DOI: 10.3310/hta20040

2. Freeman RD, Fast DK, Burd L, et al. An international perspective on Tourette syndrome: selected findings from 3500 individuals in 22 countries. *Dev Med Child Neurol* 2000;42(7):436-47. DOI: 10.1111/j.1469-8749.2000.tb00346.x

For peer review only - http://bmjopen.bmj.com/site/about/guidelines.xhtml

3. Verdellen C, Van De Griendt J, Hartmann A, et al. European clinical guidelines for Tourette syndrome and other tic disorders. Part III: behavioural and psychosocial interventions. *Eur Child Adoles Psy* 2011;20(4):197-207. DOI: 10.1007/s00787-011-0167-3

4. Whittington C, Pennant M, Kendall T, et al. Practitioner Review: Treatments for Tourette syndrome in children and young people–a systematic review. *J Child Psychol Psyc* 2016;57(9):988-1004. DOI: 10.1111/jcpp.12556

5. Roessner V, Plessen KJ, Rothenberger A, et al. European clinical guidelines for Tourette syndrome and other tic disorders. Part II: pharmacological treatment. *Eur Child Adoles Psy* 2011;20(4):173-96. DOI: 10.1007/s00787-011-0163-7

6. Cuenca J, Glazebrook C, Kendall T, et al. Perceptions of treatment for tics among young people with Tourette syndrome and their parents: a mixed methods study. *BMC Psychiatry* 2015;15(1):46. DOI: 10.1186/s12888-015-0430-0

7. Verdellen CW, Keijsers GP, Cath DC, et al. Exposure with response prevention versus habit reversal in Tourettes's syndrome: a controlled study. *Behav Res Ther* 2004;42(5):501-11. DOI: 10.1016/S0005-7967(03)00154-2

8. Rizzo R, Pellico A, Silvestri PR, et al. A Randomized Controlled Trial Comparing Behavioral, Educational, and Pharmacological Treatments in Youths With Chronic Tic Disorder or Tourette Syndrome. *Front Psych* 2018;9:100. DOI: 10.3389/fpsyt.2018.00100

9. Andersson G, Cuijpers P, Carlbring P, et al. Guided Internet-based vs. face-to-face cognitive behavior therapy for psychiatric and somatic disorders: a systematic review and meta-analysis. *World Psychiatry* 2014;13(3):288-95. DOI: 10.1002/wps.20151

For peer review only

For peer review only - http://bmjopen.bmj.com/site/about/guidelines.xhtml

10. Hedman E, Ljótsson B, Lindefors N. Cognitive behavior therapy via the Internet: a systematic review of applications, clinical efficacy and cost–effectiveness. *Expert Rev Pharm Out* 2012;12(6):745-64. DOI: 10.1586/erp.12.67

11. Palmqvist B, Carlbring P, Andersson G. Internet-delivered treatments with or without therapist input: does the therapist factor have implications for efficacy and cost? *Expert Rev Pharm Out* 2007;7(3):291-97. DOI: 10.1586/14737167.7.3.291

12. Johansson R, Andersson G. Internet-based psychological treatments for depression. *Expert Rev Neurother* 2012;12(7):861-70. DOI: 10.1586/ern.12.63

13. Cuijpers P, Donker T, Johansson R, et al. Self-guided psychological treatment for depressive symptoms: a meta-analysis. *PLoS One* 2011;6(6):e21274. DOI: 10.1371/journal.pone.0021274

14. Vigerland S, Thulin U, Ljótsson B, et al. Internet-delivered CBT for children with specific phobia: a pilot study. *Cogn Behav Therapy* 2013;42(4):303-14. DOI: 10.1016/j.brat.2015.11.006

15. Vigerland S, Ljótsson B, Thulin U, et al. Internet-delivered cognitive behavioural therapy for children with anxiety disorders: A randomised controlled trial. *Behav Res Ther* 2016;76:47-56. DOI: 10.1016/j.brat.2015.11.006

16. Lenhard F, Vigerland S, Andersson E, et al. Internet-delivered cognitive behavior therapy for adolescents with obsessive-compulsive disorder: an open trial. *PLoS One* 2014;9(6):e100773. DOI: 10.1371/journal.pone.0100773

17. Hollis C, Falconer CJ, Martin JL, et al. Annual Research Review: Digital health interventions for children and young people with mental health problems–a systematic and meta-review. *J Child Psychol Psyc* 2017;58(4):474-503. DOI: 10.1111/jcpp.12663

For peer review only - http://bmjopen.bmj.com/site/about/guidelines.xhtml

18. Himle MB, Freitag M, Walther M, et al. A randomized pilot trial comparing videoconference versus face-to-face delivery of behavior therapy for childhood tic disorders. *Behav Res Ther* 2012;50(9):565-70. DOI: 10.1016/j.brat.2012.05.009

19. Ricketts EJ, Goetz AR, Capriotti MR, et al. A randomized waitlist-controlled pilot trial of voice over Internet protocol-delivered behavior therapy for youth with chronic tic disorders. *J Telemed Telecare* 2016;22(3):153-62. DOI: 10.1177/1357633X15593192

20. Andrén P, Aspvall K, Fernández de la Cruz L, et al. Therapist- and parent-guided Internet-delivered behaviour therapy for paediatric Tourette's Disorder: a pilot randomised controlled trial with long-term follow-up. *PsyArXiv (web)* October 21st 2018. DOI:10.31234/osf.io/dp3qz

21. Lau R, Stevenson F, Ong BN, et al. Achieving change in primary care—causes of the evidence to practice gap: systematic reviews of reviews. *Implement Sci* 2016;11(1):40. DOI: 10.1186/s13012-016-0396-4

22. Kaplan HC, Brady PW, Dritz MC, et al. The influence of context on quality improvement success in health care: a systematic review of the literature. *Milbank Q* 2010;88(4):500-59. DOI: 10.1111/j.1468-0009.2010.00611.x

23. Leckman JF, Riddle MA, Hardin MT, et al. The Yale Global Tic Severity Scale: initial testing of a clinician-rated scale of tic severity. *J Am Acad Child Psy* 1989;28(4):566-73. DOI: 10.1097/00004583-198907000-00015

24. Goodman R, Ford T, Richards H, et al. The Development and Well-Being Assessment: description and initial validation of an integrated assessment of child and adolescent psychopathology. *Journal of Child Psychology and Psychiatry* 2000;41(05):645-55.

25. McKenzie K, Murray GC, Murray AL. The Validity of the Child and Adolescent Intellectual Disability Screening Questionnaire (CAIDS-Q) with children aged 6–7

For peer review only - http://bmjopen.bmj.com/site/about/guidelines.xhtml

years, 11 months: A brief report. *Psychiat Res* 2013;210(2):675-77. DOI: 10.1016/j.psychres.2013.06.014

26. Yates R, Edwards K, King J, et al. Habit reversal training and educational group treatments for children with tourette syndrome: a preliminary randomised controlled trial. *Behav Res Ther* 2016;80:43-50. DOI: 10.1016/j.brat.2016.03.003

27. Verdellen C, Van de Griendt J, Kriens S, et al. Tics - Therapist Manual [program]. Amsterdam, Netherlands: Boom Publishers, 2011.

28. Piacentini J, Woods DW, Scahill L, et al. Behavior therapy for children with Tourette disorder: a randomized controlled trial. *JAMA* 2010;303(19):1929-37. DOI: 10.1001/jama.2010.607

29. Chang S, Himle MB, Tucker BT, et al. Initial psychometric properties of a brief parent-report instrument for assessing tic severity in children with chronic tic disorders. *Child Fam Behav Ther* 2009;31(3):181-91. DOI: 10.1080/07317100903099100

30. Guy W. ECDEU assessment manual for psychopharmacology. *US Department of Health, and Welfare* 1976:534-37.

31. Shaffer D, Gould MS, Brasic J, et al. A children's global assessment scale (CGAS). *Arch Gen Psychiat* 1983;40(11):1228-31. DOI: 10.1001/archpsyc.1983.01790100074010

32. Goodman R. The extended version of the Strengths and Difficulties Questionnaire as a guide to child psychiatric caseness and consequent burden. *Journal of Child Psychology and Psychiatry* 1999;40(5):791-99.

33. Angold A, Costello EJ, Messer SC, et al. Development of a short questionnaire for use in epidemiological studies of depression in children and adolescents. *Int J Meth Psych Res* 1995; *5*(4), 237-249.

34. Spence SH. A measure of anxiety symptoms among children. *Behav Res Ther* 1998;36(5):545-66. DOI: 10.1016/S0005-7967(98)00034-5

For peer review only - http://bmjopen.bmj.com/site/about/guidelines.xhtml

35. Stevens K. The Child Health Utility 9D (CHU9D). *A new, paediatric, preference-based measure of health related quality of life PRO Newsletter* 2010;43:11-2.

36. Cavanna AE, Luoni C, Selvini C, et al. Disease-specific quality of life in young patients with Tourette syndrome. *Pediatr Neurol* 2013;48(2):111-14. DOI: 10.1016/j.pediatrneurol.2012.10.006

37. Beecham J, Knapp M. Costing Psychiatric Interventions. In: Thornicroft G, Brewin C, Wing J, eds. Measuring mental health needs. London: Gaskell 2001:200-24.

38. Byford S, Barrett B, Roberts C, et al. Cost-effectiveness of selective serotonin reuptake inhibitors and routine specialist care with and without cognitive–behavioural therapy in adolescents with major depression. *Brit J Psychiat* 2007;191(6):521-27. DOI: 10.1192/bjp.bp.107.038984

39. Hill P, Taylor E. An auditable protocol for treating attention deficit/hyperactivity disorder. *Arch Dis Child* 2001;84(5):404-09. DOI: 10.1136/adc.84.5.404

40. Rutter M, Bailey A, Lord C. Social Communication Questionnaire (SCQ). Los Angeles: Western Psychological Services 2003.

41. Woods DW, Piacentini J, Himle MB, et al. Premonitory Urge for Tics Scale (PUTS): initial psychometric results and examination of the premonitory urge phenomenon in youths with Tic disorders. *J Dev Behav Pediatr* 2005;26(6):397-403.

42. Swanson JM, Kraemer HC, Hinshaw SP, et al. Clinical relevance of the primary findings of the MTA: success rates based on severity of ADHD and ODD symptoms at the end of treatment. *J Am Acad Child Psy* 2001;40(2):168-79. DOI: 10.1097/00004583-200102000-00011

43. Craig P, Dieppe P, Macintyre S, et al. Developing and evaluating complex interventions: the new Medical Research Council guidance. *BMJ* 2008;337:a1655. DOI: https://doi.org/10.1136/bmj.a1655

For peer review only - http://bmjopen.bmj.com/site/about/guidelines.xhtml

44. Moore GF, Audrey S, Barker M, et al. Process evaluation of complex interventions: Medical Research Council guidance. *BMJ* 2015;350. DOI: 10.1136/bmj.h1258

45. Braun V, Clarke V. Using thematic analysis in psychology. *Qual Res Psychol* 2006;3(2):77-101.DOI: 10.1191/1478088706qp063oa

FIGURE CAPTION:

Figure 1: Schematic flow diagram of trial design

Key: CAIDS-Q = Child and Adolescent Intellectual Disability Screening Questionnaire; CTU = Clinical Trials Unit; DAWBA = Development and Wellbeing Assessment; YGTSS = Yale Global Tic Severity Scale.

Figure 2: Participant flow chart

Key: C&A-GTS-QOL = Child and adolescent version of the Gilles de la Tourette Syndrome Quality of Life Scale; CAIDS-Q = Child and Adolescent Intellectual Disability Screening Questionnaire; CGAS = Children's Global Assessment Scale; CGI-I = Clinical Global Impressions- Improvement; CHU9D = Child Health Utility 9D; CSRI = Client Service Receipt Inventory; DAWBA = Development and Wellbeing Assessment; MFQ = Mood and Feelings Questionnaire; PTQ = Parent Tic Questionnaire; PUTS = Premonitory Urge for Tics Scale; RT = Research team; SCAS = Spence Child Anxiety Scale; SCQ = Social Communication Questionnaire; SNAP-IV; Swanson, Nolan and Pelham Rating Scale; SDQ = Strengths and Difficulties Questionnaire; YGTSS = Yale Global Tic Severity Scale.

Note: The CSRI includes a measure of school attendance. The YGTSS includes both a measure of severity and impairment.

For peer review only - http://bmjopen.bmj.com/site/about/guidelines.xhtml

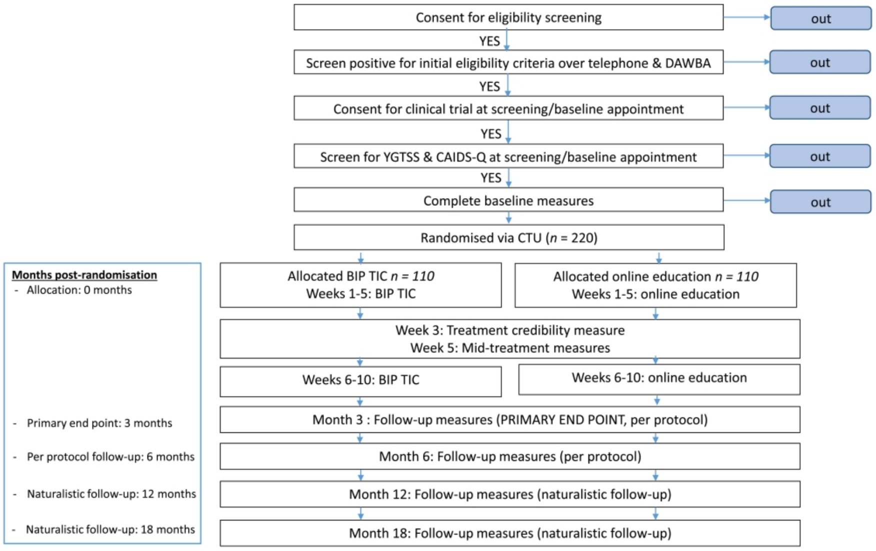

Figure 1

338x190mm (300 x 300 DPI)

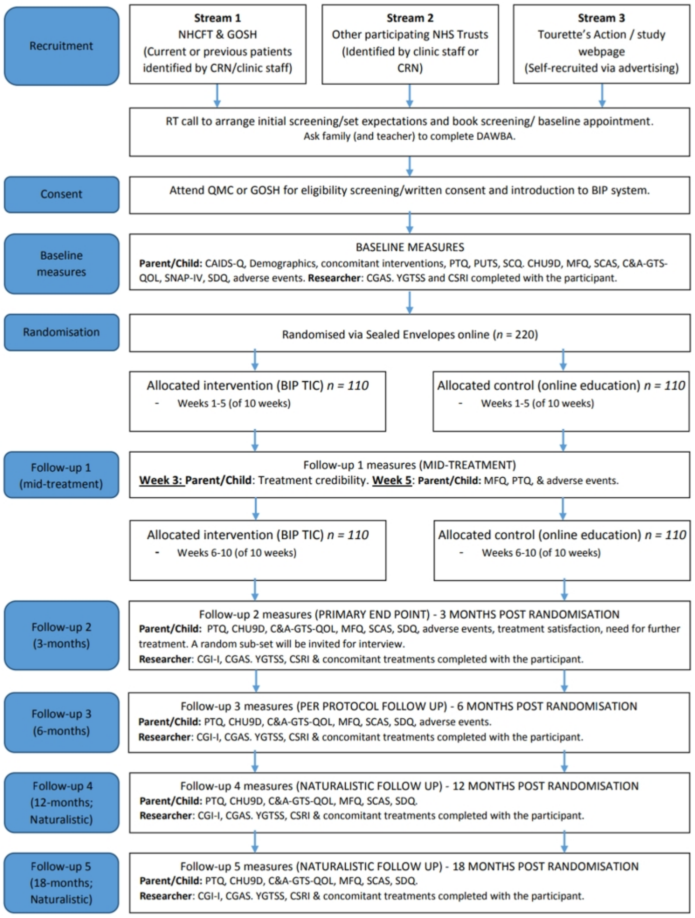

Figure 2

Key: C&A-GTS-QOL = Child and adolescent version of the Gilles de la Tourette Syndrome Quality of Life Scale; CAIDS-Q = Child and Adolescent Intellectual Disability Screening Questionnaire; CGAS = Children's Global Assessment Scale; CGI-I = Clinical Global Impressions- Improvement; CHU9D = Child Health Utility 9D; CSRI = Client Service Receipt Inventory; DAWBA = Development and Wellbeing Assessment; MFQ = Mood and Feelings Questionnaire; PTQ = Parent Tic Questionnaire; PUTS = Premonitory Urge for Tics Scale; RT = Research team; SCAS = Spence Child Anxiety Scale; SCQ = Social Communication Questionnaire; SDQ = Strengths and Difficulties Questionnaire; YGTSS = Yale Global Tic Severity Scale.

Note: The CSRI includes a measure of school attendance. The YGTSS includes both a measure of severity and impairment.

Figure 2

401x669mm (300 x 300 DPI)

Supplementary File A: Consent for parent/carers

CONSENT FORM FOR PARENTS/CARERS

Centre Name: Nottinghamshire Healthcare NHS Foundation Trust

REC reference: 18/NW/0079

Participant Identification Number for this trial:

CONSENT FORM

Title of Project: Online Remote Behavioural Intervention for Tics (ORBIT)

Name of Researcher:

Please initial box

1. I confirm that I have read the information sheet dated ______ (version__) for the above study. I have had the opportunity to consider the information, ask questions and have had these answered satisfactorily. ☐

2. I understand that mine and my child's participation is voluntary and that I am free to withdraw at any time without giving any reason, without my medical care or legal rights being affected. ☐

3. I understand that relevant sections of my child's medical notes and data collected during the study, may be looked at by individuals from the ORBIT team, from regulatory authorities or from the NHS Trust, where it is relevant to my taking part in this research. I give permission for these individuals to have access to my records. I understand this data will be stored in the UK (Sealed Envelopes) and Sweden (BIP and BASS) secure databases and servers. ☐

4. I understand that the information collected about me and my child will be used to support other research in the future, and may be shared anonymously with other researchers. ☐

5. I agree to my child's General Practitioner being informed of our participation in the study. ☐

For peer review only

Supplementary File A: Consent for parent/carers

6. I understand that I/my child may be asked to take part in research interviews, which will be recorded and anonymous direct quotes from these interviews may be used in study reports. ☐

7. I agree for me and my child (named below) to take part in the above study. ☐

Name of Parent/carer	Date	Signature

Name of child

Name of Person taking consent	Date	Signature

When completed: 1 for participant; 1 for researcher site file; 1 (original) to be kept in medical notes.

This research was funded by the NIHR Health Technology Assessment (ref 16/19/02). The views expressed are those of the author(s) and not necessarily those of the NHS, the NIHR or the Department of Health.

Funded by NIHR

For peer review only - http://bmjopen.bmj.com/site/about/guidelines.xhtml

Supplementary File B: Consent form for young people aged 16-years and over

CONSENT FORM FOR YOUNG PEOPLE 16 YEARS AND OVER

Centre Name: Nottinghamshire Healthcare NHS Foundation Trust

REC reference: 18/NW/0079

Participant Identification Number for this trial:

CONSENT FORM

Title of Project: Online Remote Behavioural Intervention for Tics (ORBIT)

Name of Researcher:

Please initial box

1. I confirm that I have read the information sheet dated _____ (version___) for the above study. I have had the opportunity to consider the information, ask questions and have had these answered satisfactorily. ☐

2. I understand that my participation is voluntary and that I am free to withdraw at any time without giving any reason, without my medical care or legal rights being affected. ☐

3. I understand that relevant sections of my medical notes and data collected during the study, may be looked at by individuals from the ORBIT team, from regulatory authorities or from the NHS Trust, where it is relevant to my taking part in this research. I give permission for these individuals to have access to my records. I understand this data will be stored in the UK (Sealed Envelopes) and Sweden (BIP and BASS) secure databases and servers. ☐

4. I understand that the information collected about me will be used to support other research in the future, and may be shared anonymously with other researchers. ☐

5. I agree to my General Practitioner being informed of my participation in the study. ☐

For peer review only - http://bmjopen.bmj.com/site/about/guidelines.xhtml

Supplementary File B: Consent form for young people aged 16-years and over

6. I understand that I may be asked to take part in research interviews, which will be recorded and anonymous direct quotes from these interviews may be used in study reports. ☐

7. I agree to take part in the above study. ☐

For peer review only

Name of Participant	Date	Signature
Name of Person taking consent	Date	Signature

When completed: 1 for participant; 1 for researcher site file; 1 (original) to be kept in medical notes.

This research was funded by the NIHR Health Technology Assessment (ref 16/19/02). The views expressed are those of the author(s) and not necessarily those of the NHS, the NIHR or the Department of Health.

For peer review only - http://bmjopen.bmj.com/site/about/guidelines.xhtml

Supplementary File C: Assent form for young people aged under 16 years

ASSENT FORM FOR YOUNG PEOPLE UNDER 16 YEARS

Centre Name: Nottinghamshire Healthcare NHS Foundation Trust

REC reference: 18/NW/0079

Participant Identification Number for this trial:

ASSENT FORM

Title of Project: Online Remote Behavioural Intervention for Tics (ORBIT)

Name of Researcher:

Please initial box

1. I have read the information sheet dated ______ (version___) for the ORBIT study. I have discussed it with my mum/dad/carer and the researcher and I have asked questions. ☐

2. I understand that I don't have to take part and I can stop taking part any time. This is my choice and no-one will be upset with me if I stop. ☐

3. I understand that the ORBIT team may look at my medical records and the data will be kept in a database both in England and in Sweden. This will be kept safe and only the research team will see my data. ☐

4. I understand that the research team will write a report about the project. My name will not be mentioned in any reports. ☐

5. I agree to my Doctor knowing that I am taking part in the ORBIT study. ☐

6. The researcher might ask me to take part in an interview about my experiences of the ORBIT trial. I do not have to take part. If I agree to take part, the interview will be recorded but only the research team will know that I did the interview. ☐

7. I agree to take part in the ORBIT study. ☐

For peer review only

For peer review only - http://bmjopen.bmj.com/site/about/guidelines.xhtml

Supplementary File C: Assent form for young people aged under 16 years

Name of young person	Date	Signature
Name of Person taking assent	Date	Signature

When completed: 1 for participant; 1 for researcher site file; 1 (original) to be kept in medical notes.

This research was funded by the NIHR Health Technology Assessment (ref 16/19/02). The views expressed are those of the author(s) and not necessarily those of the NHS, the NIHR or the Department of Health.

Funded by NIHR

For peer review only

For peer review only - http://bmjopen.bmj.com/site/about/guidelines.xhtml

SPIRIT 2013 Checklist: Recommended items to address in a clinical trial protocol and related documents*

Section/item	Item No	Description	Addressed on page number
**Administrative information**			
Title	1	Descriptive title identifying the study design, population, interventions, and, if applicable, trial acronym	___1_______
Trial registration	2a	Trial identifier and registry name. If not yet registered, name of intended registry	___4+10____
	2b	All items from the World Health Organization Trial Registration Data Set	____4+10___
Protocol version	3	Date and version identifier	_____26___
Funding	4	Sources and types of financial, material, and other support	____28+29____
Roles and responsibilities	5a	Names, affiliations, and roles of protocol contributors	___1+2____
	5b	Name and contact information for the trial sponsor	____26 + 29____
	5c	Role of study sponsor and funders, if any, in study design; collection, management, analysis, and interpretation of data; writing of the report; and the decision to submit the report for publication, including whether they will have ultimate authority over any of these activities	____26+28__
	5d	Composition, roles, and responsibilities of the coordinating centre, steering committee, endpoint adjudication committee, data management team, and other individuals or groups overseeing the trial, if applicable (see Item 21a for data monitoring committee)	___25+26____

For peer review only - http://bmjopen.bmj.com/site/about/guidelines.xhtml

**Introduction**			___________
Background and rationale	6a	Description of research question and justification for undertaking the trial, including summary of relevant studies (published and unpublished) examining benefits and harms for each intervention	__5-6+8____
	6b	Explanation for choice of comparators	____9+14_____
Objectives	7	Specific objectives or hypotheses	____8-9_____
Trial design	8	Description of trial design including type of trial (eg, parallel group, crossover, factorial, single group), allocation ratio, and framework (eg, superiority, equivalence, noninferiority, exploratory)	___9-10____
**Methods: Participants, interventions, and outcomes**			
Study setting	9	Description of study settings (eg, community clinic, academic hospital) and list of countries where data will be collected. Reference to where list of study sites can be obtained	___10_____
Eligibility criteria	10	Inclusion and exclusion criteria for participants. If applicable, eligibility criteria for study centres and individuals who will perform the interventions (eg, surgeons, psychotherapists)	____11____
Interventions	11a	Interventions for each group with sufficient detail to allow replication, including how and when they will be administered	____12-15__
	11b	Criteria for discontinuing or modifying allocated interventions for a given trial participant (eg, drug dose change in response to harms, participant request, or improving/worsening disease)	____NA_____
	11c	Strategies to improve adherence to intervention protocols, and any procedures for monitoring adherence (eg, drug tablet return, laboratory tests)	_____15_____
	11d	Relevant concomitant care and interventions that are permitted or prohibited during the trial	_____11_____
Outcomes	12	Primary, secondary, and other outcomes, including the specific measurement variable (eg, systolic blood pressure), analysis metric (eg, change from baseline, final value, time to event), method of aggregation (eg, median, proportion), and time point for each outcome. Explanation of the clinical relevance of chosen efficacy and harm outcomes is strongly recommended	15-21+Table 1
Participant timeline	13	Time schedule of enrolment, interventions (including any run-ins and washouts), assessments, and visits for participants. A schematic diagram is highly recommended (see Figure)	__Fig1,2, Table 1

For peer review only - http://bmjopen.bmj.com/site/about/guidelines.xhtml

Sample size	14	Estimated number of participants needed to achieve study objectives and how it was determined, including clinical and statistical assumptions supporting any sample size calculations	____22____
Recruitment	15	Strategies for achieving adequate participant enrolment to reach target sample size	__22___
**Methods: Assignment of interventions (for controlled trials)**			
Allocation:			___22-23_____
Sequence generation	16a	Method of generating the allocation sequence (eg, computer-generated random numbers), and list of any factors for stratification. To reduce predictability of a random sequence, details of any planned restriction (eg, blocking) should be provided in a separate document that is unavailable to those who enrol participants or assign interventions	___22-23_____
Allocation concealment mechanism	16b	Mechanism of implementing the allocation sequence (eg, central telephone; sequentially numbered, opaque, sealed envelopes), describing any steps to conceal the sequence until interventions are assigned	___22-23_____
Implementation	16c	Who will generate the allocation sequence, who will enrol participants, and who will assign participants to interventions	____22-23____
Blinding (masking)	17a	Who will be blinded after assignment to interventions (eg, trial participants, care providers, outcome assessors, data analysts), and how	____22-23____
	17b	If blinded, circumstances under which unblinding is permissible, and procedure for revealing a participant's allocated intervention during the trial	___22-23_____
**Methods: Data collection, management, and analysis**			
Data collection methods	18a	Plans for assessment and collection of outcome, baseline, and other trial data, including any related processes to promote data quality (eg, duplicate measurements, training of assessors) and a description of study instruments (eg, questionnaires, laboratory tests) along with their reliability and validity, if known. Reference to where data collection forms can be found, if not in the protocol	__15-21____
	18b	Plans to promote participant retention and complete follow-up, including list of any outcome data to be collected for participants who discontinue or deviate from intervention protocols	__23-24_____

For peer review only - http://bmjopen.bmj.com/site/about/guidelines.xhtml

Data management	19	Plans for data entry, coding, security, and storage, including any related processes to promote data quality (eg, double data entry; range checks for data values). Reference to where details of data management procedures can be found, if not in the protocol	___23-24_____
Statistical methods	20a	Statistical methods for analysing primary and secondary outcomes. Reference to where other details of the statistical analysis plan can be found, if not in the protocol	___23/25____
	20b	Methods for any additional analyses (eg, subgroup and adjusted analyses)	___24_____
	20c	Definition of analysis population relating to protocol non-adherence (eg, as randomised analysis), and any statistical methods to handle missing data (eg, multiple imputation)	___24_____
**Methods: Monitoring**			
Data monitoring	21a	Composition of data monitoring committee (DMC); summary of its role and reporting structure; statement of whether it is independent from the sponsor and competing interests; and reference to where further details about its charter can be found, if not in the protocol. Alternatively, an explanation of why a DMC is not needed	___25____
	21b	Description of any interim analyses and stopping guidelines, including who will have access to these interim results and make the final decision to terminate the trial	____9-10 + 25___
Harms	22	Plans for collecting, assessing, reporting, and managing solicited and spontaneously reported adverse events and other unintended effects of trial interventions or trial conduct	_____19+26____
Auditing	23	Frequency and procedures for auditing trial conduct, if any, and whether the process will be independent from investigators and the sponsor	____26______
**Ethics and dissemination**			
Research ethics approval	24	Plans for seeking research ethics committee/institutional review board (REC/IRB) approval	_____26-27___
Protocol amendments	25	Plans for communicating important protocol modifications (eg, changes to eligibility criteria, outcomes, analyses) to relevant parties (eg, investigators, REC/IRBs, trial participants, trial registries, journals, regulators)	____27_____

For peer review only - http://bmjopen.bmj.com/site/about/guidelines.xhtml

Consent or assent	26a	Who will obtain informed consent or assent from potential trial participants or authorised surrogates, and how (see Item 32)	____26-27____
	26b	Additional consent provisions for collection and use of participant data and biological specimens in ancillary studies, if applicable	____26-27____
Confidentiality	27	How personal information about potential and enrolled participants will be collected, shared, and maintained in order to protect confidentiality before, during, and after the trial	___27____
Declaration of interests	28	Financial and other competing interests for principal investigators for the overall trial and each study site	____27+29____
Access to data	29	Statement of who will have access to the final trial dataset, and disclosure of contractual agreements that limit such access for investigators	___27_______
Ancillary and post-trial care	30	Provisions, if any, for ancillary and post-trial care, and for compensation to those who suffer harm from trial participation	_NA (see p27)__
Dissemination policy	31a	Plans for investigators and sponsor to communicate trial results to participants, healthcare professionals, the public, and other relevant groups (eg, via publication, reporting in results databases, or other data sharing arrangements), including any publication restrictions	___26-27_____
	31b	Authorship eligibility guidelines and any intended use of professional writers	___27_____
	31c	Plans, if any, for granting public access to the full protocol, participant-level dataset, and statistical code	___NA______
**Appendices**			
Informed consent materials	32	Model consent form and other related documentation given to participants and authorised surrogates	___Appendix A,B,C_______
Biological specimens	33	Plans for collection, laboratory evaluation, and storage of biological specimens for genetic or molecular analysis in the current trial and for future use in ancillary studies, if applicable	___NA_______

*It is strongly recommended that this checklist be read in conjunction with the SPIRIT 2013 Explanation & Elaboration for important clarification on the items. Amendments to the protocol should be tracked and dated. The SPIRIT checklist is copyrighted by the SPIRIT Group under the Creative Commons "Attribution-NonCommercial-NoDerivs 3.0 Unported" license.

For peer review only - http://bmjopen.bmj.com/site/about/guidelines.xhtml
