# Supplementary material for: Investigating a therapist-guided, parent-assisted remote digital behavioural intervention for tics in children and adolescents—‘Online Remote Behavioural Intervention for Tics’ (ORBIT) trial: protocol of an internal pilot study and single-blind randomised controlled trial
Source: BMJ Open. 2019 Jan 3;9(1):e027583. doi: 10.1136/bmjopen-2018-027583 (PMC6326281; doi:10.1136/bmjopen-2018-027583)
Supplement: Reviewer comments [file bmjopen-2018-027583.reviewer_comments.pdf]

PEER REVIEW HISTORY

BMJ Open publishes all reviews undertaken for accepted manuscripts. Reviewers are asked to complete a checklist review form (**http://bmjopen.bmj.com/site/about/resources/checklist.pdf**) and are provided with free text boxes to elaborate on their assessment. These free text comments are reproduced below.

ARTICLE DETAILS

**TITLE (PROVISIONAL)**	Investigating a therapist-guided, parent-assisted remote digital behavioural intervention for tics in children and adolescents – 'Online Remote Behavioural Intervention for Tics' (ORBIT) Trial: protocol of an internal pilot study and single-blind randomised controlled trial
**AUTHORS**	Hall, Charlotte Lucy; Davies, E. Bethan; Andrén, Per; Murphy, Tara; Bennett, Sophie; Brown, Beverley J; Brown, Susan; Chamberlain, Liam; Craven, Michael; Evans, Amber; Glazebrook, Cristine; Heyman, Isobel; Hunter, Rachael; Jones, Rebecca; Kilgariff, Joseph; Marston, Louise; Mataix-Cols, David; Murray, Elizabeth; Sanderson, Charlotte; Serlachius, Eva; Hollis, Chris

This article was not externally reviewed at BMJ Open. Protocols that have been independently assessed prior to submission to BMJ Open are usually fast-tracked to publication on the grounds that further substantial changes will not be possible. This independent assessment will usually be external, independent review for both a substantial grant award from a non-commercial or government funder and ethics approval.
